# Exploring the Mechanism of Ammonium Dinitramide Synthesis through DFT Calculations

**DOI:** 10.1021/acsomega.5c06735

**Published:** 2025-08-30

**Authors:** Letícia M. S. V. Queiroz, Gabriel F. S. Fernandes, Josiane R. C. Silva, Francisco B. C. Machado, Luiz F. A. Ferrão

**Affiliations:** Department of Chemistry, Aeronautics Institute of Technology (ITA), 12228-900 São José dos Campos, SP, Brazil

## Abstract

Ammonium dinitramide (ADN) has emerged as a promising substitute for ammonium perchlorate (AP) in solid rocket propellants due to its favorable energetic properties and absence of halogens. However, a comprehensive understanding of its synthesis mechanism has not been thoroughly achieved. In this sense, this study explores various submechanisms governing the synthesis of ADN in the gas phase and under the influence of an implicit solvent (PCM). Density Functional Theory (DFT) was employed to determine the thermochemical properties of all stationary states on the ground-state potential energy surface related to the elementary reactions involved in the nitration process. Five different pathways were described for the gas phase reaction and three different pathways with implicit solvent, all of which were accessible. Two of the five pathways (the same two pathways found with PCM) appear to be preferential, forming nitramide more easily and favoring the formation of dinitramidic acid (HDN), which will later react with ammonia to form ADN.

## Graphical abstract

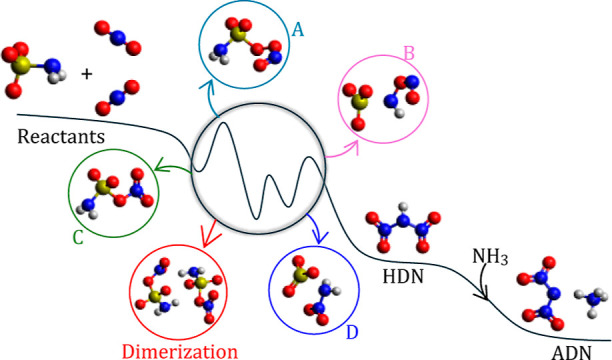

## Introduction

Oxidizers are typically the primary component by mass in propellant mixtures. To illustrate, consider the equation 2H_2_ + O_2_ → 2H_2_O, where H_2_ acts as the fuel and O_2_ as the oxidizer. The mass ratio between hydrogen and oxygen is 1/8, resulting in a fuel–oxidizer mixture composed of 11.1% fuel and 88.9% oxidizer. The precise ratios depend on the chosen combination of fuel and oxidizer; however, the oxidizer comprises a significant proportion of the propellant mixtures, irrespective of the specific molecules involved. For instance, in solid propellants, at least 70% of the total composition is comprised of the oxidizer,^1,2^ making it the most crucial component of a solid propellant.

One of the most widely used oxidizing agents in solid propellants is ammonium perchlorate (AP) which, despite its high efficiency, is also environmentally harmful^3–6^ since it releases a significant amount of HCl_(g)_,^7^ Cl_2(g)_, and other chlorinated compounds (ClO_*x*_) during its decomposition.^8^ This has led to a growing interest in new substitutes for AP. Among these, one possible substitute has emerged as a particularly promising candidate for use as an oxidizer in solid rocket propellant compositions, the ammonium dinitramide, ADN (Figure 1). ADN is a highly energetic and halogen-free oxidizer, capable of producing a higher specific impulse than AP.^9–12^

**Figure 1:**
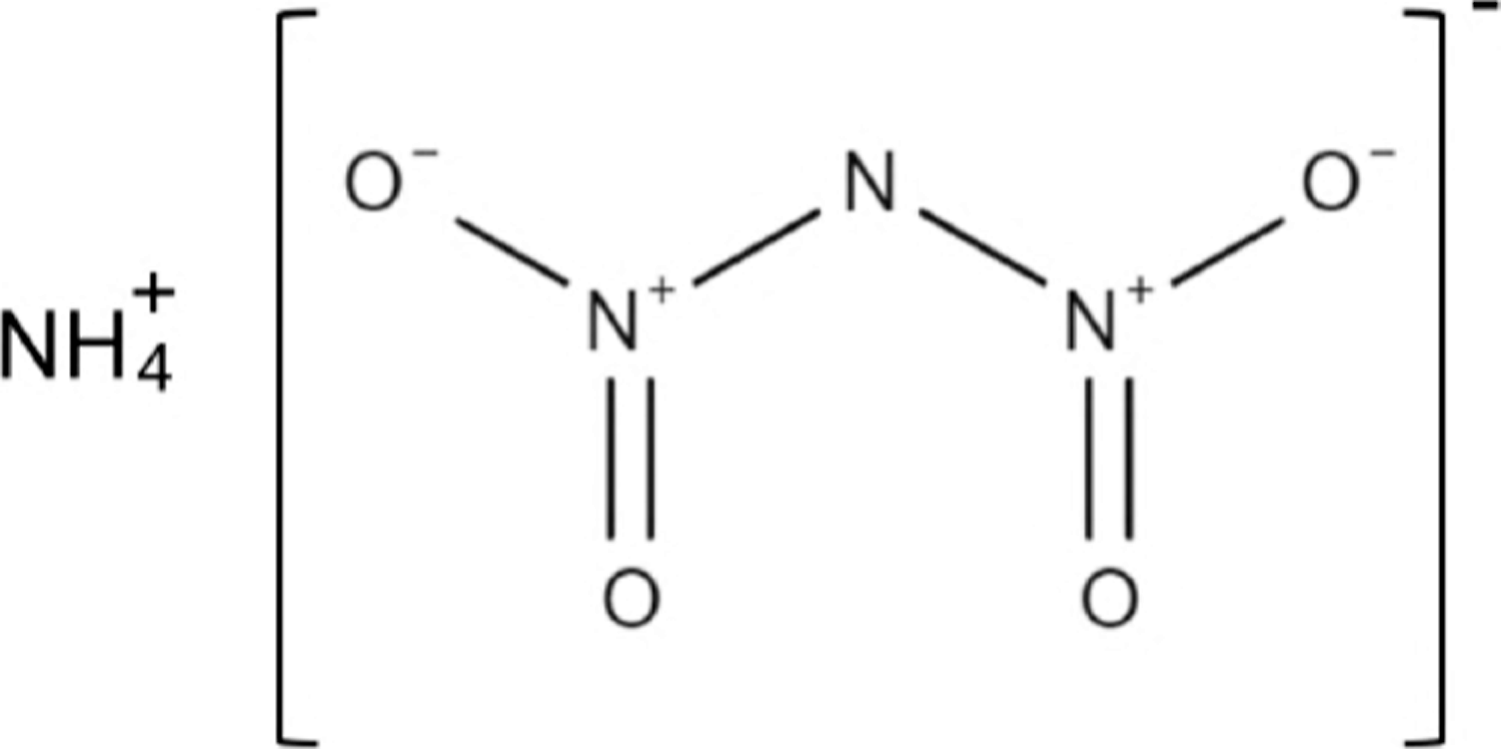
Ammonium dinitramide structure.

First synthesized in 1971 in URSS, and later in 1988 in the USA,^4,9,10^ ADN (Figure 1) is a high-density oxidant (1.81 g cm^–3^), with high enthalpy of formation (−125.3 kJ mol^–1^, compared to −283.1 kJ mol^–1^ of AP) and high positive oxygen balance (25.8%). However, despite its advantages, ADN also presents disadvantages, including costly synthesis, high hygroscopicity, and light and moisture sensitivity.^8^ Furthermore, when synthesized, ADN grows in a needle shape, forming a monoclinic crystalline system (*a* = 6.914 Å, *b* = 11.787 Å, *c* = 5.614 Å).^13,14^ The needle shape can impede the packing of the oxidant particles, leading to inappropriate mechanical behavior of the propellant, once it affects the final viscosity of the mixture and the propellant processing. Thus, the amount of oxidizer present in the formulation is also influenced by the shape of the oxidizer particles.^15^

Techniques for recrystallization and encapsulation can be used to mitigate issues related to particle packing, hygroscopicity, and sensitivity to the environment.^2,15,16^ Nevertheless, it is indispensable to conduct studies that aim to comprehend and enhance the synthesis process. Detailed information about the reaction mechanism in question is crucial for effective synthesis route planning.

Despite the progress in ADN research, most theoretical works focus on the electronic structure and vibrational properties of ADN,^17^ ADN crystallography,^18^ hygroscopicity,^11^ sublimation mechanisms,^14^ and ADN pyrolysis.^19^ Nevertheless, the mechanism of ADN synthesis is still not fully understood.^20^

There are, at least, 20 synthesis routes of ADN,^5^ but the most economically and safety-wise advantageous is the nitration of the sulfamate anion (NH_2_SO_3_^–^), normally obtained from a salt (e.g. potassium sulfamate), by the nitronium cation (NO_2_^+^),^15,21^ obtained either from a sulfonitric mixture, as shown by eq 1, or from nitronium salts, as shown by eq 2. To synthesize ADN, the NO_2_^+^ first reacts with NH_2_SO_3_^–^ in aqueous medium to form dinitramidic acid (HDN), an unstable compound. For this reason, HDN (HN­(NO_2_)_2_) reacts then with the ammonium ion to finally produce ADN (NH_4_N­(NO_2_)_2_) (eqs 3 and 4).^15^

1 $$H_{2}{\text{SO}}_{4}+{HNO}_{3}\Leftrightarrow {{NO}_{2}}^{+}+{{HSO}_{4}}^{-}+H_{2}O$$

2 $${NO}_{2}Y\Leftrightarrow {{NO}_{2}}^{+}+Y^{-}$$

3 $${NH}_{2}{{\text{SO}}_{3}}^{-}+2{{NO}_{2}}^{+}+H_{2}O\to HN{\left({NO}_{2}\right)}_{2}+{{HSO}_{4}}^{-}+2H^{+}$$

4 $$HN{\left({NO}_{2}\right)}_{2}+{{NH}_{4}}^{+}\to {NH}_{4}N{\left({NO}_{2}\right)}_{2}+H^{+}$$

As one can note, the main reaction in the ADN synthesis is that represented by eq 3. Nevertheless, the reaction represented by eq 3 only shows the initial reactants and final products that can be obtained experimentally by conventional characterization techniques. However, some questions are still not fully understood, for example, how the reactants interact with each other and what intermediates are formed? What are the main elementary reactions that build the whole mechanism? The answers for these questions are not trivial, especially because this reaction should involve several steps (elementary reactions), and some of them possibly involve charge transfer and ionic species. This work aims to explore the Potential Energy Surface (PES) around the minimum energy paths related to the ADN synthesis. This data can be utilized to enhance the description of the ADN synthesis mechanism and improve the planning of efficient synthesis routes. For this challenge, the reactants, some intermediate species, and the products involved in eq 3 were examined using Density Functional Theory (DFT) in the gas phase and applying the effect of a field that was implemented by an implicit solvent.

## Methodology

The thermochemical properties of the ADN synthesis reaction were obtained by characterizing the stationary states (SS), including reactants, products, and possible intermediates, through Density Functional Theory (DFT) within a hybrid *meta*-GGA approximation, M06-2X^22^ combined with the def2-SVP^23,24^ basis set. Frequency analysis was carried out to compute the thermodynamic quantities and to identify possible saddle points (defined in this work as transition states, TS) during the optimization. Thus, the intermediates (Figures S1 and S18) were classified as either minima, Stationary States (SS), or saddle point, Transition States (TS), based on the absence or presence of only one imaginary frequency vibrational mode, respectively (Tables S1 and S3). To ensure the connection between the SSs, Intrinsic Reaction Coordinate (IRC) calculations were performed for each of the previously characterized TS. If saddle points were not found, then the PES was scanned between the reactants and products. The electronic energies were corrected using single-point calculations with the M06-2X functional combined with the def2-TZVP basis set (Tables S2 and S4). Relative energies refer to electronic energy differences with respect to the reactants (SS0).

The reaction was first studied in the gas phase. Subsequently, the effect of an implicit solvent was included using the Polarizable Continuum Model (PCM).^25^ This makes it important to evaluate the effect of the electrostatic field on the reaction. The choice of PCM was made since in this model, the solute is placed within an electrostatic cavity encircled by a dielectric medium representing the solvent, and therefore, there would be no direct interaction between the solute and the solvent that might change the mechanism. Because highly polar substances, such as nitric and sulfuric acids, are expected to have a high dielectric constant, similar to water, water was used as an approximation for the solvent effect. It is evident that the energy of the SS as well as their relative energies may vary according to the selected solvent. However, the mechanism is expected to remain predominantly unchanged.

All the geometry optimizations and IRC were performed using the Gaussian 09 package,^26^ and the PES were performed using Gaussian 09 and Molpro 2015.^27^ All of the information on geometry, IRC, PES, frequencies, and corrected thermochemical properties can be accessed in the Supporting Information.

In the current route synthesis of ADN, two NO_2_^+^ ions react with one NH_2_SO_3_^–^ ion (eq 3); for this reason, in this work, we propose that the reaction occurs in two sequential steps. Initially, the NH_2_SO_3_^–^ ion interacts with one NO_2_^+^ ion. Subsequently, the second NO_2_^+^ ion interacts with the product of the previous step. In this context, eight initial guesses (G*n*, where *n* is the *n*-th guess) were generated by modifying the relative position of the first NO_2_^+^ around NH_2_SO_3_^–^. This will be discussed in greater detail in the Results and Discussion section.

Once HDN is equivalent to the substitution of one hydrogen atom by one nitro group in a nitramide, the main part of the reaction is to obtain nitramide. Once this species is obtained, it is easier to produce HDN. For this reason, as will be discussed in the Results and Discussion section, each of the proposed reaction pathways has been studied in order to produce nitramide or any other structure close to it.

All of the calculations were conducted aiming to reproduce, at least to some extent, the real environment in which the reaction is submitted in the laboratory. As previously stated in the Introduction section, the sulfamate anion is typically obtained from a salt (e.g., potassium sulfamate) and the nitronium from a sulfonitric mixture or from a nitronium salt. However, since these are the first data about the reaction itself, some modifications were necessary. It is therefore important to note that in this work, the influence of any counterion (e.g., K^+^ or Y^–^) and the influence of acidic pH were not evaluated.

The experimental route of the synthesis of ADN involves the neutralization of dinitramidic acid with ammonium hydroxide (NH_4_OH), resulting in the desired salt (ADN) and water. Since the acidic medium was not evaluated in this work, the last reaction step consisted of the addition of ammonia (NH_3_) instead of the ammonium ion (NH_4_^+^), according to eq 5

5 $$HN{\left({NO}_{2}\right)}_{2}+{NH}_{3}\to {NH}_{4}N{\left({NO}_{2}\right)}_{2}$$

## Results and Discussion

Upon the initial approach of the first NO_2_^+^ with NH_2_SO_3_^–^, it was observed that the interaction between both ions is intramolecular, as expected, with the NO_2_^+^ binding to the anion, independently of the initial distance between the ions and of the medium (gas phase or PCM). The different guess geometries led to the formation of four different SS1 in the gas phase and two different SS1 with PCM (Figure 2). From these SS1, we were able to explore five different pathways for the formation of HDN in the gas phase and three different pathways for the formation of HDN in with PCM.

**Figure 2:**
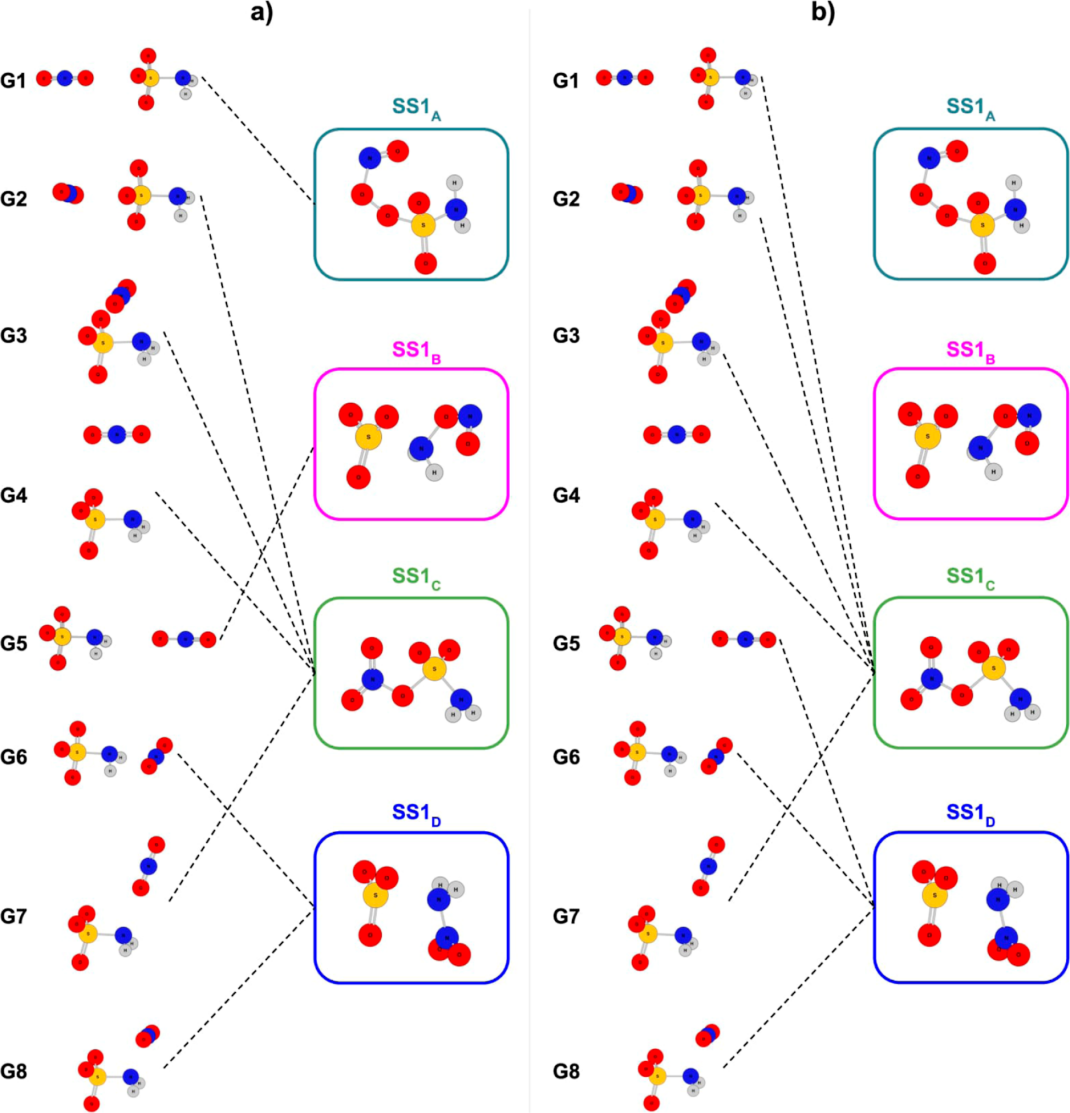
The figure shows the first SS obtained by approximating NO_2_^+^ and H_2_NSO_3_^–^ in gas phase (a) andunder implicit solvent (b). It worth noting that SS1_A_ and SS1_B_ obtained in the gas phase were reoptimized, adding the implicit solvent effect, and they exist in PES within the PCM; however, they were not found by means of the guesses for ions approximation. Calculated at M06-2X/def2-SVP.

The four SS1 structures were analyzed based on thermochemical properties, and it was verified that SS1_A_ is the least stable. This structure forms a bond between an oxygen atom and the NO_2_^+^ ion, creating a –O–O– bond. SS1_B_ is the second least stable structure and forms an intermolecular interaction between SO_3_ and H_2_N–ONO. SS1_c_ is the second most stable structure and has the formation of a N–O bond as its main feature. Due to the possibility of internal rotation of the dihedrals, it presents three conformational isomers, which are significantly accessible by overcoming the energy barriers of approximately 5 kcal mol^–1^ (Figure S10). Finally, SS1_D_ is the most stable SS1 found, which presents an intermolecular interaction between SO_3_ and H_2_N–NO_2_ (nitramide), in a structure similar to SS1_B_ but with a much higher intramolecular binding energy.

As previously stated, each of the four reaction pathways (A–D) was investigated to produce nitramide or a structure closely related to nitramide. However, SS1_D_ is already a nitramide, from which only one more nitration is necessary to obtain HDN. Thus, SS1_D_ is not only the most stable SS1 identified, but it is also the closest to the final product.

Additionally, we investigated the possibility of producing HDN via a dimerization mechanism. The proposed dimer was constructed by interacting the two SS1_C_ monomers in opposing directions, where the NO_2_ moiety of one monomer binds the NH_2_ of the other one. The dimer formed by this intermolecular interaction was designated as SS1_DIM_.

## Gas Phase Reaction

The energy diagram for the gas phase mechanism is shown in Figure 3, and the numeric values are presented in Table 1. The most stable initial stationary state (SS1_D_) is dissociated into SO_3_ and HN_2_–NO_2_ (SS2_D_), as shown in Figure S12, followed by the approach of the second NO_2_^+^ to form an intermolecular complex (SS3_D_) between H_2_N–NO_2_ and NO_2_^+^. From the SS3_D_, the NO_2_ binds to the nitramide, breaking the NH bond and forming the SS5_D_ structure through the transition state (TS4_D_). This process involves the abstraction of hydrogen from nitrogen to oxygen (Figure S7). The SS5_D_ is, in fact, the protonated HDN (HDN-H) SS. The final step is the removal of the proton, but to properly remove the proton, it is necessary to use an explicit water molecule as an artifact to abstract the hydrogen, producing the species H_3_O^+^ (Figure S14). Finally, we obtained SS6_D_ and SS7_D_ (HDN).

**Figure 3:**
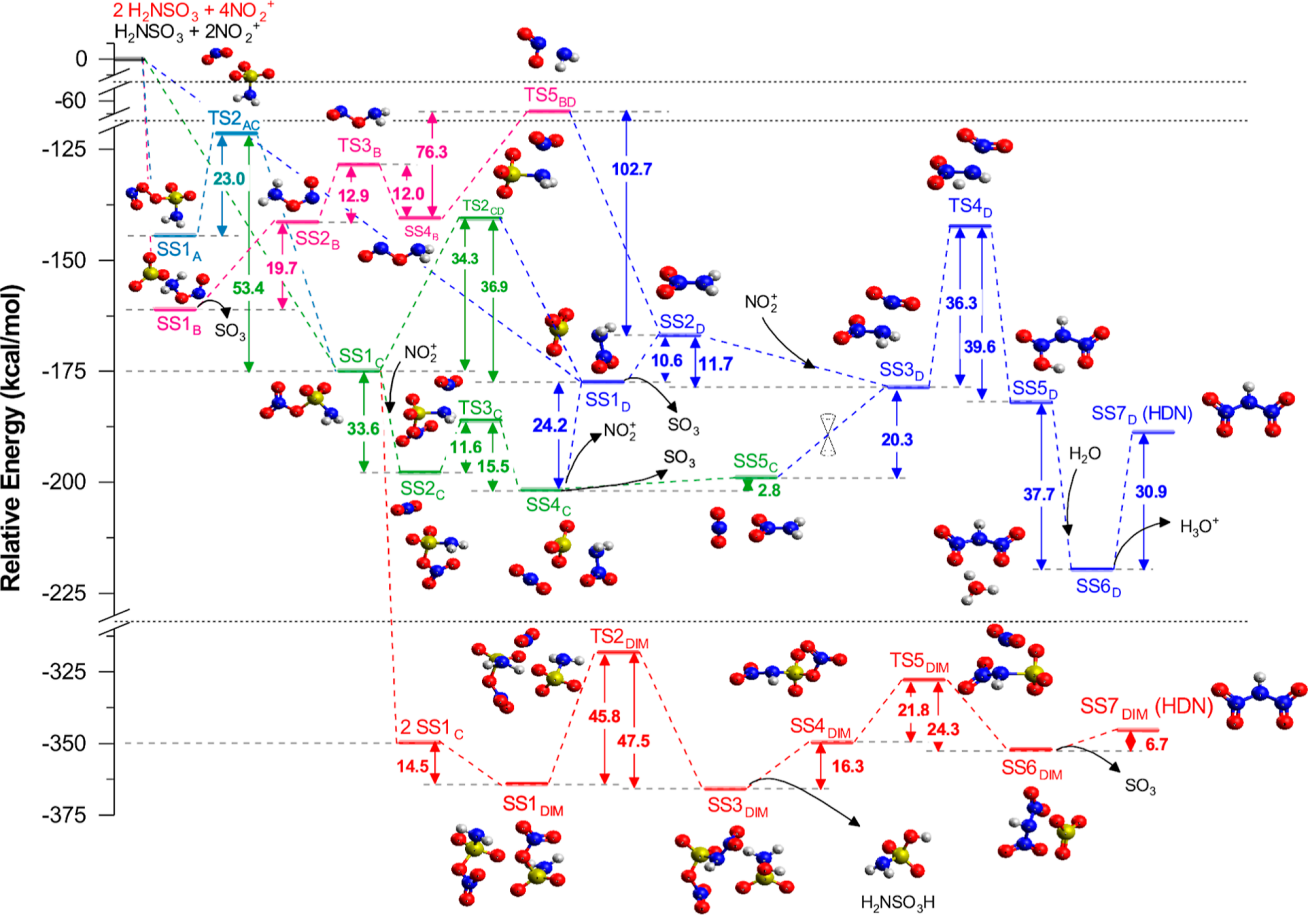
The figure shows the electronic energy diagram relative to reactants (SS0) calculated in the gas phase at M06-2X/def2-SVP. The different pathways, originated from each of the SS1 found (A, B, C, and D), are represented by different colors: in light blue is the A-pathway originated from SS1_A_, in pink is the B-pathway originated from SS1_B_, in green is the C-pathway originated from SS1_C_, in dark blue is the pathway originated from SS1_D_, and in red is represented the DIM-pathway (dimerization pathway) obtained from the dimerization of SS1_C_. All the structures (SS or TS) are also labeled with a number corresponding to their position in the respective pathway and with a subscripted letter emphasizing the pathway; for example, SS4_C_ is the fourth SS in the C-pathway. When a TS connects two different pathways, so it is labeled with both letters from both pathways; for example, TS2_CD_ is a transition state (TS) that connects both the pathways C and D. The double cone represents possible conical intersection (CI).

**Table 1:** Relative Energies (kcal mol^–1^) for the Gas Phase Reactions^a^

species	Δ*E*	Δ*H* (0 K)	Δ*H* (298.15 K)	Δ*G* (298.15 K)
SS1_A_	–144.38	–144.33	–144.80	–133.98
TS2_AC_	–121.38	–121.65	–121.98	–111.90
SS1_B_	–161.04	–160.39	–160.53	–151.38
SS2_B_	–141.38	–142.42	–142.36	–196.58
TS3_B_	–128.44	–130.45	–130.53	–132.62
SS4_B_	–140.47	–141.77	–141.57	–144.07
TS5_BD_	–64.16	–67.58	–67.50	–69.96
SS1_C_	–174.82	–173.19	–173.87	–162.79
TS2_CD_	–140.55	–139.85	–140.73	–129.12
SS2_C_	–197.80	–195.95	–196.34	–177.03
TS3_C_	–186.19	–186.19	–184.56	–165.31
SS4_C_	–201.68	–198.92	–199.26	–179.74
SS5_C_	–198.91	–198.14	–198.25	–191.19
SS1_D_	–177.47	–175.93	–176.04	–162.22
SS2_D_	–166.89	–166.65	–166.91	–168.64
SS3_D_	–178.58	–177.57	–177.74	–171.34
TS4_D_	–142.31	–144.38	–145.10	–137.17
SS5_D_	–181.91	–181.19	–182.18	–173.41
SS6_D_	–219.64	–216.24	–217.96	–199.15
SS7_D_	–188.75	–186.67	–187.74	–179.44
SS0_DIM_	–349.64	–346.37	–347.74	–325.58
SS1_DIM_	–362.80	–359.14	–359.99	–327.02
TS2_DIM_	–318.34	–317.85	–361.18	–284.32
SS3_DIM_	–365.82	–361.96	–362.75	–328.98
SS4_DIM_	–349.52	–347.09	–347.91	–327.40
TS5_DIM_	–327.77	–325.82	–326.88	–305.03
SS6_DIM_	–352.04	–350.00	–350.27	–331.65
SS7_DIM_	–345.30	–343.93	–344.64	–336.46

a Calculated by M06-2X/def2-SVP.

From the rotational properties of SS1_C_, it was investigated whether there might be a connection between SS1_C_ and SS1_D_. Indeed, a transition state (TS2_CD_) connecting the two SS was found (Figures S3 and S11). However, for SS1_C_ to reach SS1_D_, an energy barrier of 34.3 kcal mol^–1^ would have to be overcome. An alternative hypothesis to this reaction is the inclusion of the second NO_2_^+^ ion at SS1_C_, which would produce SS2_C_ and result in a reduction in energy by 33.6 kcal mol^–1^.

It can be reasonably presumed from SS2_C_ that the second NO_2_^+^ will bind with the −NH_2_ group to form nitramide, thus allowing SS2_C_ to pass through TS3_C_, leading to an intermolecular system (SS4_C_) containing SO_3_, NO_2_^+^, and H_2_N–NO_2_ (Figure S6). From this point, the system has two potential outcomes: it can dissociate NO_2_^+^ (24.2 kcal mol^–1^) to form SS1_D_ or it can dissociate SO_3_ to create an intermolecular system comprising (SS5_C_) of NO_2_^+^ and H_2_N–NO_2_ (only 2.8 kcal mol^–1^) (Figure S13). From SS5_C_, a rearrangement is observed, resulting in the formation of SS3_D_. This system consists of NO_2_ on top of the nitrogen from −NH_2_. It should be noted that these two systems (SS5_C_ and SS3_D_) are treated here as two SS. However, in order to connect these two SS, a TS or a Conical Intersection (CI) should exist. Since no TS was found, a possible CI is proposed. Nevertheless, it is possible to observe that the C-pathway will ultimately converge on the D-pathway, either for SS1_D_ or for SS3_D_.

Upon analysis of SS1_A_, it becomes evident that there is a certain degree of similarity to that of SS1_C_. However, there is a notable distinction between the two structures. In SS1_A_, the oxygen bound to sulfur is also bound to an oxygen from NO_2_ (O–O–S), rather than being bound to the nitrogen as it is in SS1_C_ (N–O–S). With this in mind, we investigated the possibility of these two structures being connected. Our findings revealed the existence of a transition state, TS2_AC_ (Figure S2), which connects the two structures. This transition state represents the breaking of the (–O–O–) bond to form a (–N–O–) bond.

SS1_B_ initially dissociates into SO_3_ + H_2_N–ONO (SS2_B_). As previously stated, this is a structure very similar to that of nitramide. Consequently, the subsequent steps should change the conformation to obtain the nitramide. To achieve this, it was first necessary to pass SS2_B_ through three other intermediates: TS3_B_ (Figure S4), SS4_B_, and TS5_BD_ (Figure S5).

Therefore, there are four distinct pathways, all of which are interconnected at some point, ultimately converging on the D-pathway. As previously mentioned, five different pathways have been studied in the gas phase, and so far, we have discussed only four of them. The fifth pathway consists of the dimerization of the first intermediary (SS1_C_). The formation of SS1_DIM_ decreases the energy of the system by −364.2 kcal mol^–1^ relative to that of SS0, a highly exothermic reaction.

From SS1_DIM_, it is possible to obtain a mechanism involving hydrogen abstraction. In this process, one SS1_C_ abstracts a hydrogen atom from the NH_2_ of another SS1_C_ to form H–OSO_3_. Concurrently, the nitro group is transferred from the second SS1_C_ to the first SS1_C_. This step requires 45.8 kcal mol^–1^, a high energy barrier that is recovered when SS3_DIM_ is formed (Figure S8). SS3_DIM_ is comprised of two intermolecular fragments, one of which is the sulfamic acid (NH_2_SO_3_H) and the other of which will be utilized to produce HDN. The reaction then proceeds with the dissociation of these two fragments to form SS4_DIM_, requiring 16.3 kcal mol^–1^ (Figure S15) and releasing the NH_2_SO_3_H in the medium.

From this stationary state (SS4_DIM_), a few conformational changes are required to produce HDN. It is necessary for the bond between NO_2_ and SO_3_ to be broken and for NO_2_ to bind to NH, overcoming an energy barrier of 21.8 kcal mol^–1^. This results in the formation of SS6_DIM_, an intermolecular interaction between HDN and SO_3_ (Figure S9). Finally, the last reaction step is dissociation of the sulfur trioxide molecule (SO_3_), which requires 6.7 kcal mol^–1^ and leads to the formation of the final product (Figure S16).

It is noteworthy that in the dimerization pathway, sulfamic acid (NH_2_SO_3_H) is released into the medium, which could be utilized as a precursor for the reaction as a sulfamate ion supplier. For the dimerization to occur, two SS1_C_ molecules must be present. Therefore, SS0 must consist of two sulfamate ions and four nitronium ions. When NH_2_SO_3_H is released in the medium, there would be two more NO_2_^+^ that could react with the sulfamate ion provided by NH_2_SO_3_H, continuing the reaction through one of the other four pathways studied in this work (A, B, C, or D).

Once HDN was produced, ammonia was added to produce ADN. This process did not present a saddle point, and therefore, it was scanned in terms of the two most significant coordinates, as shown in Figure 4. Large values of R_1_ and small values of R_2_ represent a covalent dissociation channel (HDN + NH_3_), while small R_1_ and large R_2_ represent the ionic dissociation channel (DN^–^ + NH_4_^+^).

**Figure 4:**
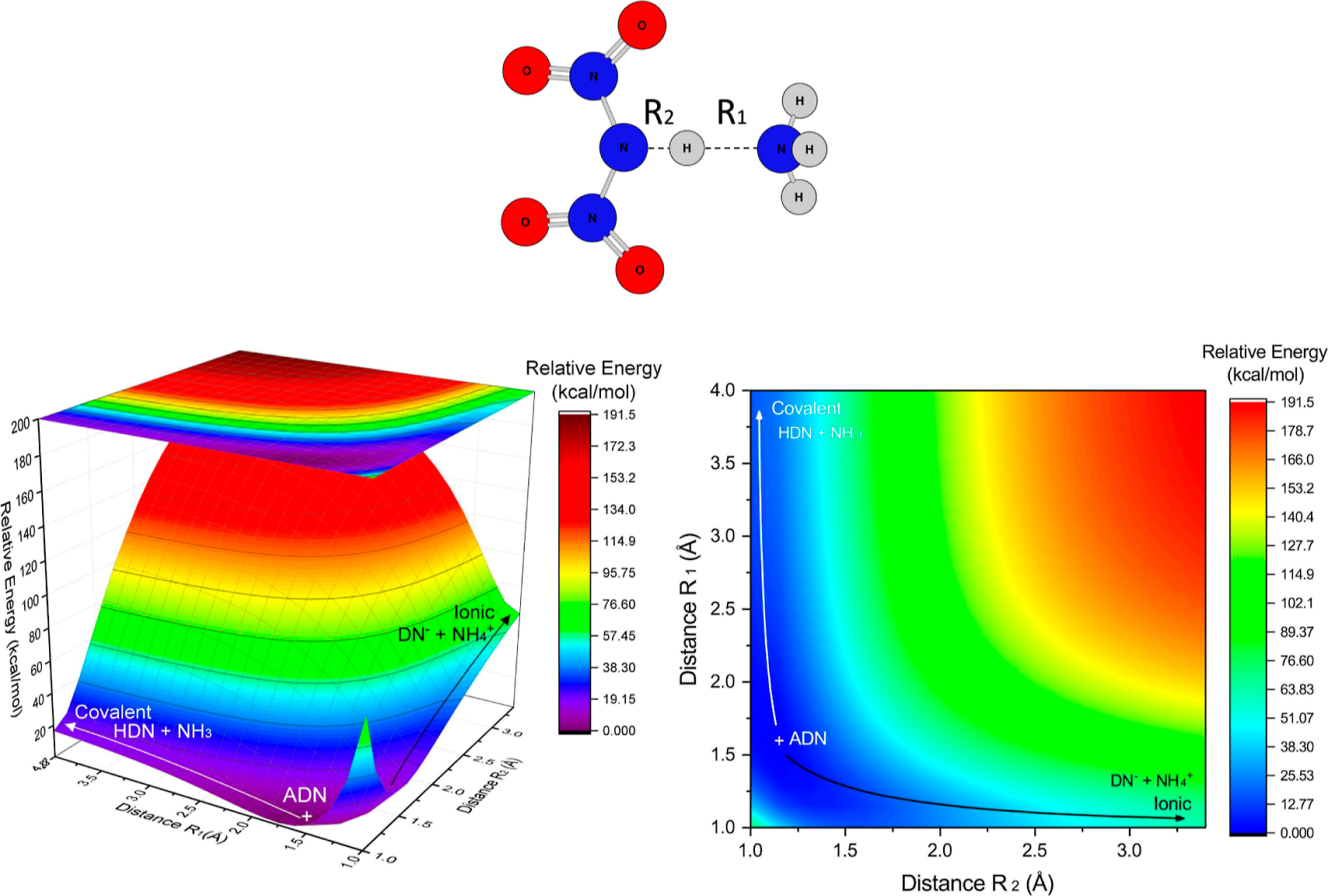
Rigid PES scan of ADN dissociation channels varying the distances R_1_ and R_2_. In the left side, one can see the three-dimensional visualization and in the right side the two-dimensional visualization of the surface. Calculated by M06-2X/def2-SVP.

The incorporation of ammonia into HDN to form ADN stabilizes the system by approximately 20 kcal mol^–1^ (Figure 4), in agreement with the optimized value (Table 2, also labeled as Figure S17 in the Supporting Information). From ADN, dissociation to the ionic channel requires more than 75 kcal mol^–1^.

**Table 2:** Dissociation Channels (kcal mol^–1^) Relative to ADN for the Gas Phase Reaction Calculated by M06-2X/def2-SVP

species	Δ*E*	Δ*H* (0 K)	Δ*H* (298.15 K)	Δ*G* (298.15 K)
HDN + NH_3_	18.42	17.35	18.06	9.04
DN^–^ + NH_4_^+^	124.10	124.13	124.59	115.26

The thermochemical data for the two endothermic dissociation channels are presented in Table 2. Based on the positive values of the calculated free energy, it can be concluded that the chemical equilibrium is shifted toward the formation of ADN rather than the dissociative products.

## Reaction under the Implicit Solvent Effect

The inclusion of PCM resulted in some differences in the mechanism. First, as mentioned above, only two SS1 were found (SS1_C_ and SS1_D_) out of the 8 guesses for the approximation of the ions, which leads to only 3 pathways to be studied (C, D, and the SS1_C_ dimerization pathway). The IRC connecting C and D pathways by means of TS2_CD_ and the potential energy curve (PEC) representing the dissociation of SS1_D_ are represented in Figures S19 and S27, respectively.

Second, from the diagram shown in Figure 5 and Table 3, it is possible to notice a much smaller variation in the relative electronic energy. While in the gas phase, the C and D pathways were about −150 kcal mol^–1^ relative to SS0, with PCM, this difference is reduced to −50 kcal mol^–1^ relative to SS0. The same is observed in the dimerization pathway, while in gas, this pathway occurs at about −350 kcal mol^–1^, and with PCM, this difference is reduced to −90 kcal mol^–1^. In this sense, the mechanism when calculated with PCM releases much less heat than in the gas phase.

**Figure 5:**
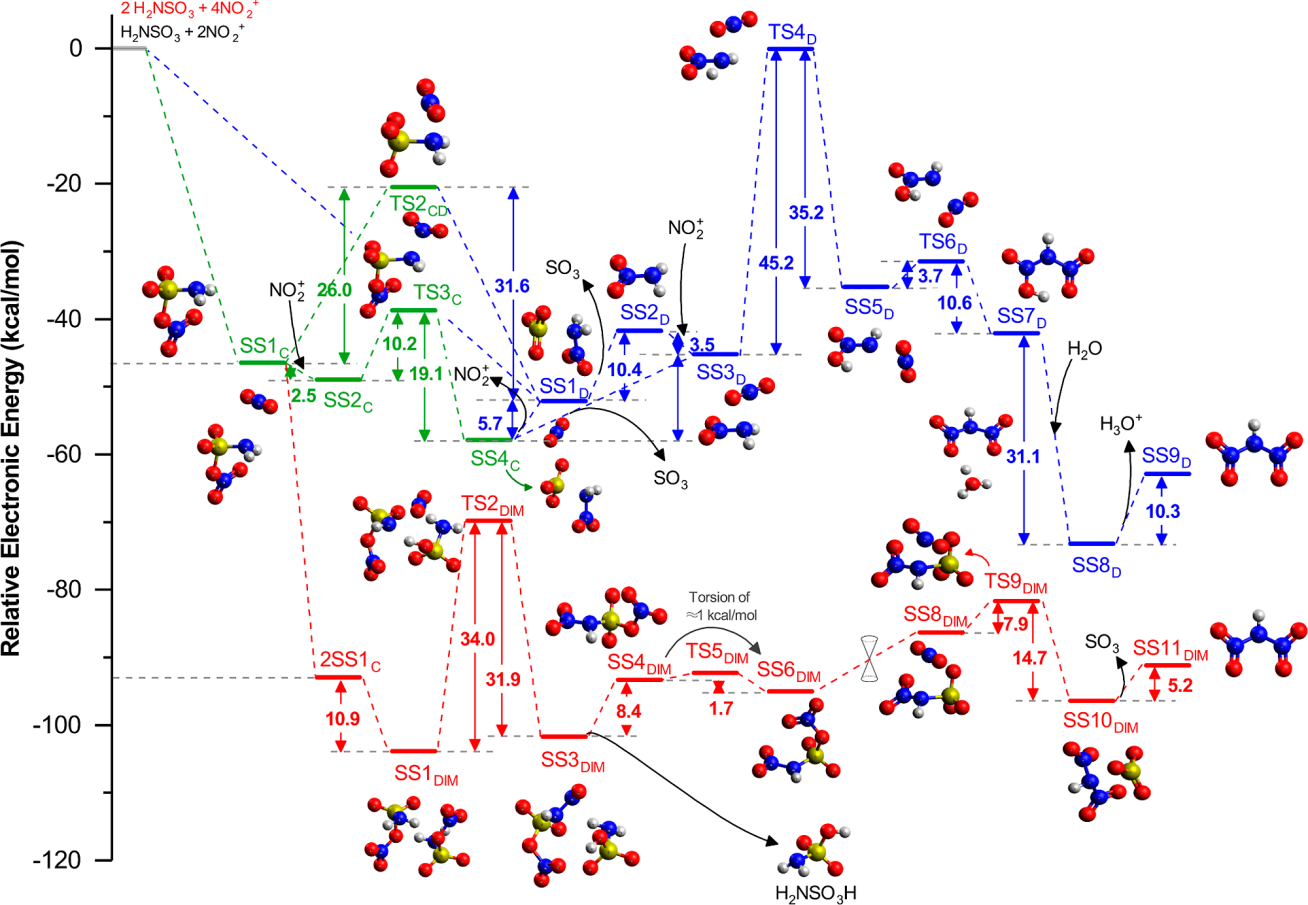
The figure shows the electronic energy diagram relative to reactants (SS0) for the reaction implicit solvent, calculated at M06-2X/def2-SVP/PCM. The different pathways, originated from each of the SS1 found (C and D), are represented by different colors: in green is the C-pathway originated from SS1_C_, in dark blue is the pathway originated from SS1_D_, and in red is represented the DIM-pathway (dimerization pathway) obtained from the dimerization of SS1_C_. All the structures (SS or TS) are also labeled with a number corresponding to their position in the respective pathway and with a subscripted letter emphasizing the pathway; for example, SS4_C_ is the fourth SS in the C-pathway. When a TS connects two different pathways, so it is labeled with both letters from both pathways; for example, TS2_CD_ is a transition state (TS) that connects both the pathways C and D. The double cone represents possible conical intersection (CI).

**Table 3:** Relative Energies (kcal mol^–1^) for Reactions Calculated Under Implicit Solvent Using M06-2X/def2-SVP/PCM

species	Δ*E*	Δ*H* (0 K)	Δ*H* (298.15 K)	Δ*G* (298.15 K)
SS1_C_	–46.46	–45.24	–45.83	–34.94
TS2_CD_	–20.51	–20.01	–20.56	–9.79
SS2_C_	–48.96	–46.90	–47.24	–28.26
TS3_C_	–38.74	–37.15	–38.03	–17.66
SS4_C_	–57.85	–55.61	–56.13	–37.23
SS1_D_	–52.12	–50.38	–50.62	–41.25
SS2_D_	–41.70	–41.78	–41.95	–43.88
SS3_D_	–45.20	–44.31	–44.34	–19.06
TS4_D_	–0.05	–2.70	–2.77	3.13
SS5_D_	–35.23	–34.50	–34.52	–28.59
TS6_D_	–31.50	–31.45	–32.02	–24.51
SS7_D_	–42.08	–41.40	–42.43	–33.33
SS8_D_	–73.21	–69.98	–71.88	–52.62
SS9_D_	–62.88	–60.75	–61.70	–54.26
2SS1_C_	–92.92	–90.48	–91.66	–69.88
SS1_DIM_	–103.86	–100.80	–101.51	–67.97
TS2_DIM_	–69.84	–68.52	–69.18	–36.09
SS3_DIM_	–101.70	–99.05	–99.43	–66.79
SS4_DIM_	–93.30	–91.55	–92.22	–71.91
SS6_DIM_	–95.03	–92.99	–93.80	–72.96
SS8_DIM_	–86.34	–84.55	–84.80	–65.39
TS9_DIM_	–81.65	–80.29	–81.06	–60.14
SS10_DIM_	–96.39	–94.95	–95.05	–76.87
SS11_DIM_	–91.16	–90.52	–91.01	–83.91

Third, the electronic barriers between the intermediates are higher or lower, depending on the reaction step, when compared to those of the gas phase calculations. For example, for the D-pathway in the gas phase, in order to produce H-HDN (SS5_D_), the hydrogen abstraction of SS3_D_ requires an electronic barrier of 36.3 kcal mol^–1^ to be overcome, while the same process with PCM showed an increase of about 10 kcal mol^–1^ in the electronic barrier, in addition to requiring an extra step for the formation of H-HDN (Figures S21 and S22). In this sense, with PCM, the hydrogen is first abstracted and then the NO_2_^+^ is bonded. In the gas phase, these two steps take place simultaneously.

Despite the high electronic barriers, changing the solvent used in the PCM may vary the relative energy of the SS, keeping the mechanism essentially the same, as suggested in the Methodology section. Another way to reduce the energy barriers could be the inclusion of water molecules explicitly once H_2_O can make hydrogen bonds and help specially in the reduction of the barriers related to the hydrogen abstraction. However, the addition of explicit water molecules must be done with caution to avoid steering the mechanism toward unintended pathways since NO_2_^+^ in the presence of H_2_O can act as a Brønsted-base and produce HNO_3_, which is not desired. A more detailed discussion over the addition of *n*H_2_O (*n* = 1, 2, and 4) explicitly can be found in the Supporting Information (Section 3).

Another example of these electronic barrier differences is observed in the dimerization pathway. When calculated in the gas phase, hydrogen abstraction (TS2_DIM_) has to overcome a barrier of 46 kcal mol^–1^, which is probably the determinant step of this pathway. However, when the same calculation is performed with an implicit solvent, this electronic barrier is reduced by approximately 12 kcal mol^–1^. In the Supporting Information, one can find the IRC related to TS2_DIM_ labeled as Figure S23. Figure S23 shows only an energy barrier of 6.7 kcal mol^–1^ due to the fact that the IRC did not reach the true minimum for the reactant, but when optimizing it, the energy is reduced even more.

Finally, the structural conformations of some intermediates are different in gas and with PCM, showing the importance of the presence of the solvent and the role of the solvation layer. For example, the SS2_C_ in gas phase presents the NO_2_^+^ near to the SO_3_ group, while with PCM, the NO_2_^+^ is stabilized on top of the nitrogen of the NH_2_ group. For the SS4_C_ in the gas phase, the NO_2_^+^ is placed near the NO_2_ group of nitramide, while with PCM, it is near the NH_2_ group, favoring the approximation of the NO_2_^+^ to nitramide, and therefore, there is no SS5_C_ with PCM. For this reason, from SS4_C_, the system can more easily reach SS3_D_ when the reaction occurs under the solvent effects. The IRC related to TS3_C_, the PEC representing the approximation of NO_2_^+^ from SS1_C_ to form SS2_C_, passing through TS3_C_ and reaching SS4_C_, as well as the PEC to represent SS4_C_ dissociation, can be found in Figures S20, S25, and S26, respectively.

Also, comparing SS3_D_ obtained with the calculations in gas and with PCM, it is possible to notice that the solvation layer is capable of stabilizing the charge up to a higher step in the surface since at this point, the NO_2_ is still linear and, therefore, we still have NO_2_^+^, different from the gas phase process.

Once H-HDN was obtained, even though the implicit solvent was used, it was still necessary to incorporate an explicit water molecule to properly remove the proton and finally produce HDN (Figure S28).

For the dimerization pathway, there are also some other important differences from those in the gas phase. SS3_DIM_ is slightly less energetic than SS1_DIM_; in the gas phase, it is the opposite. Moreover, in the process of breaking the NO_2_ bond with SO_3_ and binding to NH_2_, first, the molecule SS4_DIM_ is formed, which undergoes a rotation of the SO_3_ group, overcoming a very low barrier of about 1 kcal mol^–1^ (estimate obtained from scanning the PES), which is in the “resolution limit” of our methodology (the chemical accuracy). This conformational change leads to SS6_DIM_ (Figure S30). From this structure, it was expected that the reaction would proceed exactly as in the gas phase (to SS7—HDN), but a new different SS was found, with the NO_2_^+^ stabilized on top of nitrogen (SS8_DIM_). However, no unrestrained minimum energy pathway (including saddle points) between SS6_DIM_ and SS8_DIM_ was found. As can be seen, the NO_2_ group changes from angular to linear, indicating a charge transfer. This also suggests a possible conical intersection (CI) between these two SS, but this could not be verified. Then, the reaction proceeds as in the gas phase, with the NO_2_ group passing through a TS (TS9_DIM_) and binding to nitrogen, forming an intermolecular system between SO_3_ and HDN (SS10_DIM_) and finally dissociating the SO_3_ to obtain the HDN (Figures S24 and S29).

Analogous to the gas phase calculation, once HDN was produced, ammonia was added to produce ADN. This process did not present a saddle point and was therefore scanned in terms of the two most significant coordinates, as shown in Figure 6, also labeled as Figure S31 in the Supporting Information. Large values of R_1_ and small values of R_2_ represent a covalent dissociation channel (HDN + NH_3_), while small R_1_ and large R_2_ represent the ionic dissociation channel (DN^–^ + NH_4_^+^).

**Figure 6:**
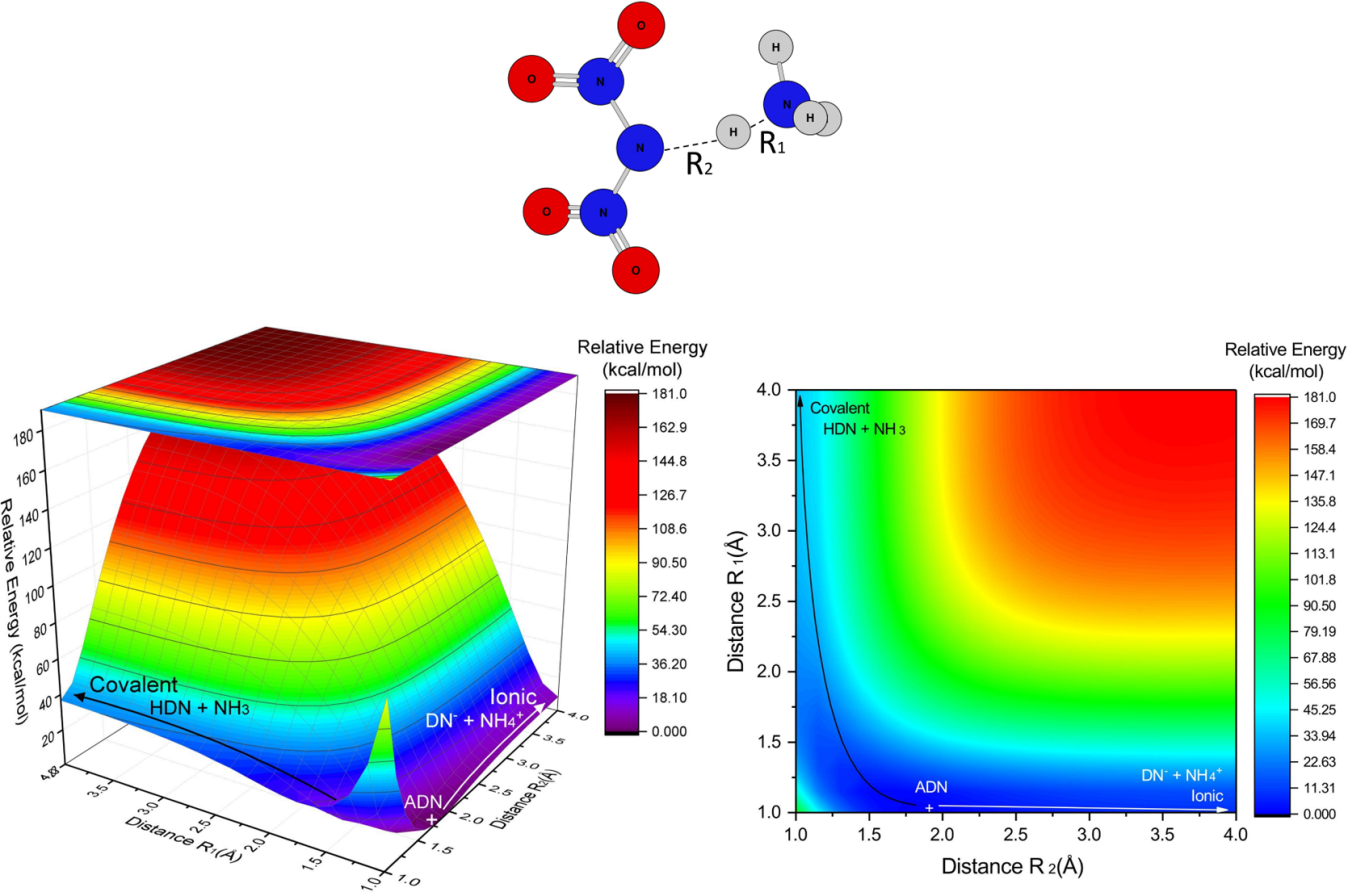
Rigid PES scan of ADN dissociation channels varying the distances R_1_ and R_2_. In the left side, one can see the three-dimensional visualization and in the right side the two-dimensional visualization of the surface. Calculated at M06-2X/def2-SVP/PCM.

In contrast to what happens in the gas phase, it is clear from Figure 6 that the incorporation of ammonia into the HDN to form ADN stabilizes the system by about 40 kcal mol^–1^ (although the optimization keeps this relative energy at about 20 kcal mol^–1^, as shown in Table 4), and the ionic dissociation channel has its relative energy drastically reduced to about 10 kcal mol^–1^, which makes sense once ADN is a soluble salt in water, and the solvent used within PCM approximation is water.

**Table 4:** Dissociation Channels (kcal mol^–1^) Relative to ADN for Calculations under Implicit Solvent Using M06-2X/def2-SVP/PCM

species	Δ*E*	Δ*H* (0 K)	Δ*H* (298.15 K)	Δ*G* (298.15 K)
HDN + NH_3_	24.60	22.35	22.79	13.32
DN^–^ + NH_4_^+^	13.92	13.21	13.34	4.27

## Conclusion

In the present work, we have described five possible pathways for ADN synthesis in the gas phase and three possible pathways for ADN synthesis under the implicit solvent effect (PCM). In all cases, the first reaction step involves the interaction of a sulfamate ion with a nitronium ion, which has been shown to be shock section dependent, producing 4 and 2 stationary states (SS1) in gas and with implicit solvent, respectively, which will later produce nitramide, making it a very important intermediate. In both cases, all of the SS 1s found are correlated with each other, since they all eventually converge on one of the four pathways (D-pathway). Also, in both gaseous and liquid systems, a dimerization submechanism has been proposed from one of the SS 1s found (SS1_C_). In this dimerized mechanism, the barriers are also significantly lowered and the bottleneck step occurs much earlier (TS2_DIM_) when comparing to the monomeric mechanism (TS4_D_).

The implementation of implicit solvent has also played an important role: it increased the electronic energy of the system by approximately 100 kcal mol^–1^, relative to the values calculated in the gas phase, in addition to helping stabilize the charge over NO_2_^+^, and led to additional steps to complete the reaction. However, the barriers corresponding to hydrogen abstraction (TS4_D_ and TS2_DIM_), even with implicit solvent, remained high, about 40 kcal mol^–1^, although the change in the chosen solvent may reduce the energy barriers, keeping the mechanism essentially the same.

In the gas phase, a missing connection between SS5_C_ and SS3_D_ was observed. We suggest that this missing intermediate could be a consequence of a conical intersection, instead of a simple transition state, once no TS was found. The same happens in the aqueous system between SS6_DIM_ and SS8_DIM_. Since the present work aims to describe the mechanism in its ground state by DFT calculations, the characterization of these intermediates was not evaluated.

To correct the thermodynamic functions, single-point calculations were performed with M06-2X/def2-TZVP (the corresponding data can be found in the Supporting Information). The energy correction kept the energy profile of the gas phase system essentially the same as that calculated with only M06-2X/def2-SVP alone, with only small variations in the relative energies. However, when analyzing the system under implicit solvent, the single-point calculations with M06-2X/def2-TZVP provided a higher energy barrier in a specific step: addition of NO_2_^+^ on SS1_C_ to form SS2_C_, passing through TS3_C_ and reaching SS4_C_. This divergence in values can be attributed to a possible difficulty of the PCM in representing the system when subjected to a change of base (optimization with a double-ζ and energy correction with a triple-ζ) since once in the PCM, the molecule is found in a cavity essentially described by the van der Waals radius. A smaller base, such as def2-SVP, may not be diffuse enough to occupy the entire cavity, generating a kind of vacuum that can be reduced by increasing the basis set and finally provoking different values in energy. Also, for the production of ADN from HDN, the implementation of solvent has played a very important role in describing the ionic dissociation energy, besides proving the higher stability of ADN compared to that of HDN.

## Supplementary Material



## Notes

Structures of all stationary and transition states, IRC, PES, thermochemistry data, and frequencies (PDF)

## References

[1] Agrawal, J. P. High Energy Materials, 1st ed.; Wiley-VCH Verlag: Weinheim, Germany, 2010.

[2] Silva J. O., Cardoso K. P., Silva J. R. C., Kawachi E. Y., Nagamachi M. Y., Ferrão L. F. A. (2020). ADN
Recrystallization and Microencapsulation with HTPB
by Simple Coacervation. Propellants, Explos.,
Pyrotech..

[3] Beckstead M. W., Puduppakkam K., Thakre P., Yang V. (2007). Modeling of Combustion
and Ignition of Solid-Propellant Ingredients. Prog. Energy Combust. Sci..

[4] Venkatachalam S., Santhosh G., Ninan Ninan K. (2004). An Overview
on the Synthetic Routes
and Properties of Ammonium Dinitramide (ADN) and other Dinitramide
Salts. Propellants, Explos., Pyrotech..

[5] Chen F.-Y., Xuan C.-L., Lu Q.-Q., Xiao L., Yang J.-Q., Hu Y.-B., Zhang G.-P., Wang Y.-L., Zhao F.-Q., Hao G.-Z., Jiang W. (2023). A Review on the High
Energy Oxidizer
Ammonium Dinitramide: Its Synthesis, Thermal Decomposition, Hygroscopicity,
and Application in Energetic Materials. Def.
Technol..

[6] Chaturvedi S., Dave P. N. (2019). Solid propellants:
AP/HTPB composite propellants. Arabian J. Chem..

[7] Gadiot G., Mul J., Meulenbrugge J., Korting P., Schnorkh A., Schöyer H. (1993). New solid
propellants based on energetic binders and HNF. Acta Astronaut..

[8] Kumar P. (2018). An Overview
on Properties, Thermal Decomposition, and Combustion Behavior of ADN
and ADN Based Solid Propellants. Def. Technol..

[9] Bottaro J. C., Penwell P. E., Schmitt R. J. (1997). 1,1,3,3-Tetraoxo-1,2,3-triazapropene
Anion, a New Oxy Anion of Nitrogen: The Dinitramide Anion and Its
Salts. J. Am. Chem. Soc..

[10] Christe K. O., Wilson W. W., Petrie M. A., Michels H. H., Bottaro J. C., Gilardi R. (1996). The Dinitramide Anion,N­(NO_2_)_2_
^–^. Inorg. Chem..

[11] Chen X., He L., Li X., Zhou Z., Ren Z. (2019). Molecular Simulation
Studies on the Growth Process and Properties of Ammonium Dinitramide
Crystal. J. Phys. Chem. C.

[12] Rahm M., Tyrode E., Brinck T., Johnson C. M. (2011). The Molecular
Surface
Structure of Ammonium and Potassium Dinitramide: A Vibrational Sum
Frequency Spectroscopy and Quantum Chemical Study. J. Phys. Chem. C.

[13] Gilardi R., Flippen-Anderson J., George C., Butcher R. J. (1997). A New Class of Flexible
Energetic Salts: The Crystal Structures of the Ammonium, Lithium,
Potassium, and Cesium Salts of Dinitramide. J. Am. Chem. Soc..

[14] Zhu R. S., Chen H.-L., Lin M. C. (2012). Mechanism
and Kinetics for Ammonium
Dinitramide (ADN) Sublimation: A First-Principles Study. J. Phys. Chem. A.

[15] Nagamachi M. Y., Oliveira J. I. S., Kawamoto A. M., Dutra R. D. C. L., Dutra R. (2009). ADN - The new oxidizer
around the corner for an environmentally friendly
smokeless propellant. J. Aerosp. Technol. Manage..

[16] Heintz T., Pontius H., Aniol J., Birke C., Leisinger K., Reinhard W. (2009). Ammonium Dinitramide
(ADN) - Prilling, Coating, and
Characterization. Propellants, Explos., Pyrotech..

[17] Zhu W., Wei T., Zhu W., Xiao H. (2008). Comparative DFT Study
of Crystalline
Ammonium Perchlorate and Ammonium Dinitramide. J. Phys. Chem. A.

[18] Lan Y., Zhai J., Li D., Yang R. (2015). The influence of solution
chemistry on the morphology of ammonium dinitramide crystals. J. Mater. Sci..

[19] Wang K., Xue B., Chen J.-G., He Z.-H., Ji Y., Wang B., Lu J., Liu Z.-W., Liu Z.-T. (2020). A combined experimental and theoretical
study of the thermal decomposition mechanism and kinetics of ammonium
dinitramide (ADN). New J. Chem..

[20] Lu Q., Chen F., Xiao L., Yang J., Hu Y., Zhang G., Zhao F., Wang Y., Jiang W., Hao G. (2022). Advances in the molecular simulation and numerical calculations of
the green high-energy oxidant ADN. Mater. Today
Commun..

[21] Jadhav P. M., Pandey R. K., Kulkarni A. A. (2021). Estimation
of reaction kinetics for
aromatic and heterocycles nitration in mixed acids through computational
chemistry approach. Int. J. Chem. Kinet..

[22] Zhao Y., Truhlar D. G. (2008). The M06 suite of density functionals for main group
thermochemistry, thermochemical kinetics, noncovalent interactions,
excited states, and transition elements: two new functionals and systematic
testing of four M06-class functionals and 12 other functionals. Theor. Chem. Acc..

[23] Weigend F., Ahlrichs R. (2005). Balanced basis sets
of split valence, triple zeta valence
and quadruple zeta valence quality for H to Rn: Design and assessment
of accuracy. Phys. Chem. Chem. Phys..

[24] Weigend F. (2006). Accurate Coulomb-fitting
basis sets for H to Rn. Phys. Chem. Chem. Phys..

[25] Marenich A. V., Olson R. M., Kelly C. P., Cramer C. J., Truhlar D. G. (2007). Self-Consistent
Reaction Field Model for Aqueous and Nonaqueous Solutions Based on
Accurate Polarized Partial Charges. J. Chem.
Theory Comput..

[26] Frisch, M. J. ; Gaussian 09. Revision E.01; Gaussian Inc.: Wallingford CT, 2009.

[27] Werner H.-J., Knowles P. J., Knizia G., Manby F. R., Schütz M. (2012). Molpro: a
general-purpose quantum chemistry program package. Wiley Interdiscip. Rev.: Comput. Mol. Sci..

