# Supplementary material for: Exploring the Mechanism of Ammonium Dinitramide Synthesis through DFT Calculations
Source: ACS Omega. 2025 Aug 30;10(36):41948–57. doi: 10.1021/acsomega.5c06735 (PMC12444511; doi:10.1021/acsomega.5c06735)
Supplement: Supplementary file 1 [file ao5c06735_si_001.pdf]

Supporting Information: Exploring the Mechanism of Ammonium Dinitramide (ADN) Synthesis Through DFT Calculations

Letícia M. S. V. Queiroz, Gabriel F. S. Fernandes, Josiane R. C. Silva, Francisco B.C. Machado, and Luiz F.A. Ferrão*

Department of Chemistry, Aeronautics Institute of Technology (ITA), 12228-900, São José dos Campos, SP, Brasil.

E-mail: ferrao@ita.br

Contents

1 Gas phase **3**

1.1 Stationary structures for calculations in gas phase 3

1.2 IRC calculations in gas phase 4

1.3 Potential surface scan for calculations in gas phase 6

1.4 Stationary states cartesian coordinates for calculations in gas phase 9

1.5 Frequency tables for calculations in gas phase 27

1.6 Table of energies corrected with triple-zeta basis set for calculations in gas phase 34

2 Implicit Solvent (PCM) **35**

2.1 Stationary structures for calculations with PCM 37

2.2 IRC calculations with PCM 38

2.3 Potential surface scan for calculations with PCM 39

2.4 Stationary states cartesian coordinates for calculations with PCM 42

2.5 Frequency tables for calculations with PCM 57

2.6 Table of energies corrected with triple-zeta basis set for calculations with water 65

3 Explicit Solvent: Water + PCM **66**

3.1 Results and Discussion 66

3.2 Stationary structures for calculations with explicit solvent 71

3.3 Stationary states cartesian coordinates for calculations with explicit solvent . 72

3.4 Frequency tables for calculations with explicit solvent 84

3.5 Table of energies for the mechanisms calculated with explicit solvent 88

1 Gas phase

1.1 Stationary structures for calculations in gas phase

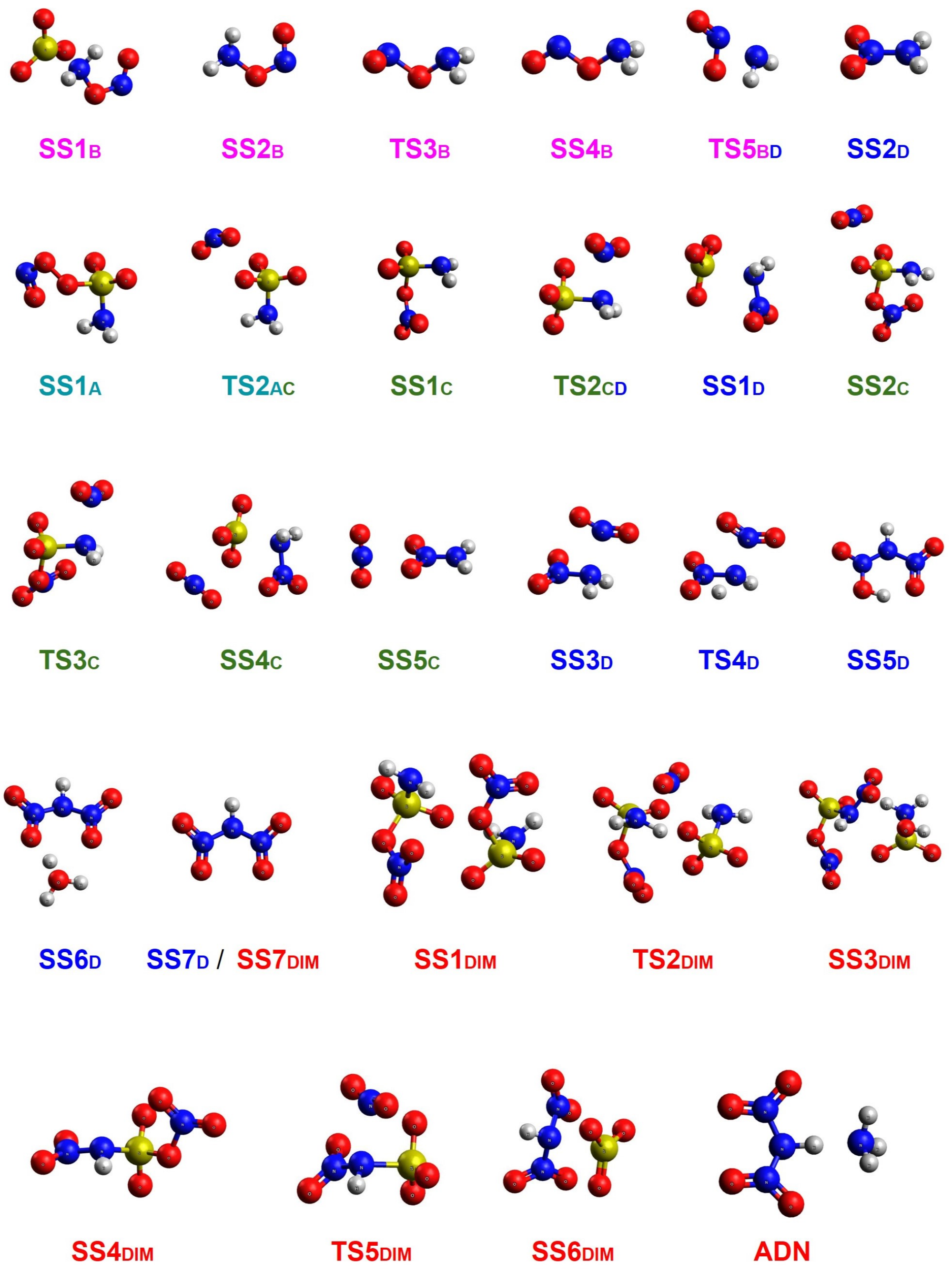

Figure S1: Stationary states for the gas-phase mechanism optimized with M06-2X/def2-SVP.

1.2 IRC calculations in gas phase

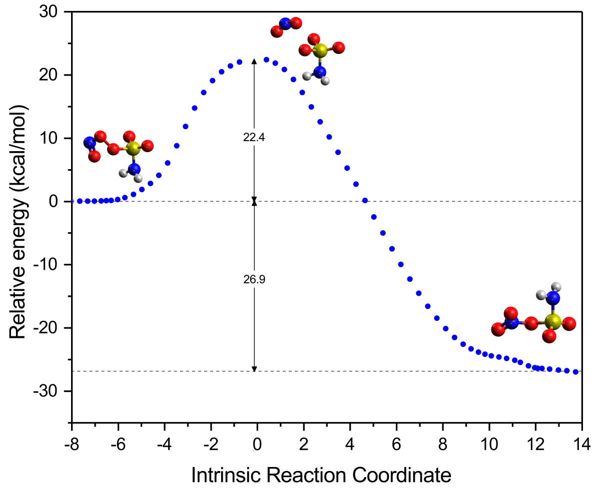

Figure S2: IRC for $TS2_{AC}$. Calculated with M06-2X/def2-SVP.

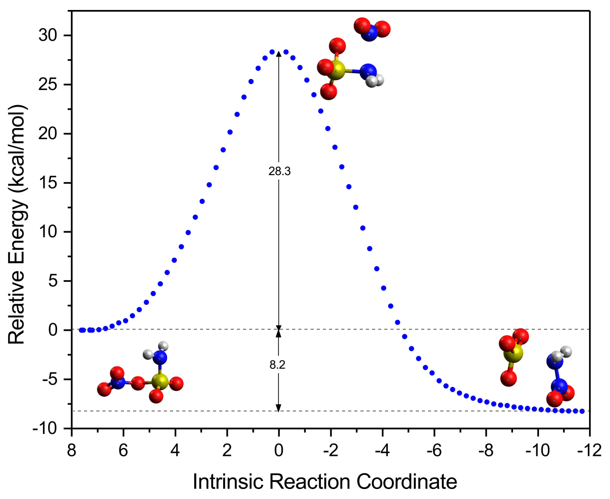

Figure S3: IRC for $TS2_{CD}$. Calculated with M06-2X/def2-SVP.

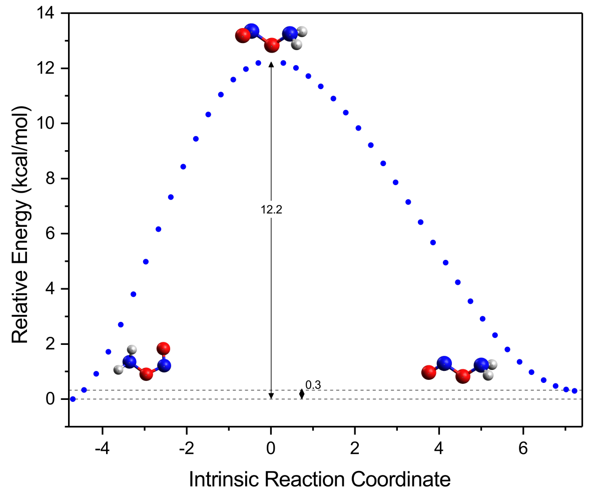

Figure S4: IRC for $TS3_{B}$. Calculated with M06-2X/def2-SVP.

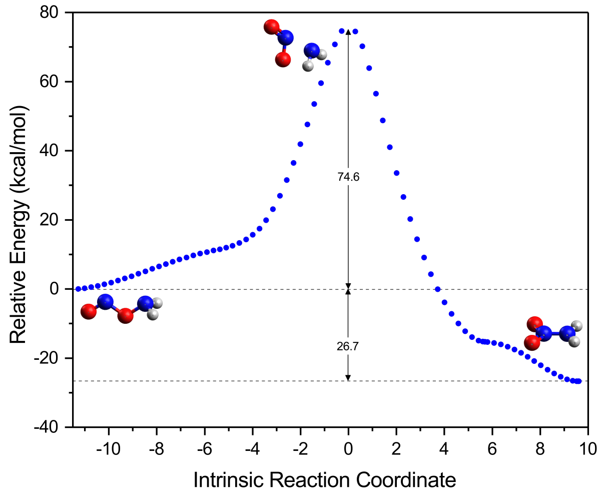

Figure S5: IRC for $TS5_{BD}$. Calculated with M06-2X/def2-SVP.

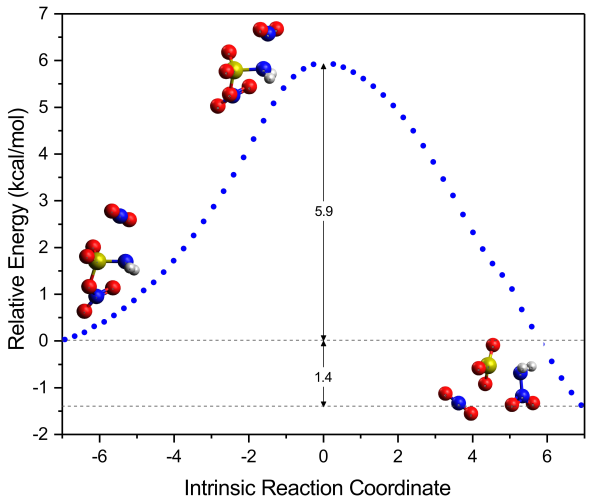

Figure S6: IRC for $TS3_C$. Calculated with M06-2X/def2-SVP.

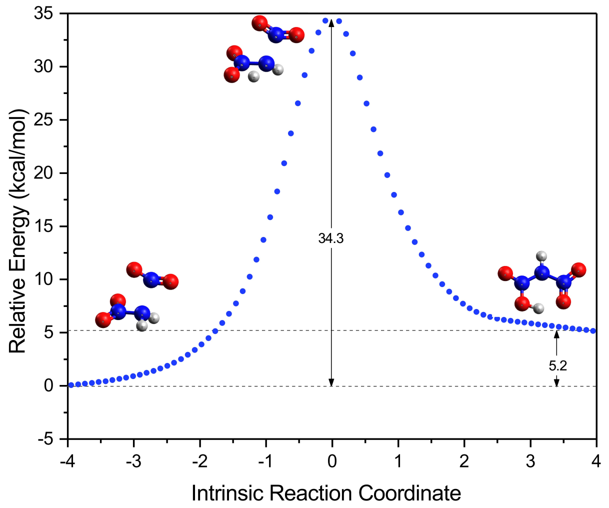

Figure S7: IRC for $TS4_D$. Calculated with M06-2X/def2-SVP.

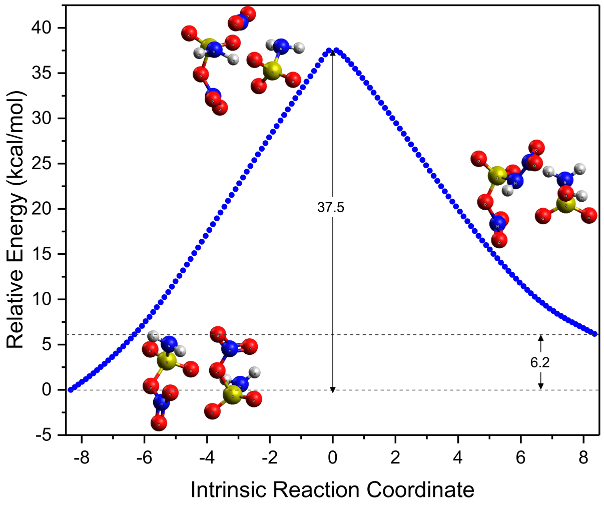

Figure S8: IRC for $TS2_{DIM}$. Calculated with M06-2X/def2-SVP.

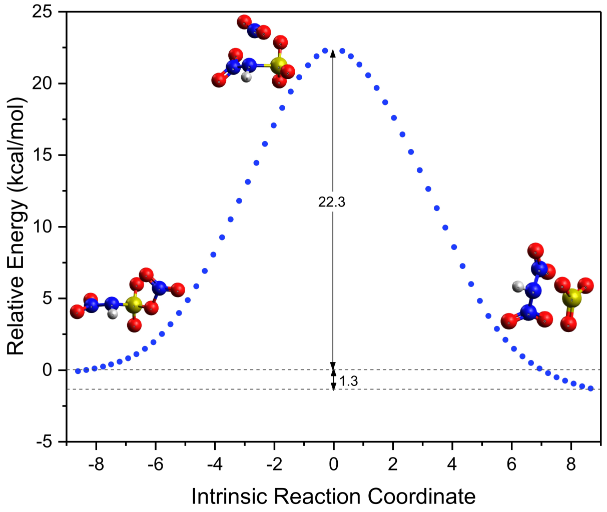

Figure S9: IRC for $TS5_{DIM}$. Calculated with M06-2X/def2-SVP.

1.3 Potential surface scan for calculations in gas phase

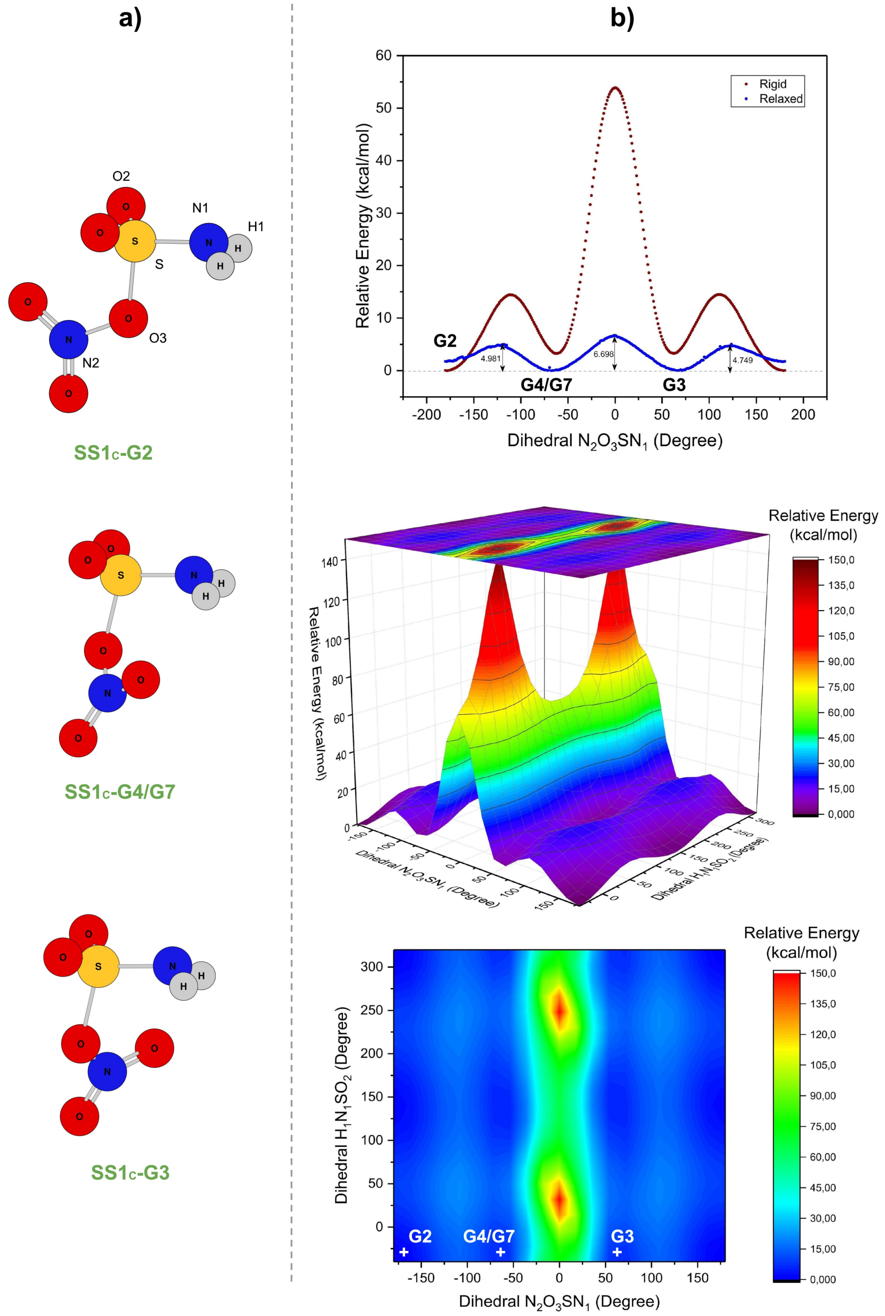

Figure S10: **a)** $SS1_C$ isomers obtained from G2, G3, G4 and G7. **b)** Potential energy surfaces connecting the 3 isomers. The first is a 1D PES (or PEC- Potential Energy Curve) scanning only one variable, the dihedral $N_2O_3SN_1$, responsible for the differentiation between the isomers. The second PES is a rigid 3D PES originated from the scan of two dihedrals (Dihedral $H_1N_1SO_2$ and Dihedral $N_2O_3SN_1$), showing the existence of other isomers from the rotation of this 2 dihedrals . The third image is the projection of the 3D PES, providing a 2D visualization of the surface. M06-2X/def2-SVP.

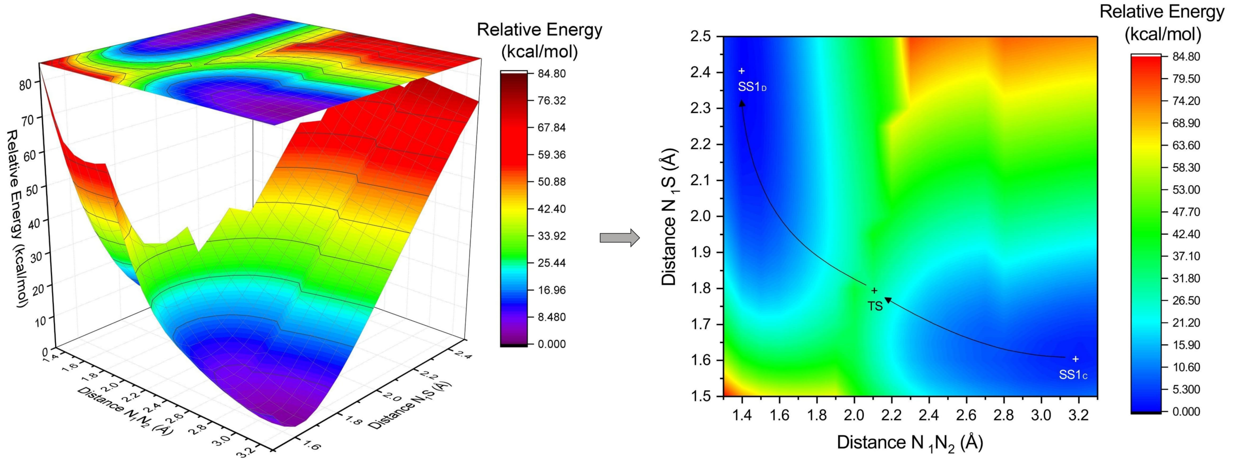

Figure S11: Rigid 3D PES between $SS1_C$ and $SS1_D$. M06-2X/def2-SVP.

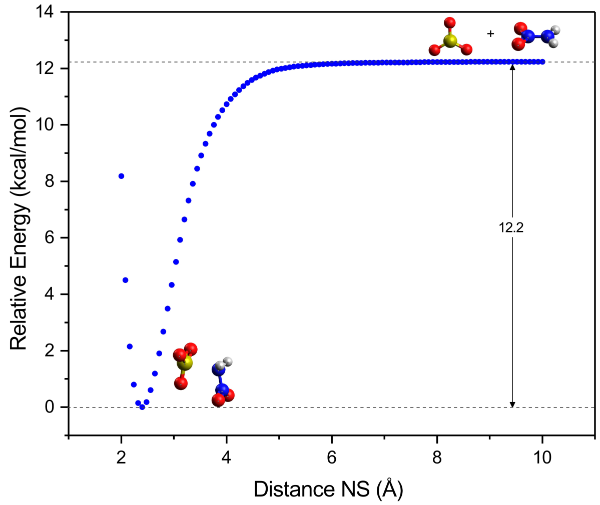

Figure S12: Rigid PEC representing the $SS1_D$ dissociation. M06-2X/def2-SVP.

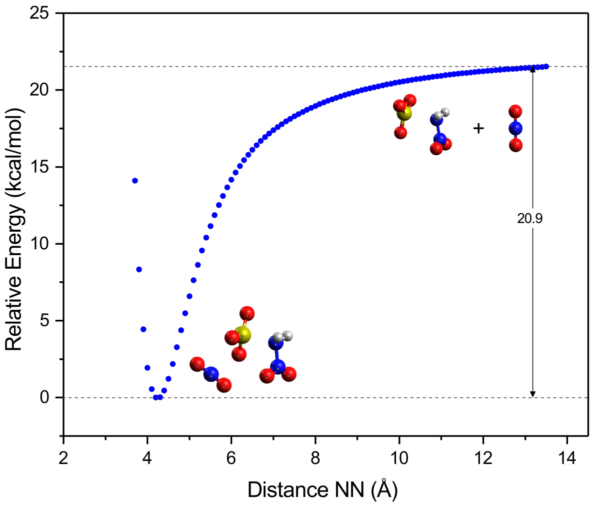

Figure S13: Rigid PEC representing the $SS4_C$ dissociation. M06-2X/def2-SVP.

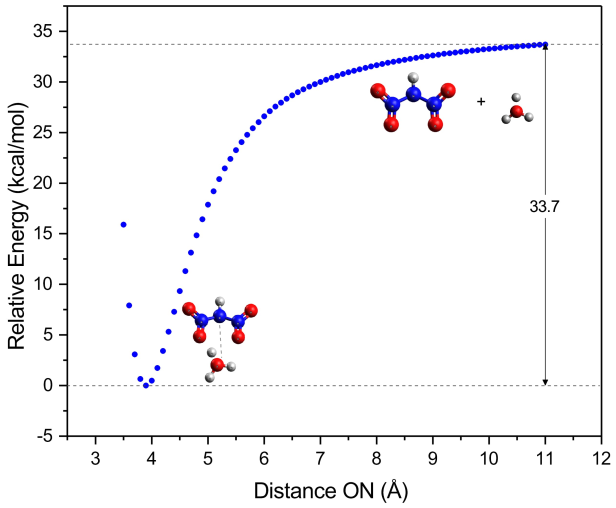

Figure S14: Rigid PEC representing the $SS6_D$ dissociation. M06-2X/def2-SVP.

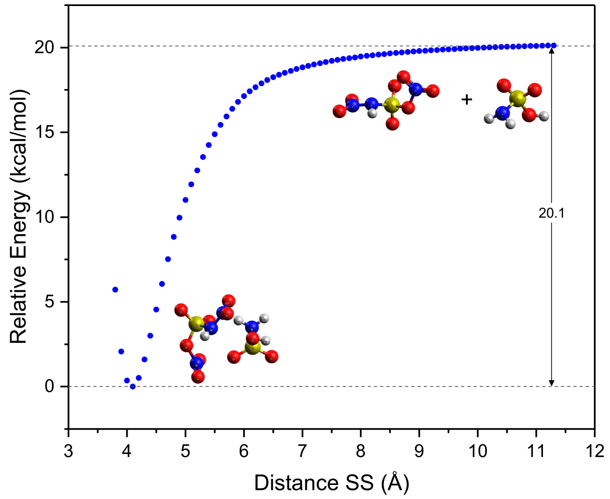

Figure S15: Rigid PEC representing the $SS3_{DIM}$ dissociation. M06-2X/def2-SVP.

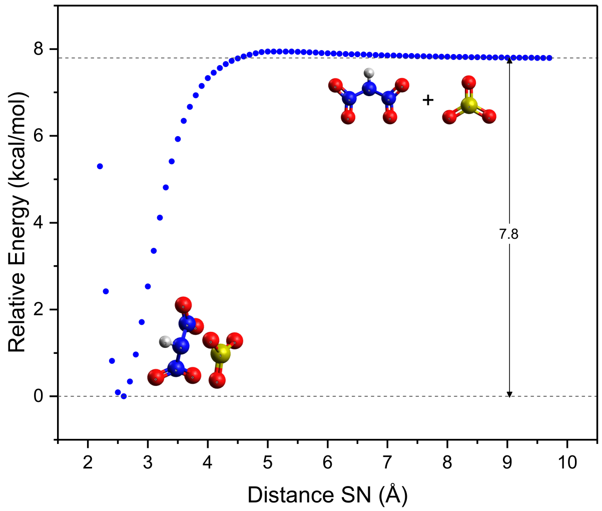

Figure S16: Rigid PEC representing the $SS6_{DIM}$ dissociation. M06-2X/def2-SVP.

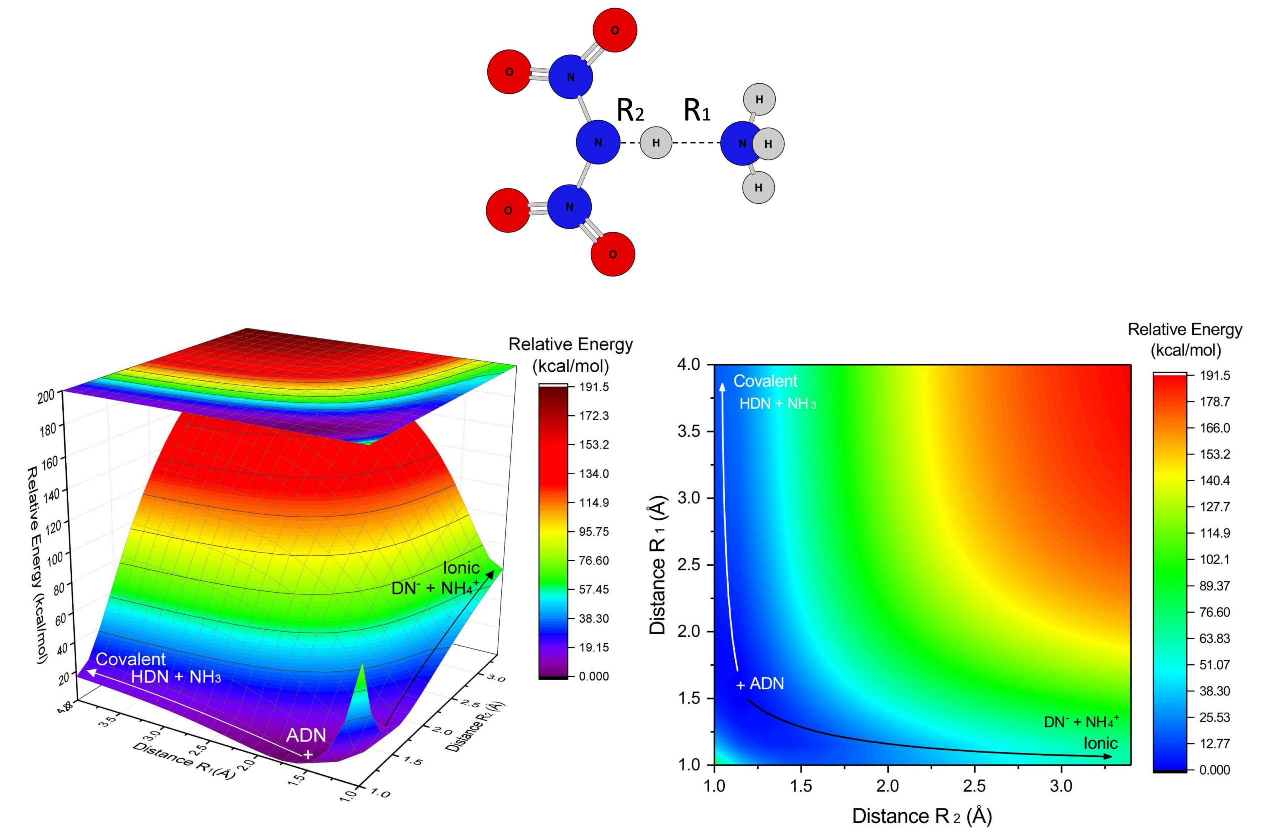

Figure S17: Rigid PES representing the formation of ADN by approximating thh NH_3 to HDN. M06-2X/def2-SVP.

1.4 Stationary states cartesian coordinates for calculations in gas phase

3

ss0 no2+

N 0.000000 0.000000 0.000000

O 0.000000 0.000000 1.108624

O 0.000000 0.000000 -1.108624

7

ss0 h2nso3-

O -0.629089 1.245966 -0.610555

O -0.630219 -1.244454 -0.612552

N 1.536899 -0.000375 -0.356102

S -0.163022 0.000039 0.009256

O -0.236884 -0.001131 1.483146

H 1.909581 -0.816337 0.132076

H 1.910010 0.815294 0.132238

10

ss1 from G1 optimized

O 2.107884 -0.795689 -0.227602

O 0.362974 -0.041562 1.459569

N 1.125889 1.468190 -0.382035

S 0.942791 -0.055540 0.149267

O -0.284055 -0.616975 -0.888174

H 0.285231 2.036775 -0.296945

H 1.614765 1.536543 -1.271481

N -2.278108 -0.082592 0.157959

O -1.398312 -1.089884 -0.201060

O -1.903381 0.996127 -0.049147

10

ss1 from G2 optimized

O 1.017750 -0.809015 -1.267553

O 1.013425 -0.801579 1.273016

N 1.994777 1.090957 -0.001426

S 0.960158 -0.151246 0.000707

O -0.470293 0.783168 -0.003949

H 2.029243 1.627932 0.861528

H 2.033009 1.621668 -0.868089

N -1.694265 0.079872 -0.000938

O -1.625550 -1.105342 -0.000195

O -2.626376 0.804584 0.000157

10

ss1 from G3 optimized

O 0.491528 -0.768904 -0.360049

O -0.843532 0.284457 1.487438

N -1.209410 1.205596 -0.794060

S -1.015619 -0.109536 0.124562

O -1.894031 -1.133582 -0.341401

H -0.569021 1.967262 -0.577536

H -1.384953 1.016430 -1.777725

N 1.647645 -0.037759 -0.075535

O 2.624377 -0.697762 -0.065486

O 1.513688 1.140046 0.085676

10

ss1 from G4 optimized

O 0.835075 0.272999 1.489904

O -0.490664 -0.764265 -0.372112

N 1.215359 1.211225 -0.782185

S 1.014601 -0.110755 0.125023

O 1.894546 -1.132114 -0.343956

H 1.398652 1.029316 -1.765791

H 0.573190 1.971234 -0.565064

N -1.647114 -0.036706 -0.078383

O -2.622179 -0.698975 -0.063415

O -1.514673 1.141092 0.083888

10

ss1 from G5 optimized

O 0.441095 -0.944349 -1.057929

O 1.799078 1.075359 -0.567979

N -0.498177 0.722379 0.807981

S 1.205282 -0.157180 -0.126430

O 1.687303 -0.771981 1.083613

H -0.250392 1.609582 1.255133

H -0.881191 0.061749 1.499433

N -2.361725 0.001894 -0.422005

O -1.469850 1.047035 -0.131181

O -2.224328 -0.934359 0.244287

10

ss1 from G6 optimized

O 1.741540 1.241413 -0.420324

O 0.655878 -0.002923 1.435157

N -0.744942 -0.000916 -1.045815

S 1.320400 0.000050 0.164253

O 1.745264 -1.238195 -0.424235

H -0.778833 -0.862054 -1.594276

H -0.778237 0.859589 -1.595311

N -1.809117 0.000015 -0.110260

O -2.176356 1.079957 0.245254

O -2.177690 -1.079256 0.245904

10

ss1 from G7 optimized

O 0.841706 0.283000 1.487774

O -0.491332 -0.768036 -0.362277

N 1.210661 1.206238 -0.792463

S 1.015412 -0.109681 0.124726

O 1.894197 -1.133452 -0.341154

H 1.388604 1.017703 -1.775845

H 0.569383 1.967368 -0.577005

N -1.647552 -0.037599 -0.076059

O -1.513892 1.140176 0.085552

O -2.623972 -0.698021 -0.065285

10

ss1 from G8 optimized

O 1.742450 1.240801 -0.421001

O 0.655842 -0.001753 1.435188

N -0.744954 -0.000231 -1.045842

S 1.320419 0.000010 0.164261

O 1.744431 -1.238908 -0.423563

H -0.778607 -0.861103 -1.594760

H -0.778476 0.860567 -1.594890

N -1.809136 -0.000019 -0.110268

O -2.177190 -1.079560 0.245671

O -2.176907 1.079686 0.245485

10

ts2-ac

O -1.848519 -1.157527 -0.398423

O -0.061837 0.429030 -1.165626

N -1.888294 1.207452 0.369165

S -0.937592 -0.076090 -0.105190

O -0.052573 -0.360302 1.122675

H -1.329222 1.987532 0.707619

H -2.578246 0.907400 1.055370

N 2.229170 -0.126071 -0.055178

O 1.665384 -1.071428 0.203081

O 2.362896 1.004331 -0.046438

6

ss2-b

N -1.238771 0.463169 -0.128515

H -2.160986 0.136055 0.158081

H -0.909263 1.124205 0.582186

N 0.859197 -0.520700 0.002041

O -0.473192 -0.713166 0.032198

O 1.189100 0.605973 -0.014067

6

ts3-b

N 1.358167 0.388671 -0.189598

H 1.663539 0.969285 0.591390

H 2.164393 -0.145082 -0.514390

N -0.794516 -0.363673 -0.384456

O -1.448467 0.424608 0.135391

O 0.476781 -0.549507 0.357282

6

ss4-b

N -1.583208 -0.232439 -0.000021

H -2.083918 0.113904 0.818938

H -2.083985 0.114141 -0.818838

N 0.672929 -0.454459 -0.000022

O 1.690375 0.105895 0.000006

O -0.372894 0.466636 0.000020

6

ts5-bd

N 1.297843 -0.613296 -0.066508

H 1.691908 -0.089979 -0.863027

H 1.801231 -0.263922 0.759147

N -0.425394 -0.027090 0.372015

O -1.393571 -0.408182 -0.173156

O 0.193536 1.012756 -0.081178

13

ss2-c

O 0.647237 -0.269792 0.568501

O -1.349686 0.137991 -0.844831

N -0.753592 1.680645 1.205974

S -0.118224 0.837087 0.003895

O 0.489944 1.701522 -0.958612

H -1.184780 2.567126 0.943303

H -1.191482 1.145051 1.956012

N -2.198184 -0.838516 -0.159319

O -2.132633 -0.823300 1.024949

O -2.819298 -1.467342 -0.918719

N 2.839727 -0.738913 -0.044153

O 2.849162 -1.609749 0.642055

O 2.946799 0.102161 -0.760738

13

ts3-c

O 0.103880 0.017813 -1.294247

O -1.564371 0.736191 0.401736

N 0.794194 -0.148084 1.082045

S -0.062994 0.800030 -0.102626

O 0.419399 2.125895 0.080382

H 0.967430 0.390517 1.941487

H 0.306070 -1.031238 1.285779

N -2.392846 -0.576316 0.042913

O -1.779623 -1.548617 0.260964

O -3.444027 -0.280941 -0.313706

N 2.508704 -0.631777 -0.011646

O 2.398502 -1.743239 -0.177641

O 3.036743 0.359583 -0.129793

13

ss4-c

O -0.315282 0.391784 -1.181175

O -0.785738 0.781428 1.160735

N 1.834108 0.388676 0.484066

S -0.077206 1.231571 -0.021222

O 0.390233 2.569431 -0.147774

H 2.609391 0.876842 0.016494

H 1.916783 0.407700 1.509385

N -2.212672 -0.994131 -0.077204

O -1.542914 -1.834315 0.188914

O -2.944275 -0.205524 -0.347861

N 1.811106 -0.994757 0.047967

O 1.044877 -1.673437 0.667093

O 2.488266 -1.252891 -0.886199

9

ss5-c

N 2.446740 -0.004621 0.001762

H 2.909610 0.885780 0.156823

H 2.901302 -0.898802 -0.155720

N -1.676524 -0.001002 0.001727

O -1.751527 0.196409 -1.087038

O -1.743139 -0.197377 1.091226

N 1.134468 0.001318 -0.001680

O 0.547361 -1.053249 -0.189459

O 0.554342 1.059612 0.183550

4

ss2-d so3

O -1.192966 -0.794353 0.000001

O 1.284956 -0.635068 0.000001

S 0.000021 -0.000264 -0.000001

O -0.092032 1.429949 0.000001

6

ss2-d h2nno2

N 1.227985 -0.001239 -0.088611

H 1.615958 0.869617 0.259530

H 1.614846 -0.871593 0.262046

N -0.141946 0.000061 -0.003748

O -0.675917 1.079463 0.007916

O -0.678217 -1.078186 0.007701

9

ss3-d

N 0.458001 -0.932723 0.611091

H 0.513127 -0.806938 1.627020

H 0.750601 -1.879988 0.352571

N -1.753424 0.156896 -0.135532

O -2.336615 -0.789741 -0.127396

O -1.283842 1.161328 -0.178888

N 1.390379 -0.019200 0.031700

O 2.080472 -0.442138 -0.839454

O 1.298933 1.102066 0.454437

9

ts4-d

N -0.065427 -0.830734 -0.292317

H -0.792643 -1.332530 0.682643

N -1.250881 -0.009326 -0.162434

O -1.817352 -0.550442 0.807527

O -1.530524 0.919569 -0.798645

N 1.383660 0.106900 0.095096

O 2.295935 -0.449768 -0.329023

O 1.085728 1.039070 0.706051

H 0.050889 -1.202785 -1.252334

9

ss5-d: HDN-H

N -0.457581 -0.942048 0.597119

H -0.751700 -1.885342 0.326065

N 1.753978 0.158916 -0.135860

O 2.341934 -0.784691 -0.120879

O 1.279504 1.160754 -0.185311

N -1.390893 -0.019693 0.033643

O -1.295318 1.095978 0.470195

O -2.085747 -0.430071 -0.839664

H -0.509809 -0.830643 1.614894

3

h2o

O 0.000000 0.119260 0.000000

H 0.755892 -0.477044 0.000000

H -0.755892 -0.477034 0.000000

12

ss6-d: HDN-H3O+

N -1.151137 0.184822 0.345064

H -2.149903 0.360492 0.190659

N -0.782962 -1.150019 0.005965

O -1.708123 -1.870861 -0.128624

O 0.390441 -1.395798 -0.046648

N -0.373152 1.293522 0.009607

O 0.842399 1.186802 -0.015361

O -0.980714 2.290356 -0.157225

H 1.862655 0.157301 0.000131

O 2.750199 -0.391180 -0.063020

H 2.590569 -1.337739 0.116429

H 3.493819 -0.032877 0.455351

8

ss7-d: HDN

N 0.000053 -0.588686 0.417995

H 0.000087 -1.581821 0.181358

N 1.235304 -0.001840 0.014768

O 2.085088 -0.823992 -0.184355

O 1.310899 1.182215 -0.022965

N -1.235305 -0.001939 0.014889

O -1.311240 1.182116 -0.022024

O -2.084803 -0.824205 -0.185021

4

ss7-d: H3O+

H -0.610370 -0.725779 0.189376

O 0.000049 0.000016 -0.071040

H 0.933568 -0.165596 0.189507

H -0.323591 0.891245 0.189438

20

ss1-dim

O 0.756220 0.912505 0.996114

O 2.929565 -0.030990 0.156124

N 1.311070 1.317105 -1.381243

S 1.853517 1.266366 0.132667

O 2.738755 2.359441 0.372047

H 2.010112 1.566162 -2.076688

H 0.644120 0.577686 -1.619602

N 2.380012 -1.352934 0.127458

O 2.653857 -1.949121 -0.851938

O 1.782238 -1.674018 1.098979

H -0.299514 -0.717025 1.620186

O -1.832309 0.564392 -0.538405

O -0.179395 -1.097958 -0.985878

O -3.343842 2.092411 -0.390144

N -2.992515 1.065022 0.066665

N -1.132469 -1.222054 1.302522

S -1.426450 -1.074166 -0.261028

O -3.453579 0.415665 0.956963

H -1.964480 -1.198908 1.888683

O -2.574759 -1.836464 -0.634689

20

ts2-dim

O -0.645451 0.579001 -1.489827

O -2.540670 -0.309007 -0.086090

N -0.605523 1.158809 0.885469

S -1.542722 0.961291 -0.424561

O -2.481278 2.033813 -0.583087

H -1.140894 1.431214 1.712689

H 0.315224 0.344353 0.985366

N -2.026609 -1.556309 0.510184

O -1.547627 -1.417281 1.579259

O -2.273486 -2.491954 -0.144777

H 1.253622 -0.309920 -2.092260

O 1.504873 -0.032954 0.885669

O 0.479390 -1.910116 -0.266484

O 1.633608 2.498678 1.491535

N 1.360722 2.175470 0.444432

N 2.096314 -0.392444 -1.521876

S 1.743777 -1.212090 -0.106701

O 1.395279 2.193823 -0.678052

H 2.849013 -0.878151 -2.006159

O 2.941840 -1.928178 0.250989

20

ss3-dim

O 0.972588 -0.004894 1.542413

O 2.233342 -0.761778 -0.486031

N 0.682252 1.087834 -0.747990

S 1.749022 0.451253 0.425171

O 2.870785 1.322733 0.585584

H 1.004117 1.061174 -1.715947

H -3.061662 0.885560 -1.123914

N 1.441267 -2.145781 -0.463882

O 1.211471 -2.455204 -1.556162

O 1.341387 -2.586253 0.601789

H -0.961726 0.174445 1.809769

O -2.158500 0.714229 -0.814819

O -0.999764 -1.366782 -0.294538

O -0.330505 2.913285 -1.406173

N -0.009292 2.258332 -0.460054

N -1.923683 -0.116947 1.618419

S -2.171776 -0.627643 0.103293

O -0.244611 2.459702 0.701478

H -2.659583 0.472046 1.997660

O -3.507553 -1.154419 -0.004597

12

ss4-dim

O 0.271142 0.902673 1.394472

O 1.348802 0.238233 -0.775016

N -0.894429 -0.640690 -0.289516

S 0.003053 0.783408 0.000128

O -0.474123 1.843628 -0.826952

H -0.732108 -1.081287 -1.196886

N 2.306949 -0.591060 -0.007333

O 1.820526 -1.381064 0.715322

O 3.409897 -0.329740 -0.300226

O -2.941229 -1.372926 -0.589938

N -2.269641 -0.573341 -0.008210

O -2.599626 0.246994 0.798620

8

ss4-dim fragment h2nso3h

H 1.740697 -0.784865 -0.815392

H -1.982226 -0.225005 -0.795400

O 0.811634 -0.685194 -1.078521

O -0.071268 1.499149 -0.296245

N -1.452027 -0.528910 0.017304

S 0.041302 0.124247 0.100774

H -1.479018 -1.529426 0.196254

O 0.662622 -0.282241 1.334894

12

ss5-dim

O 0.306514 -2.195566 -0.596567

O 1.197558 0.055561 -1.142638

N -0.504926 -0.046527 0.726975

S 0.901907 -0.974583 -0.132880

O 1.788001 -0.975538 1.010070

H -0.567039 -0.305149 1.716941

N 0.480641 1.809668 0.043878

O -0.249957 2.199786 -0.720587

O 1.316767 1.905183 0.797852

O -2.683376 -0.418535 0.910800

N -1.778696 -0.171130 0.161049

O -1.830833 0.023408 -1.023202

12

ss6-dim

O -1.617462 -1.569027 0.225528

O -1.456705 0.711149 1.198898

N 0.729204 0.054137 -0.607423

S -1.738003 -0.147635 0.086188

O -2.200243 0.412521 -1.149295

H 0.876297 0.112879 -1.620543

N 1.137070 1.335611 -0.029827

O 1.642694 1.321814 1.038801

O 0.851795 2.244663 -0.744933

O 1.955499 -1.763822 -0.953401

N 1.420163 -1.117577 -0.102004

O 1.315257 -1.314287 1.061441

8

ss7-dim: HDN

N 0.000068 -0.588653 0.417839

H 0.000069 -1.581809 0.181257

N -1.235377 -0.001902 0.014808

O -1.311333 1.182145 -0.022219

O -2.084852 -0.824244 -0.184772

O 2.085108 -0.824023 -0.184415

N 1.235329 -0.001867 0.014773

O 1.311051 1.182217 -0.022744

12

ADN

N 0.055554 -0.000191 0.427114

H 1.129141 -0.001891 0.259267

N -0.520167 1.217666 0.039584

O 0.299077 2.099439 -0.049939

O -1.698810 1.301410 -0.115057

N -0.524169 -1.216167 0.039685

O -1.703202 -1.296235 -0.113939

O 0.292272 -2.100450 -0.050714

N 2.719956 -0.003933 -0.090752

H 2.963204 -0.830815 -0.635849

H 3.309731 -0.004227 0.740861

H 2.965018 0.821987 -0.636495

1.5 Frequency tables for calculations in gas phase

Table S1: Frequencies for all stationary states and transition states for calculations in gas phase.

SS1 from G1 (SS1a)				
85.5543	129.6130	236.6120	254.9858	321.4380
348.3718	434.6653	448.8335	472.5277	524.2093
561.4624	614.8270	695.8508	728.3146	915.3655
929.4717	1026.6369	1080.7680	1241.6847	1491.0349
1543.7606	1887.2265	3549.7529	3676.9842	

SS1 from G2 (SS1c)				
63.6613	106.3758	177.4078	289.0105	301.9774
391.8277	430.4617	468.4611	495.5450	512.6198
611.8330	738.7519	812.2301	828.5670	912.7236
946.8903	1059.9501	1242.0480	1462.6417	1492.0873
1540.3320	1923.0665	3562.2342	3693.6724	

SS1 from G3 (SS1c)				
76.1005	135.3187	215.7883	309.7755	324.9521
388.8046	420.4274	478.6797	527.0746	539.6238
626.8513	747.3681	789.8535	853.5331	910.3939
974.5353	1080.8699	1250.1216	1458.2491	1504.3193
1543.0675	1910.0348	3553.9742	3686.3623	

SS1 from G4 (SS1c)				
75.6232	134.5742	215.3980	309.5425	324.8839
388.9905	421.5106	478.7066	527.5942	539.9729
627.1831	746.8482	788.3016	853.6855	910.4893
974.6847	1081.1877	1250.3957	1458.2983	1504.7081
1543.3117	1910.3295	3554.3499	3686.7648	

SS1 from G5 (SS1b)				
26.8713	88.2002	127.4890	184.4154	221.2034
310.2420	387.1024	465.5629	499.0804	533.5205
550.8480	625.5372	710.4756	906.3429	1075.6555
1104.5776	1199.0900	1344.0607	1418.0267	1426.9305
1550.4023	1891.8077	3419.2529	3548.4551	

SS1 from G6 (SS1d)				
35.9646	71.5291	85.3665	162.1973	174.4133
224.9298	487.7888	495.5665	529.8562	536.1184
658.1244	719.9828	785.1718	935.8789	1038.6652
1107.7383	1242.0980	1431.2110	1434.7156	1485.9139
1572.9047	1885.1472	3502.7182	3631.7642	

SS1 from G7 (SS1c)				
75.9831	135.2112	215.6126	309.6658	324.9386
388.7517	420.4656	478.6612	527.1671	539.1663
626.5790	747.2501	789.5765	853.5511	910.4067
974.5492	1080.8272	1250.1448	1458.2624	1504.3388
1542.9827	1910.1230	3554.3304	3686.6160	

SS1 from G8 (SS1d)				
36.4548	71.7241	85.4282	162.2485	174.5008
224.9632	487.8425	495.6379	529.8616	536.1388
658.1614	719.9506	785.2025	935.8809	1038.6502
1107.5716	1242.1051	1430.9993	1434.5139	1485.8158
1572.9144	1885.0041	3502.5575	3631.6031	

TS2ac				
-274.7597	53.2526	104.6345	170.6323	224.7010
253.4228	327.5867	423.1955	458.6165	536.2719
542.2195	559.7328	595.1213	689.5871	859.6664
962.0503	1077.3377	1177.0999	1391.6828	1459.9204
1551.9121	2354.5576	3544.8197	3662.9920	

SS2b				
196.2749	414.0031	450.1591	840.8332	901.6422
1048.1672	1157.6008	1406.2090	1600.2369	1822.0886
3463.9979	3597.6477			

TS3b				
-254.9813	300.6759	375.7114	574.5791	815.5591
946.7548	1174.3135	1334.6210	1646.6918	1949.4584
3499.6770	3596.2266			

SS4b				
203.2990	272.8093	431.6834	652.4904	862.9632
1084.4717	1197.4040	1362.0524	1663.1142	1909.8739
3490.2153	3582.7344			

TS5bd				
-1241.0952	219.2406	328.0702	471.9478	782.8214
997.8925	1015.5285	1178.4898	1495.9155	1797.9485
3406.3415	3533.6030			

SS2c				
40.1587	48.9214	61.7062	79.2912	121.1039
185.9935	194.3587	217.8454	302.5253	330.1327
381.2116	433.9340	479.9697	519.1694	536.1799
546.0498	619.5511	646.0581	694.5559	781.3285
821.6493	890.3864	988.5353	1048.4151	1222.3684
1429.1680	1454.8213	1529.9643	1559.5651	1982.4510
2611.2465	3520.2412	3650.3350		

TS3c				
-179.2840	30.0197	51.8040	61.3532	80.9285
122.1917	176.4951	254.9512	290.9574	308.0519
358.0733	457.2769	529.9545	555.0884	594.5923
620.6818	659.0613	698.4560	719.6677	776.0126
807.2795	926.1015	1017.1758	1059.6581	1233.0394
1464.9465	1488.0773	1503.2800	1527.6279	2100.3946
2373.5215	3409.9000	3520.8427		

SS4c				
53.9291	63.6920	95.5754	99.7176	110.6097
130.9575	171.5167	181.2408	232.6866	237.5133
288.5467	501.6669	519.0292	540.9372	555.0514
586.1468	613.4727	696.7196	753.8911	798.4044
944.4543	1095.1713	1136.0972	1226.4278	1352.5622
1463.4978	1477.0887	1557.7156	1570.8898	1916.3390
2629.2097	3425.1771	3539.6426		

SS5c				
90.9811	116.2830	120.6636	196.8101	212.4856
255.9657	548.3585	585.1089	592.5002	608.0961
778.7700	848.2424	1162.0418	1183.4723	1539.8161
1562.8739	1608.8946	1716.2476	2616.7945	3560.9457
3734.7827				

SS2d				
463.1954	593.2743	598.1741	747.3207	829.5399
1088.6536	1244.4324	1506.9325	1586.4337	1834.9505
3574.1520	3722.5823			

SS3d				
37.2610	60.9646	140.3088	171.0370	204.8789
491.8591	506.5057	605.1227	642.5037	712.2287
781.3662	956.1968	1095.1241	1268.2608	1479.5069
1546.4612	1591.9006	1875.4728	2598.8927	3462.9800
3567.8022				

TS4d				
-1749.9574	48.2530	109.8281	147.9266	260.6862
388.0486	594.9937	658.8602	755.7519	788.5033
855.1179	919.9383	1008.1688	1253.1027	1277.5655
1404.6544	1484.9549	1897.8653	2156.8613	2240.8544
3400.1423				

SS5d				
35.3205	62.1252	140.9164	170.6374	204.3152
491.4476	506.2929	604.8199	642.5255	712.3890
781.1336	956.7384	1094.9866	1267.9941	1479.9032
1546.2003	1591.2822	1875.5306	2598.3538	3463.1922
3567.9795				

SS6d				
57.0483	78.2464	109.2850	176.7805	245.0151
263.1579	318.7383	422.1455	460.7187	475.8182
505.4262	643.1538	785.3016	791.9547	816.5270
883.2509	988.4257	1012.1632	1078.5926	1356.4274
1437.0906	1513.1698	1561.5106	1630.2021	1848.6243
1912.4825	2529.3489	3518.4712	3719.8341	3811.2224

SS7d/SS7dim (HDN)				
50.4702	130.5442	257.3239	441.5933	472.5965
681.2470	788.2754	805.8936	836.9478	893.1080
983.9635	1072.9359	1406.9669	1425.2329	1499.8572
1862.6804	1906.2300	3582.1186		

SS1dim				
29.8303	37.1372	49.5692	70.2506	85.9531
102.8748	113.1399	128.9056	153.9235	171.2616
216.9271	222.8408	297.3622	321.9419	329.5371
357.7192	401.2244	406.3535	421.1437	466.3649
489.8370	501.7871	527.4450	530.9371	559.6326
593.7410	601.1503	658.0701	696.0491	740.4061
749.9236	796.9820	841.5901	873.4886	898.6096
911.7300	965.5882	991.0215	1093.4029	1109.7823
1225.5706	1251.0223	1451.1595	1464.2195	1472.8407
1488.1760	1545.5196	1549.8113	1913.2708	1939.4591
3475.9756	3495.0728	3649.8892	3655.4370	

TS2dim				
-891.7928	15.4865	54.6589	65.5250	88.2253
107.1827	119.4892	123.7370	138.6604	156.5305
178.8059	209.3931	215.4479	265.0246	273.8785
294.4199	322.8370	357.7982	394.8705	414.8903
464.7139	492.1997	512.5654	529.8971	545.4454
575.1011	583.2953	595.0232	643.4524	683.0598
694.2468	706.4704	788.7111	848.4213	866.8046
884.3369	961.8539	1029.5209	1106.9467	1121.5771
1217.0459	1244.3946	1415.2460	1442.7021	1461.1850
1477.5504	1532.2741	1542.6212	1601.2388	1988.9266
2395.2361	3525.6062	3558.9373	3646.2278	

SS3dim				
26.0529	58.8585	86.4184	94.7071	110.7852
113.8280	117.5793	131.5755	137.7893	148.0110
175.5959	207.5064	220.0289	255.8591	309.3010
360.2489	361.0939	375.3066	437.2506	454.7020
462.9250	490.8479	526.4315	536.7426	554.8898
560.7877	607.5092	621.0982	650.6868	684.0970
798.9506	809.5700	817.7739	842.6182	898.6984
925.8846	953.9702	1058.4586	1087.6267	1137.3246
1228.8397	1238.1402	1367.8369	1455.7097	1461.7948
1479.4244	1500.0996	1547.8497	1854.0453	2104.8356
3498.1388	3582.2049	3665.9307	3833.0444	

SS4dim				
41.9843	57.3623	63.4339	107.4122	170.4062
182.1233	275.6891	298.8436	385.7770	428.6909
462.5580	545.8786	573.5525	606.9652	683.4328
739.9398	797.2960	806.5889	839.4423	890.0579
905.8790	1031.6571	1243.5594	1370.7392	1459.3419
1477.4756	1503.7443	1863.5999	1993.0808	3568.9573

TS5dim				
-363.8218	67.7645	79.9322	141.7426	148.3459
188.6309	243.0690	291.0644	310.5916	328.3726
344.7739	511.4866	516.3669	544.9376	581.2250
631.7422	729.6008	767.6918	855.3059	912.2284
1028.1419	1079.8130	1293.7915	1343.1210	1426.3346
1458.1341	1488.4453	1834.7131	2385.1797	3513.7102

SS6dim				
38.4116	39.8404	57.5684	80.1673	98.2884
114.1892	144.1216	190.9352	263.9019	430.9894
464.0344	502.0248	526.3567	528.5807	691.0658
756.4726	796.5306	877.1066	883.2051	922.6121
1066.8208	1112.9786	1386.5888	1419.1112	1440.4587
1442.5349	1493.6186	1891.5319	1929.0874	3511.0416

ADN				
-15.6332	20.2771	60.7677	72.4339	122.2088
241.5799	298.6557	353.6489	414.0468	462.6911
488.8709	794.0884	805.8149	811.6617	868.2767
1004.2971	1070.1275	1120.1483	1256.0583	1416.2291
1496.0514	1580.2392	1624.3178	1633.8887	1832.7488
1865.1080	2469.5624	3502.2866	3632.5020	3634.1049

1.6 Table of energies corrected with triple-zeta basis set for calculations in gas phase

Table S2: Relative energies (kcal.mol^{-1}) for gas phase calculations. M06-2X/def2-TZVP//M06-2X/def2-SVP

Species	ΔE	ΔH(0K)	ΔH(298.15K)	ΔG(298.15K)
SS0	0.00	0.00	0.00	0.00
$SS1_A$	-128.46	-128.36	-128.83	-118.01
$TS2_{AC}$	-109.67	-109.88	-110.23	-100.14
$SS1_B$	-147.20	-146.51	-146.65	-137.50
$SS2_B$	-129.37	-130.36	-130.29	-132.80
$TS3_B$	-118.11	-120.07	-120.16	-122.24
$SS4_B$	-129.25	-130.49	-130.29	-132.80
$TS5_{BD}$	-55.38	-58.75	-58.67	-61.13
$SS1_C$	-157.01	-155.33	-156.02	-144.94
$TS2_{CD}$	-126.52	-125.77	-126.66	-115.04
$SS2_C$	-177.33	-175.44	-175.83	-156.52
$TS3_C$	-163.88	-162.21	-163.07	-142.96
$SS4_C$	-181.56	-178.76	-179.10	-159.58
$SS5_C$	-180.49	-179.66	-179.78	-172.72
$SS1_D$	-161.27	-159.68	-159.80	-150.97
$SS2_D$	-153.82	-153.53	-153.80	-155.52
$SS3_D$	-162.32	-161.27	-161.44	-155.04
$TS4_D$	-122.41	-124.42	-125.15	-117.21
$SS5_D$	-162.32	-161.55	-162.54	-153.76
$SS6_D$	-190.84	-187.40	-189.12	-170.31
$SS7_D$	-164.08	-161.94	-163.01	-154.70
$2SS1_C$	-314.02	-310.66	-312.04	-289.87
$SS1_{DIM}$	-323.20	-319.15	-320.12	-286.57
$TS2_{DIM}$	-280.61	-280.02	-281.08	-246.50
$SS3_{DIM}$	-326.66	-322.70	-323.49	-289.71
$SS4_{DIM}$	-315.73	-313.28	-314.07	-293.73
$TS5_{DIM}$	-297.44	-295.46	-296.49	-274.81
$SS6_{DIM}$	-319.66	-317.60	-317.83	-299.38
$SS7_{DIM}$	-316.15	-314.74	-315.43	-307.41

2 Implicit Solvent (PCM)

As explained in the main text, there are at least 20 synthesis routes of ADN, however the most economically and safety-wise advantageous is the nitration of sulfamate anion by the nitronium cation (${NO_2}^+$), obtained either from a sulfonitric mixture or from nitronium salts. In both routes the presence of water molecules is minimal, since the $H_2SO_4{-}HNO_3{-}H_2O$ ternary diagram demands low water content to displace the equilibrium to form ${NO_2}^+$ in sulphonitric mixtures. In the present paper we are considering the routes using nitronium, since we have collaborated with experimental groups exploring this route.

In this sense, the implementation of water as an implicit solvent was an approximation used to analyze the effect of a dielectric medium on the mechanism. In the Polarizable Continuum Model (PCM) the solute is placed within an electrostatic cavity encircled by a dielectric medium representing the solvent. The absence of water molecules enables our system to be adequately defined by not considering the direct interaction between the solute and the solvent by hydrogen bonds. The inclusion of water molecules into a system containing ${NO_2}^+$ results in a scenario where ${NO_2}^+$, can directly interact with the water molecule acting as a Bronsted base. Conversely, H_2O would act as a Bronsted acid, resulting in the formation of ${NO_3}^-$. This process is illustrated in equation 1:

$$ {NO_2}^+ + H_2O \longrightarrow 2H^+ + {NO_3}^- \tag{1} $$

In this sense, although water molecules may reduce energy barriers, particularly those associated with hydrogen abstraction processes, as suggested in the main text, the implementation of explicit molecules must be done with caution to avoid steering the mechanism toward unintended pathways.

In addition, since the real synthesis occurs in an ionic liquid, the initial objective of our manuscript was to assess the impact of a polarized field on the mechanism. Given the expectation that highly polar substances, such as nitric acid and sulfuric acid, possess a high

dielectric constant, just as water, the utilization of water as an implicit solvent should not be a problem. It is evident that the energy of the stationary states, as well as their relative energies, may be subject to variations in accordance with the selected solvent. Nevertheless, it is expected that the mechanism will remain predominantly unchanged.

Nevertheless, calculations using explicit water were performed and can be verified in Section 3.

2.1 Stationary structures for calculations with PCM

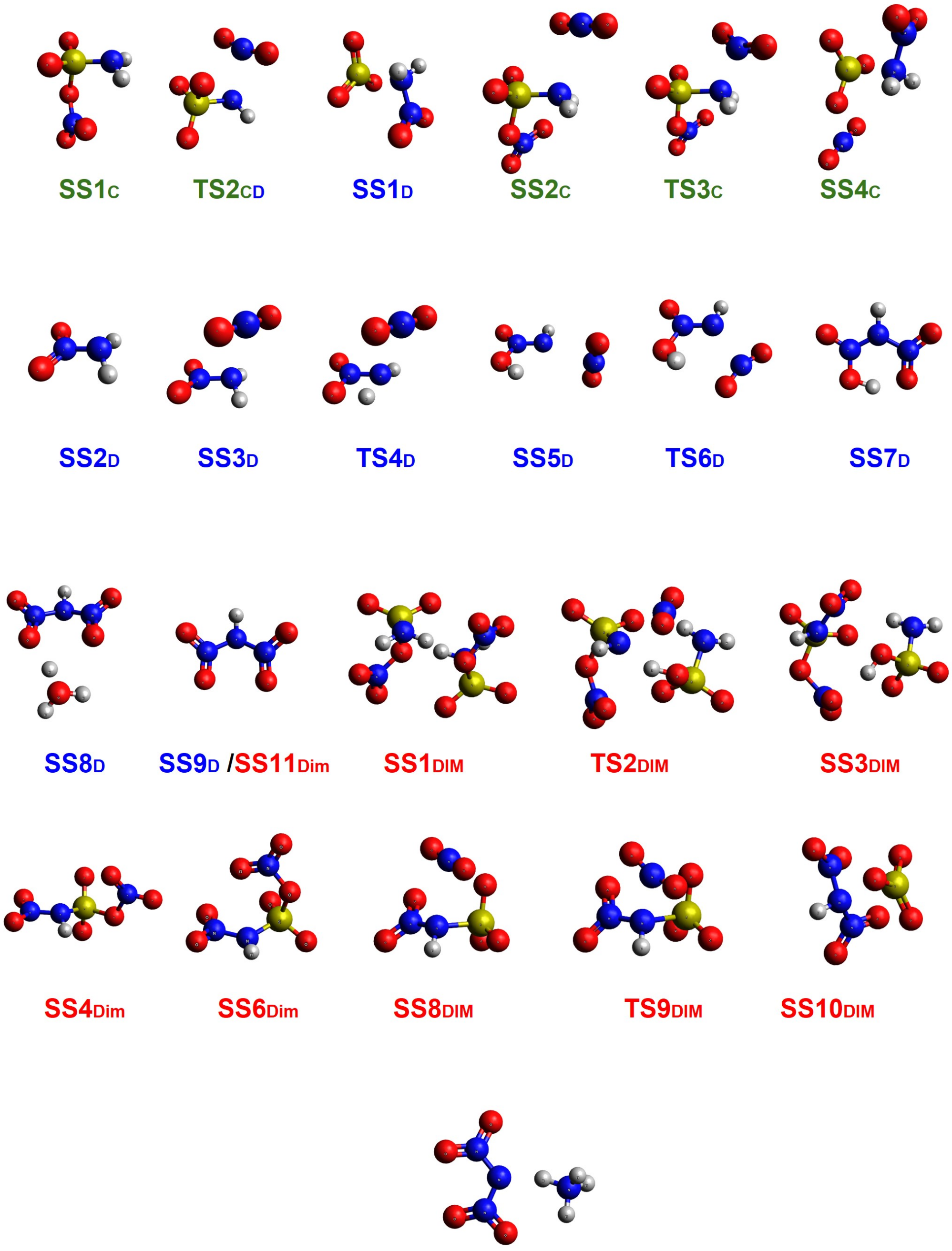

ADN

Figure S18: Stationary states for the mechanism under implicit solvent optimized with M06-2X/def2-SVP/PCM.

2.2 IRC calculations with PCM

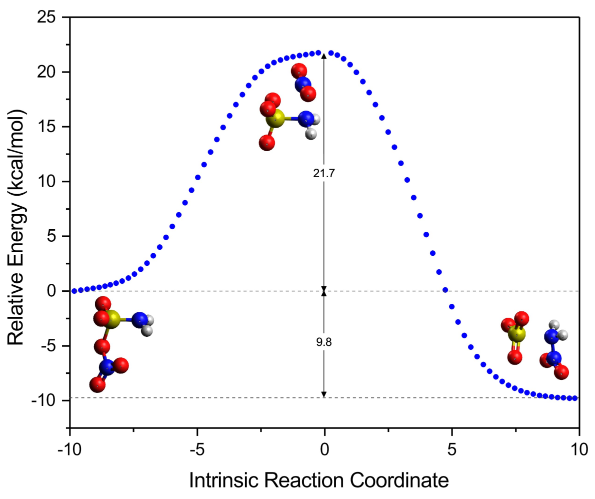

Figure S19: IRC for $TS2_{CD}$. Calculated with M06-2X/def2-SVP/PCM.

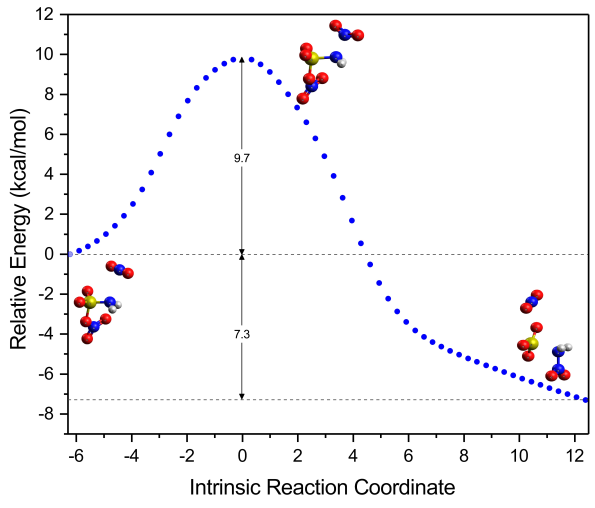

Figure S20: IRC for $TS3_{C}$. Calculated with M06-2X/def2-SVP/PCM.

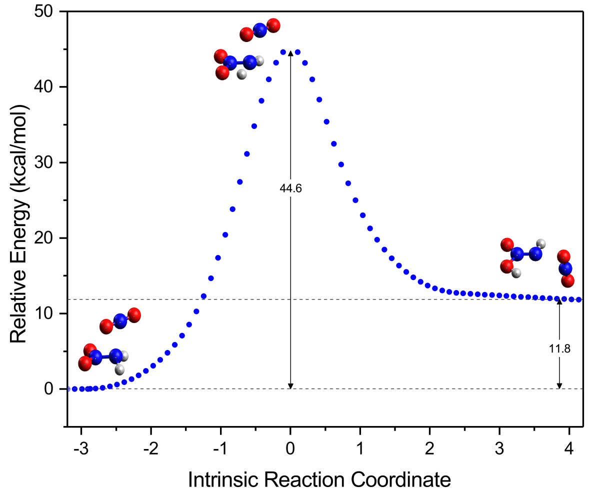

Figure S21: IRC for $TS4_{D}$. Calculated with M06-2X/def2-SVP/PCM.

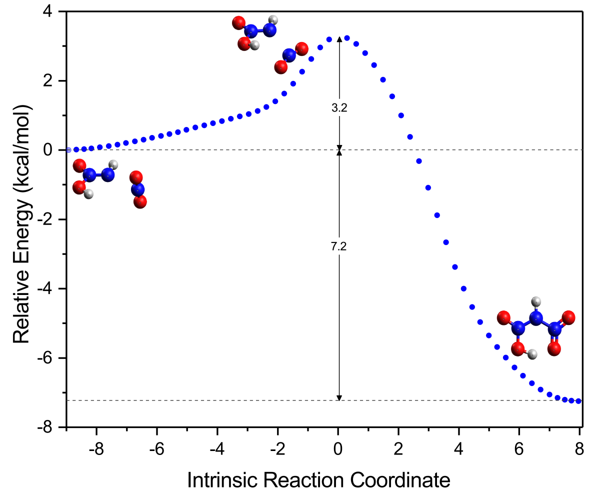

Figure S22: IRC for $TS6_{D}$. Calculated with M06-2X/def2-SVP/PCM.

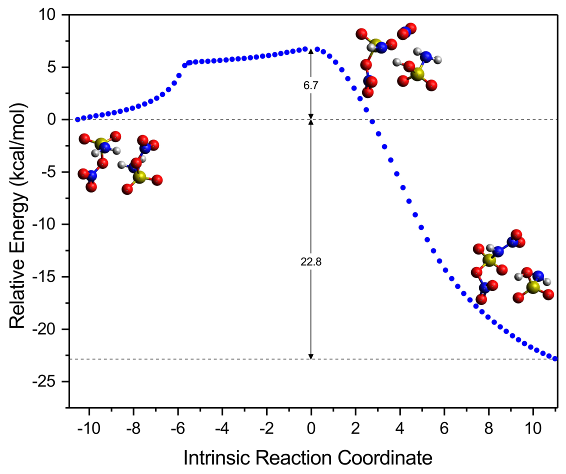

Figure S23: IRC for $TS2_{DIM}$. Calculated with M06-2X/def2-SVP/PCM.

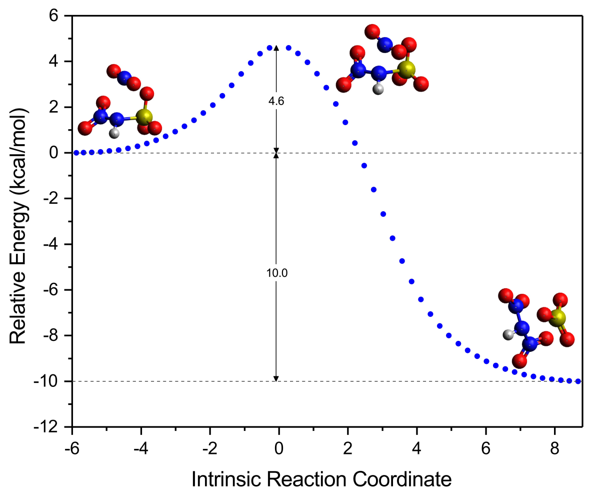

Figure S24: IRC for $TS9_{DIM}$. Calculated with M06-2X/def2-SVP/PCM.

2.3 Potential surface scan for calculations with PCM

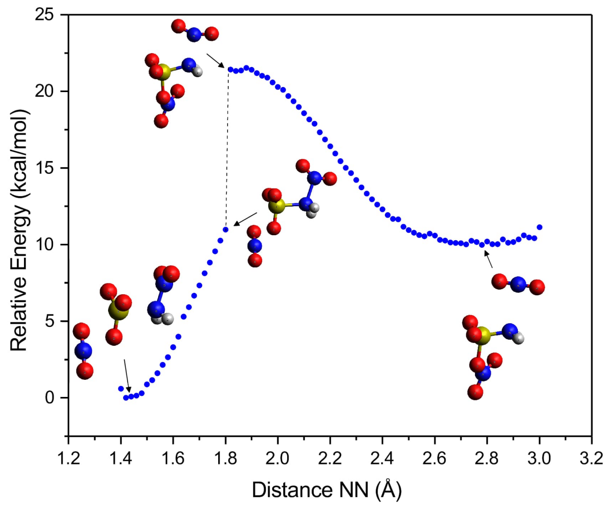

Figure S25: Relaxed PEC representing the approximation of NO_2^+ to $SS1_C$ forming $SS2_C$, $TS3_C$ and $SS4_C$. M06-2X/def2-SVP/PCM.

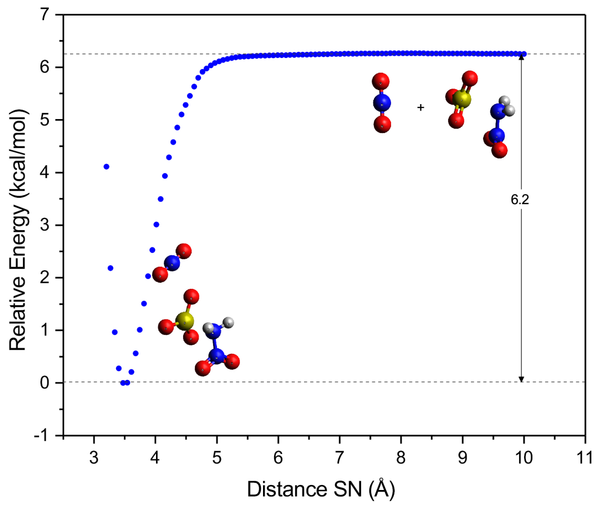

Figure S26: Rigid PEC representing the $SS4_c$ dissociation. M06-2X/def2-SVP/PCM.

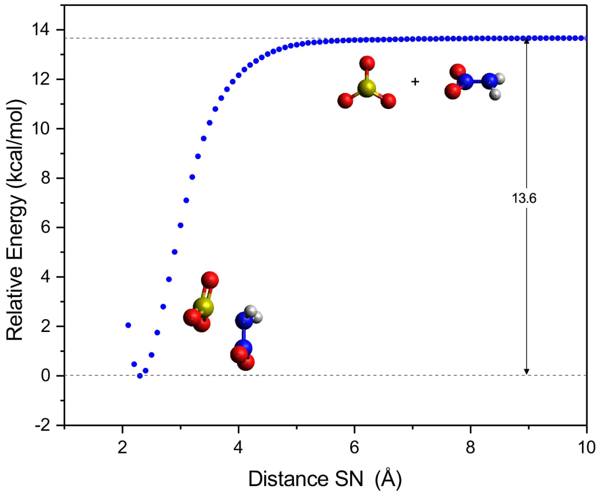

Figure S27: Rigid PEC representing the $SS1_D$ dissociation. M06-2X/def2-SVP/PCM.

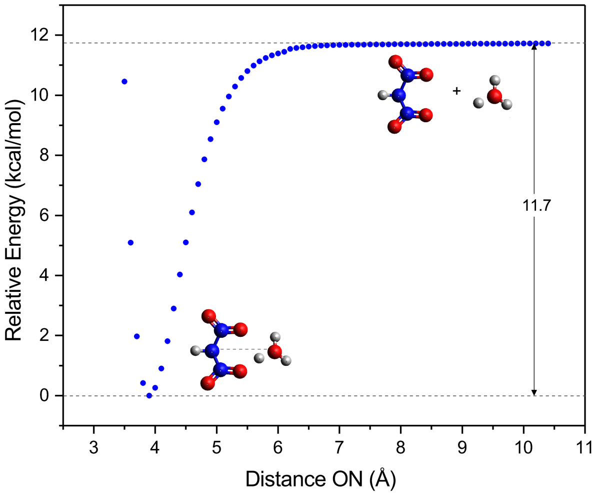

Figure S28: Rigid PEC representing the $SS8_D$ dissociation. M06-2X/def2-SVP/PCM.

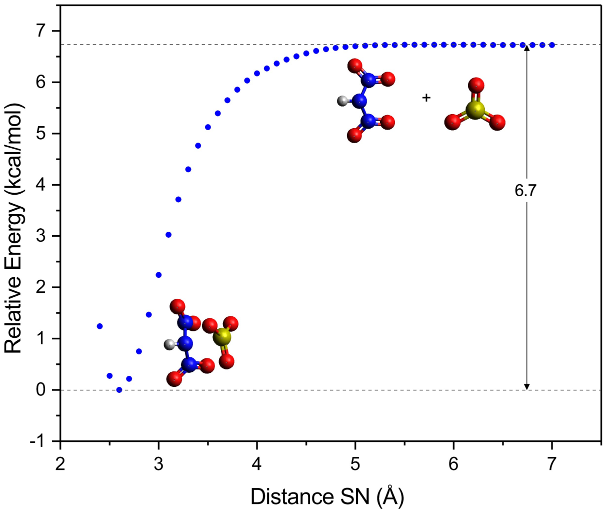

Figure S29: Rigid PEC representing the $SS9_{DIM}$ dissociation. M06-2X/def2-SVP/PCM.

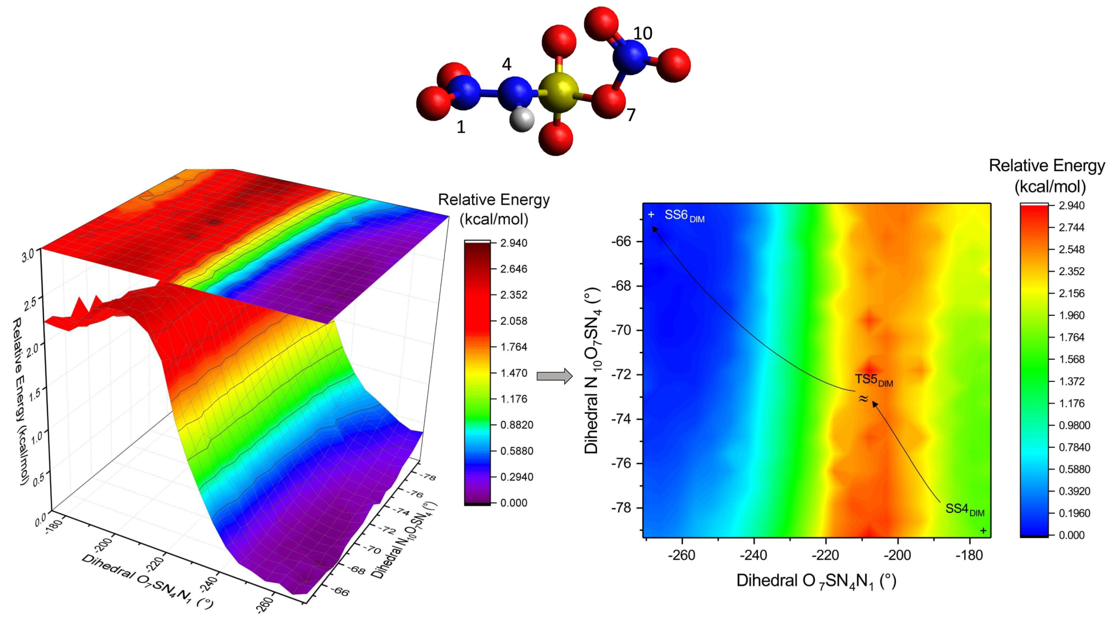

Figure S30: Rigid PES representing the rotaion of the internal dihedrals of $SS4_{DIM}$. M06-2X/def2-SVP/PCM.

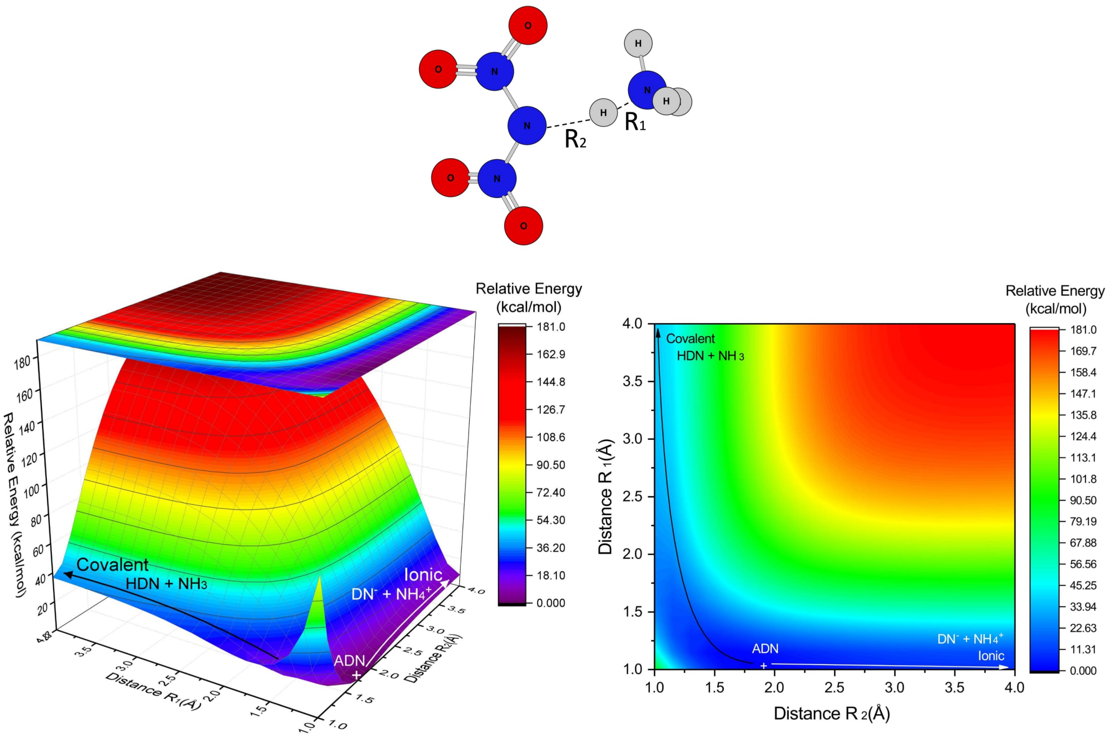

Figure S31: Rigid PES representing the formation of ADN by approximating the NH_3 to HDN. M06-2X/def2-SVP/PCM.

2.4 Stationary states cartesian coordinates for calculations with PCM

10

ss1 from G1

O -0.180198 -1.276005 1.263170

O -0.180198 -1.276005 -1.263170

N -2.202528 -0.570186 0.000000

S -0.617411 -0.759053 0.000000

O -0.180198 0.905570 0.000000

H -2.616964 -0.231313 -0.867375

H -2.616964 -0.231313 0.867375

N 1.177457 1.207860 0.000000

O 1.938675 0.291421 0.000000

O 1.387919 2.372989 0.000000

10

ss1 from G2

O 0.999167 -0.815427 -1.263050

O 0.997295 -0.813232 1.264642

N 2.005119 1.075934 -0.000070

S 0.968979 -0.138699 0.000177

O -0.482009 0.786585 -0.001638

H 2.077574 1.607349 0.866722

H 2.078967 1.605913 -0.867616

N -1.684580 0.088151 -0.000339

O -1.622577 -1.101726 0.000081

O -2.629874 0.800967 0.000082

10

ss1 from G3

O 0.927421 0.469993 1.427216

O -0.507460 -0.810436 -0.202631

N 1.181358 1.085736 -0.967375

S 1.022106 -0.086322 0.110326

O 1.864554 -1.190194 -0.234901

H 1.275840 0.786942 -1.937001

H 0.609293 1.913366 -0.800312

N -1.651811 -0.042696 -0.042342

O -1.505152 1.131646 0.118364

O -2.647571 -0.678564 -0.103033

10

ss1 from G4

O 0.507124 -0.810037 -0.207135

O -0.927153 0.459351 1.430999

N -1.179443 1.093460 -0.958883

S -1.022165 -0.086993 0.110018

O -1.865744 -1.187331 -0.243760

H -0.603653 1.917153 -0.785566

H -1.271247 0.801309 -1.931441

N 1.651267 -0.042782 -0.043102

O 1.504546 1.131596 0.117741

O 2.647074 -0.678744 -0.101518

10

ss1 from G5

O 1.351952 1.430354 -0.009225

O 0.623621 -0.505423 1.356265

N -0.647541 -0.169401 -1.040541

S 1.230933 0.008085 0.157106

O 2.007194 -0.870224 -0.672522

H -0.720674 -1.095055 -1.475625

H -0.662859 0.591221 -1.729164

N -1.727750 0.003707 -0.135276

O -1.977077 1.137325 0.151443

O -2.216235 -1.000241 0.289266

10

ss1 from G6

O 1.353898 1.430199 -0.008389

O 0.623167 -0.505484 1.355883

N -0.647869 -0.166831 -1.040923

S 1.231252 0.007980 0.157111

O 2.006363 -0.870853 -0.673030

H -0.720433 -1.091588 -1.478002

H -0.663804 0.595304 -1.727837

N -1.728052 0.003625 -0.135220

O -1.979411 1.136561 0.152465

O -2.214561 -1.001543 0.288703

10

ss1 from G7

O 0.506443 -0.802975 -0.238146

O -0.907944 0.422832 1.444184

N -1.193575 1.116175 -0.925150

S -1.020431 -0.090385 0.111243

O -1.867229 -1.182529 -0.259688

H -0.611449 1.933530 -0.743371

H -1.309686 0.849428 -1.902207

N 1.650528 -0.040500 -0.051268

O 1.504672 1.133465 0.113398

O 2.645229 -0.679109 -0.097170

10

ss1 from G8

O -0.981633 1.293271 -0.733973

O -0.902241 -1.176278 -0.911030

N 0.644127 -0.022833 1.042280

S -1.238077 0.001354 -0.158159

O -2.114344 -0.108687 0.974668

H 0.690430 -0.884845 1.596084

H 0.680162 0.819492 1.626546

N 1.735652 0.000003 0.134008

O 2.126525 -1.068918 -0.231309

O 2.094217 1.086048 -0.214120

13

ss2-c

O 0.064046 0.247011 1.443433

O 1.829267 0.599436 -0.316500

N -0.620826 -0.232390 -0.899258

S 0.198738 0.755555 0.110291

O -0.041042 2.115148 -0.257051

H -0.600087 0.092639 -1.870072

H -0.318469 -1.208104 -0.810197

N 2.473488 -0.631980 -0.093040

O 1.772456 -1.579896 0.080700

O 3.646995 -0.541698 -0.133753

N -3.184464 -0.505739 0.054627

O -2.782249 -0.499484 1.085857

O -3.606804 -0.513347 -0.967772

13

ts3-c

O 0.139548 0.449012 1.478704

O 1.516158 0.544639 -0.596472

N -0.867088 -0.419611 -0.685806

S 0.122099 0.825981 0.103019

O -0.366362 2.061689 -0.407642

H -0.976010 -0.234775 -1.696275

H -0.465972 -1.363402 -0.548007

N 2.420166 -0.653037 -0.069342

O 1.798644 -1.536344 0.366924

O 3.523204 -0.389206 -0.272598

N -2.559952 -0.547038 -0.028826

O -2.548362 -0.357881 1.102350

O -3.245766 -0.806873 -0.910792

13

ss4-c

O -0.113332 1.484436 0.563174

S 0.016944 0.065941 0.745721

O 0.975942 -0.460385 1.673675

O -1.100294 -0.767692 0.353697

N -3.274291 0.004546 -0.465970

O -2.956550 1.064708 -0.494821

O -3.632805 -1.041552 -0.451766

N 1.203416 -0.308962 -0.991562

H 1.108094 -1.284747 -1.300450

H 0.940125 0.355400 -1.731251

N 2.575384 -0.071949 -0.626239

O 2.891026 1.077218 -0.588075

O 3.204652 -1.043127 -0.345063

6

ss2-d: h2nno2

N -1.221120 0.000000 -0.083589

H -1.628129 -0.872395 0.241532

H -1.628130 0.872395 0.241531

N 0.128963 0.000000 -0.000631

O 0.681335 -1.076241 0.006655

O 0.681335 1.076241 0.006655

9

ss3-d

N -0.479993 -0.727948 0.751712

H -0.667730 -1.729957 0.697193

H -0.469955 -0.374818 1.710985

N 1.858878 0.147912 -0.140673

O 1.428514 1.167442 -0.183163

O 2.330848 -0.852344 -0.114127

N -1.438458 -0.037451 0.023381

O -1.542122 1.137516 0.270018

O -2.022904 -0.649216 -0.828866

9

ts4-d

N -0.496258 -0.571392 0.770474

H -0.488300 -1.583381 0.919909

N 1.923132 0.106904 -0.149574

O 2.339569 -0.899315 0.045924

O 1.553690 1.129010 -0.362263

N -1.469239 -0.134216 0.000219

O -1.588926 1.108024 0.329479

O -2.113577 -0.697129 -0.818879

H -0.741203 0.649582 1.178169

9

ss5-d

N -0.398996 -0.511203 -0.200117

H -0.487636 -1.467239 -0.559382

N 2.137127 0.001762 0.001252

O 2.168424 -0.475900 0.998732

O 2.155428 0.485921 -0.993509

N -1.569560 -0.093938 -0.035014

O -1.694286 1.164406 0.434931

O -2.616896 -0.652523 -0.234119

H -0.793724 1.515663 0.548261

9

ts6-d

N 0.235883 -0.686910 0.408004

H 0.298458 -1.704995 0.294930

N -1.665169 0.052766 -0.070642

O -2.257738 -0.897729 -0.000747

O -1.493353 1.152673 -0.234445

N 1.333925 -0.161853 0.043640

O 1.401079 1.172580 0.146403

O 2.303201 -0.700222 -0.382597

H 0.743549 1.458565 0.809148

9

ss7-d: HDN-H

N 0.001903 -0.723011 0.000267

H 0.012833 -1.751295 0.000159

N 1.249636 -0.046139 0.000007

O 2.183299 -0.753761 -0.000168

O 1.203992 1.164882 0.000060

N -1.198758 -0.118450 0.000011

O -1.234502 1.159709 -0.000061

O -2.168392 -0.762430 -0.000102

H -0.257475 1.497302 0.000013

12

ss8-d: HDN-H3O+

N -1.074109 0.129976 0.441834

H -2.089844 0.263905 0.469022

N -0.730391 -1.161052 -0.026861

O -1.644354 -1.921598 -0.015496

O 0.409374 -1.368678 -0.325437

N -0.419769 1.273119 -0.014216

O 0.764442 1.219306 -0.261583

O -1.101220 2.243579 -0.062420

H 1.910499 0.189701 0.001695

O 2.786846 -0.314109 0.109124

H 2.614765 -1.195170 0.496891

H 3.413769 0.179268 0.673598

8

ss8-d: HDN

N -0.000011 -0.604827 0.365831

H -0.000004 -1.602058 0.139509

N 1.229922 -0.006962 0.012529

O 2.102356 -0.810505 -0.162268

O 1.301532 1.181312 -0.017453

N -1.229922 -0.006955 0.012518

O -1.301481 1.181320 -0.017510

O -2.102396 -0.810467 -0.162226

20

ss1-dim

O -0.538633 -1.601846 1.087474

O -1.235231 0.594314 0.459221

N -1.550971 -1.485442 -1.177721

S -1.657802 -1.075878 0.357493

O -3.015194 -1.146977 0.803922

H -2.299927 -1.152831 -1.784604

H -0.607651 -1.532178 -1.567819

N -2.034082 1.539965 -0.183574

O -2.811161 1.124751 -0.987011

O -1.810030 2.645425 0.164104

H 0.464678 1.042612 1.711382

O 1.254540 -0.344454 -0.749005

O 0.956264 2.041652 -0.721571

O 1.388307 -2.471187 -0.948709

N 1.789484 -1.546150 -0.320594

N 1.464412 1.039477 1.502518

S 1.833872 1.134804 -0.046873

O 2.543149 -1.520813 0.599472

H 2.058068 0.427860 2.062328

O 3.253715 1.108730 -0.224847

20

ts2-dim

O -0.539876 0.697122 -1.569415

O -2.388233 -0.391114 -0.251849

N -0.568412 1.290699 0.803312

S -1.429568 1.055726 -0.480095

O -2.517596 1.966982 -0.728009

H -1.135095 1.617734 1.590748

H 0.551589 0.113969 1.016276

N -2.062573 -1.430164 0.593559

O -1.512000 -1.171491 1.619043

O -2.447814 -2.483069 0.203023

H 1.303904 -0.139524 -2.006186

O 1.489324 -0.296713 0.988748

O 0.320260 -1.928327 -0.408254

O 1.613714 2.565312 1.612411

N 1.448087 2.338706 0.531625

N 2.107481 -0.441706 -1.450992

S 1.637701 -1.363802 -0.174889

O 1.563842 2.249384 -0.573499

H 2.834454 -0.906780 -1.992535

O 2.748745 -2.215451 0.173914

20

ss3-dim

O 0.572513 0.235003 -1.373068

O 1.982609 1.484374 0.241685

N 1.451639 -0.861853 0.730749

S 1.778320 0.120666 -0.604554

O 3.050041 -0.165705 -1.185133

H 2.063636 -0.787946 1.548642

H -1.117333 -0.069131 1.565282

N 0.699156 2.164572 0.676238

O 0.203992 1.652566 1.609657

O 0.468063 3.107596 0.035666

H -1.178923 -0.758966 -1.846087

O -1.421439 -0.717556 0.903759

O -1.875905 1.384364 -0.265976

O 0.928822 -2.830873 1.518681

N 0.954688 -2.149996 0.537002

N -2.093535 -0.929177 -1.423890

S -2.387115 0.031171 -0.144204

O 0.580315 -2.426587 -0.568067

H -2.870514 -0.914626 -2.083401

O -3.768982 -0.156125 0.227172

12

ss4dim

O 0.150062 0.924590 1.412549

O 1.385670 0.327529 -0.703339

N -0.863762 -0.605245 -0.358336

S -0.007079 0.819312 -0.002543

O -0.445282 1.903411 -0.824101

H -0.665261 -1.065441 -1.251607

N 2.288438 -0.602864 0.021912

O 1.779613 -1.277744 0.837435

O 3.377930 -0.505931 -0.391352

O -2.857929 -1.489009 -0.566139

N -2.220031 -0.621665 -0.046922

O -2.596811 0.212763 0.731911

12

ss6dim

O 0.153679 1.005010 1.508706

O 1.496699 0.251761 -0.481427

N -1.107898 0.463365 -0.623439

S 0.216464 1.159526 0.094298

O 0.433991 2.387064 -0.597683

H -1.186587 0.540051 -1.640573

N 1.622599 -1.107181 -0.071258

O 0.642985 -1.613274 0.368336

O 2.703023 -1.526943 -0.262484

O -2.571005 -1.126273 -0.918562

N -1.919910 -0.554528 -0.092313

O -1.914418 -0.715352 1.088223

12

ss8dim

O 1.363034 1.683118 -0.814640

O -0.920449 0.777635 -0.999780

N 0.581372 -0.205186 0.790857

S 0.153273 1.279627 -0.124657

O -0.296944 2.096090 0.989092

H 0.933712 0.001202 1.728341

N -1.984688 -1.006308 0.032876

O -1.559173 -1.734065 -0.686583

O -2.492709 -0.397163 0.808732

O 2.238487 -1.659746 0.864436

N 1.420245 -1.106133 0.175194

O 1.229681 -1.297350 -1.002047

12

ts9dim

O -1.375521 -1.775220 0.441242

O -1.131715 0.563980 1.218733

N 0.392178 -0.196386 -0.686890

S -1.315240 -0.368273 0.115154

O -2.089650 0.112195 -1.010549

H 0.363485 -0.318021 -1.706731

N 0.718461 1.813120 -0.052614

O 1.472396 1.690712 0.771815

O 0.028656 2.372307 -0.744301

O 2.037491 -1.666622 -0.941313

N 1.374801 -1.039063 -0.162680

O 1.468626 -1.026517 1.036817

12

ss10dim

O -1.608374 -1.618099 0.131943

O -1.356208 0.587437 1.239253

N 0.759859 0.072276 -0.655312

S -1.721746 -0.190603 0.090301

O -2.251709 0.459621 -1.069887

H 0.943041 0.142056 -1.664609

N 1.082425 1.351126 -0.041989

O 1.642186 1.344174 1.002692

O 0.677150 2.264857 -0.689630

O 2.064208 -1.708301 -0.917674

N 1.453457 -1.069754 -0.116052

O 1.274586 -1.275682 1.042461

12

ADN

N -0.057428 -0.213075 -0.106608

H -1.545583 -1.000107 -0.013930

N -0.209306 1.136502 -0.069293

O -1.366367 1.481312 0.131487

O 0.690899 1.910398 -0.293400

N 1.205802 -0.711776 0.021726

O 2.112040 -0.069068 0.501539

O 1.300766 -1.874670 -0.320818

N -2.592108 -1.194092 0.084090

H -2.782620 -1.774008 0.905259

H -2.963141 -1.655352 -0.750416

H -3.036072 -0.277221 0.199215

2.5 Frequency tables for calculations with PCM

Table S3: Frequencies for all stationary states and transition states for calculations under implicit solvent - PCM.

SS1 from G1 (SS1c)				
55.4898	105.5242	184.9243	296.1773	319.8351
386.2314	430.1919	473.3883	489.3179	518.0279
560.2479	746.5267	810.9976	819.8227	912.5780
974.7065	1060.7095	1234.0231	1452.2046	1456.2631
1526.4128	1863.6297	3536.4582	3662.5904	

SS1 from G2 (SS1c)				
66.8950	108.7507	185.9620	295.6090	326.4147
388.2597	437.4925	470.6713	491.5504	515.3416
564.0432	745.0936	814.4789	820.8909	913.1485
976.5612	1055.5951	1234.9522	1453.0625	1453.7410
1529.4810	1868.8978	3536.5370	3662.9349	

SS1 from G3 (SS1c)				
79.8717	141.3301	203.5761	319.3126	327.7864
389.9398	406.3683	468.5139	515.4147	527.6857
556.7345	755.7208	807.9175	838.0175	911.4343
979.6015	1064.2711	1241.0900	1451.0734	1470.9709
1527.4829	1866.9754	3526.8736	3652.8876	

SS1 from G4 (SS1c)				
79.3755	141.7544	205.0833	318.7646	328.8569
390.3190	407.3430	469.9791	515.2678	532.5494
558.6769	755.6011	807.4859	838.5821	911.4439
979.4972	1065.6471	1241.1684	1451.0363	1470.7311
1528.1305	1866.9511	3524.3290	3649.4562	

SS1 from G5 (SS1d)				
43.0443	75.8359	117.8819	191.6527	216.6746
264.2026	483.9945	504.3760	524.8763	533.9531
711.9414	725.0789	805.9252	964.8228	1097.8864
1113.8251	1257.7068	1405.3116	1415.3562	1483.6864
1571.7497	1855.6047	3456.8631	3570.8237	

SS1 from G6 (SS1d)				
42.7962	75.3061	118.0483	191.4289	216.4324
263.9411	483.9328	504.3145	524.8775	533.8428
711.7567	724.8566	805.8281	964.7908	1097.6508
1113.6743	1257.4423	1405.3138	1415.2348	1483.7151
1571.6273	1855.5717	3456.8638	3570.8279	

SS1 from G7 (SS1c)				
77.7178	138.7084	204.9380	314.2178	326.1104
389.4943	407.3470	463.6006	514.8714	526.2420
555.3209	754.9548	804.4374	841.6921	911.0068
978.9861	1061.3608	1240.5386	1451.1583	1469.9751
1525.1155	1867.4720	3525.4158	3651.0323	

SS1 from G8 (SS1d)				
33.5725	92.7142	114.3541	191.7128	219.0914
267.4660	481.2372	509.1805	523.3304	535.8675
721.5987	740.6517	810.0770	968.0277	1104.6442
1125.5657	1255.4102	1409.2512	1413.9903	1481.0809
1571.1953	1851.3746	3463.1958	3572.6408	

TS2cd				
-227.5647	81.4932	145.4624	189.8377	251.6588
329.8309	339.1886	470.6145	500.3017	530.8346
539.7308	548.1788	606.8448	755.2163	904.9383
1049.2939	1066.6149	1253.5142	1322.5939	1509.8420
1539.0193	2504.8155	3495.3867	3596.1440	

SS2c				
47.2949	50.1450	62.3346	81.4412	97.5021
133.0382	137.2863	215.7166	326.3268	348.1496
398.6187	414.6127	511.8520	556.0626	560.0390
589.0443	653.1976	734.0909	780.5490	802.4334
835.7019	903.9661	945.1192	1120.8393	1235.3294
1447.1925	1475.5382	1544.0410	1581.8188	1886.2956
2609.9721	3485.9308	3592.2299		

TS3c				
-240.3038	44.2871	49.4548	65.4936	98.5196
144.5877	175.8887	225.9664	304.6128	335.3711
355.4849	430.2260	527.5236	530.4804	591.6297
615.5447	698.1372	716.6217	778.0275	802.2607
836.7516	919.7526	1140.7658	1201.2158	1237.8068
1457.1325	1473.1150	1500.7266	1515.7662	2061.7670
2157.6210	3373.6306	3469.6592		

SS4c				
-10.1421	28.0151	41.4861	60.7539	72.2295
123.9450	142.6028	169.2799	224.3732	237.7346
291.2503	492.0714	513.0405	522.8521	537.4873
592.4788	651.5650	708.5029	758.0631	808.3084
944.4309	1099.0345	1149.6589	1243.1425	1372.7852
1423.4222	1473.3115	1562.6888	1581.1255	1868.4166
2610.8511	3437.7623	3543.6308		

SS2d				
457.2449	533.7891	606.1909	750.5922	835.3604
1113.6256	1236.5479	1505.9669	1578.3051	1762.3045
3563.0998	3707.4520			

SS3d				
46.0874	56.6984	82.0487	151.0352	201.1755
512.8222	560.6343	619.2870	649.3512	722.8300
793.9662	910.0031	1079.5186	1271.6694	1499.5800
1577.3230	1594.0117	1803.4179	2606.4553	3508.8767
3625.6623				

TS4d				
-1978.2950	49.3147	66.3202	73.5785	123.5451
181.3026	559.2373	596.3642	649.8116	664.3560
760.1528	861.0768	1102.7882	1152.9675	1284.4985
1462.7712	1576.7637	1758.4952	2319.1408	2605.2812
3545.9604				

SS5d				
40.9042	57.8987	153.5613	154.9772	161.0277
429.7728	573.9601	643.5344	654.6303	705.4977
764.1775	846.7867	999.4317	1350.9504	1381.1417
1539.5096	1582.4529	1782.2597	2611.3255	3520.8501
3801.2004				

TS6d				
-260.3864	59.2014	75.8557	133.4103	329.3284
442.1439	516.9705	646.2978	665.4991	704.3407
772.2125	815.6658	1009.1223	1336.7490	1360.2040
1443.3196	1535.7771	1792.3584	2397.0446	3510.2318
3738.2972				

SS7d				
115.5930	138.5858	295.8604	465.1508	481.9296
500.6974	726.7355	758.5647	784.4584	860.2872
878.3941	982.4014	1059.1513	1217.9145	1450.8252
1464.9563	1559.3315	1849.0850	1937.9257	2686.6165
3508.5254				

SS8d				
-4.7782	62.1380	110.7079	136.8080	192.2212
197.9529	289.3085	349.7076	443.2291	482.1917
493.9920	634.2485	790.6709	802.8245	812.7893
886.4815	1007.4157	1013.9872	1078.4298	1373.4216
1434.2571	1515.5344	1614.2412	1629.5110	1810.9587
1859.4342	2899.9357	3535.5420	3707.7701	3772.3509

SS9d/SS11dim (HDN)				
16.2702	121.5216	269.6560	443.3374	472.6138
639.5536	791.8864	802.3599	811.9676	887.0386
996.8732	1070.5232	1404.7626	1411.1633	1503.1188
1821.8762	1854.5232	3565.8523		

SS1dim				
23.0227	61.4013	70.0777	96.3022	103.6922
107.5137	122.0516	133.7181	148.7314	159.6572
197.5946	208.2530	307.5319	320.9278	327.2889
329.9320	378.7267	395.8745	397.6368	409.5342
466.1693	471.7857	494.6400	513.7303	520.0620
527.7346	544.9543	551.4403	753.6227	756.3119
792.0890	806.2756	827.1609	834.8396	899.4903
918.6956	976.1360	985.0233	1067.7408	1071.2549
1232.8478	1241.9613	1444.4461	1452.2058	1465.4796
1473.1977	1518.5755	1523.5790	1859.9691	1876.2304
3512.7246	3520.2608	3644.8980	3647.6864	

TS2dim				
-248.4202	28.0210	50.8588	63.6562	77.7625
103.7516	105.1954	110.4636	119.2611	142.3242
150.4387	188.9005	217.1775	225.2197	263.3555
279.6687	343.2836	381.8838	391.3019	405.0535
424.1695	506.1151	512.1506	519.1349	543.7178
551.1100	554.0201	612.5922	643.2809	648.3270
736.9836	770.3534	843.5366	868.4454	872.7412
927.0572	946.5377	1013.1098	1103.8272	1145.2203
1193.0655	1217.3700	1357.4870	1387.6907	1444.2552
1454.0864	1515.6482	1524.7921	1846.5131	2499.0478
2759.4047	3509.4114	3540.9806	3637.8327	

SS3dim				
45.1586	52.6048	61.3721	75.5769	87.4397
93.3878	100.3449	110.3256	135.7456	141.0642
168.7566	189.9097	231.5260	259.4388	291.2591
307.8756	351.4370	386.5356	410.4401	429.4491
457.4443	490.5014	511.6506	517.3293	538.8649
554.9974	591.7023	625.7830	630.3815	658.4007
798.0586	805.6558	836.3868	840.7149	887.1657
904.5505	930.1243	1036.2323	1098.4912	1154.9602
1217.6569	1226.4602	1381.7480	1387.4015	1454.3568
1465.3478	1474.5962	1533.1468	1824.2085	1989.5535
3521.8144	3532.7311	3641.6663	3758.1357	

SS4dim				
43.7396	56.3958	76.6022	119.7550	183.0968
188.1465	285.4731	290.3510	381.7925	417.2336
440.5946	530.3500	555.1400	571.8785	655.5519
744.8510	795.9810	806.6977	813.1395	887.6872
905.3472	1045.4253	1232.6654	1388.5422	1451.5589
1467.6230	1479.4077	1813.7705	1963.5446	3539.9822

SS6dim				
43.0620	64.0539	112.4921	119.8882	194.4489
230.4349	288.3825	326.7440	377.4353	403.1849
421.5391	513.7579	560.2440	570.3881	731.1162
773.1046	796.2259	814.8194	826.1798	896.0197
929.8122	1004.9216	1234.2367	1389.8766	1445.6575
1463.6532	1505.3298	1837.1250	1910.7084	3549.9886

SS8dim				
40.5565	71.6634	83.4756	104.2159	117.6815
163.9910	195.4499	211.5538	322.9444	358.5862
413.1509	508.8134	529.3432	550.5341	600.8904
631.3091	680.4614	775.9860	832.2848	875.9255
1044.5702	1094.5017	1275.9468	1347.2611	1380.8394
1476.7226	1559.0702	1778.8126	2582.2143	3555.8628

TS9dim				
-313.0040	63.7425	73.6293	100.2103	139.5119
159.7124	179.9261	275.9169	312.2607	324.7079
350.1101	516.4545	527.2600	543.4032	600.4265
637.1166	725.4488	775.6542	855.8947	917.2254
1025.3296	1087.3737	1314.0070	1346.9399	1375.5162
1448.1392	1501.9199	1804.4619	2391.5192	3491.2378

SS10dim				
36.0826	39.9227	61.0524	78.6447	98.9043
109.7997	131.2378	191.7872	265.6046	436.0259
454.5203	498.7580	514.2658	516.3312	683.2261
755.5083	793.2957	869.3297	877.9344	922.4690
1066.5866	1112.8193	1400.8005	1415.4941	1421.0526
1432.1508	1500.5622	1855.9453	1885.3892	3490.1559

ADN				
36.1379	69.3684	96.3166	119.4638	143.4070
225.8833	319.9171	339.2906	364.4571	507.8476
513.5474	800.8862	811.9240	821.3305	899.6209
1060.3138	1168.7795	1325.6369	1354.2301	1456.6848
1479.7055	1496.3611	1656.8003	1677.6266	1703.0025
1740.8244	2709.9314	3472.1610	3581.6261	3595.1810

2.6 Table of energies corrected with triple-zeta basis set for calculations with water

Table S4: Relative energies (kcal.mol^{-1}) for calculations under implicit solvent effect. M06-2X/def2-TZVP//M06-2X/def2-SVP

Species	ΔE	ΔH(0K)	ΔH(298.15K)	ΔG(298.15K)
$SS1_C$	-28.47	-27.25	-27.84	-16.95
$TS2_{CD}$	-11.29	-10.78	-11.33	-0.56
$SS2_C$	-28.95	-26.89	-27.22	-8.25
$TS3_C$	-14.84	-13.25	-14.13	6.24
$SS4_C$	-38.90	-36.67	-37.18	-18.28
$SS1_D$	-35.77	-34.03	-34.28	-24.90
$SS2_D$	-27.96	-28.03	-41.95	-43.88
$SS3_D$	-45.20	-44.31	-28.20	-30.14
$TS4_D$	16.43	13.78	13.70	19.60
$SS5_D$	-19.30	-18.57	-18.59	-12.66
$TS6_D$	-31.50	-31.45	-32.02	-24.51
$SS7_D$	-20.35	-19.67	-20.70	-11.60
$SS8_D$	-44.00	-40.76	-42.66	-23.40
$SS9_D$	-37.27	-35.14	-36.09	-28.65
$2SS1_C$	-56.93	-54.49	-55.67	-33.89
$SS1_{DIM}$	-61.01	-57.96	-58.67	-25.12
$TS2_{DIM}$	-32.35	-31.03	-31.69	1.40
$SS3_{DIM}$	-62.55	-59.90	-60.29	-27.65
$SS4_{DIM}$	-59.40	-57.66	-58.33	-38.02
$SS6_{DIM}$	-60.44	-58.41	-59.10	-40.92
$SS8_{DIM}$	-60.76	-58.97	-59.22	-39.81
$TS9_{DIM}$	-52.78	-51.41	-52.18	-31.27
$SS10_{DIM}$	-63.24	-61.80	-61.89	-43.72
$SS11_{DIM}$	-61.20	-60.55	-61.05	-53.94

3 Explicit Solvent: Water + PCM

3.1 Results and Discussion

Although the initial objective of our manuscript was to assess the impact of a polarized field on the mechanism, we also performed some calculations with explicit water molecules. The implementation of water was made only for D-Pathway, starting from Stationary State 3 at D-Pathway ($SS3_D$), as this step corresponds to the hydrogen abstraction, presenting a high energy barrier, of 40 kcal mol^{-1}, approximately.

The study started by doubling twice the amount (n) of explicit water molecules resulting in $n = 1, 2$ and 4. Initial structures were obtained from SOLVATOR algorithm on ORCA 6.0 software. The generated structures calculations were optimized in accordance with the methodology described in the main manuscript: the thermochemical properties of the stationary states were obtained through Density Functional Theory (DFT) within a hybrid meta-GGA approximation, M06-2X combined with the def2-SVP basis set. Frequency analysis was carried out to compute the thermodynamic quantities and to identify possible saddle points (defined in this work as Transition States - TS) during the optimization. Thus, the intermediates were classified as either minima, Stationary States (SS), or saddle point, Transition States (TS), based on the absence or presence of only one imaginary frequency vibrational mode, respectively. To ensure the connection between the SSs, Intrinsic Reaction Coordinate (IRC) calculations were performed for each of the previously characterized TS. If saddle points were not found, the PES was scanned between the reactants and products.

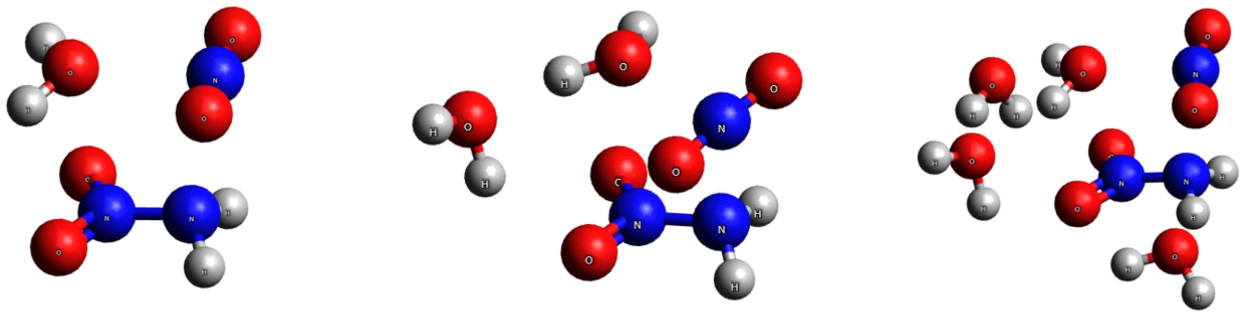

Figure S32: $SS3_D$ geometries solvated with water molecules. Calculated with M06-2X/def2-SVP/PCM

Figure S32 shows the structures of $SS3_D$ obtained by adding explicit water molecules. From figure S32, it is possible to see that by adding only one molecule of water (Figure S32a), the H_2O orientates its oxygen atom in direction of the positive charge (located at $NO_2{}^+$) and its hydrogen atoms to the oxygen atoms from nitramine (NH_2NO_2). When a second molecule of water (Figure S32b) is added, one keeps aligned with $NO_2{}^+$, while the second one tries to keep the hydrogen bonds with the first water molecule and the oxygen from nitramine. In these two cases, once no water molecule interacts directly with the hydrogen atom from nitramine, it is not expected they would help directly to transfer the hydrogen atom from the nitrogen to oxygen. However, their presence could help reduce the barriers.

As the number of water molecules gets higher, e.g., four molecules (Figure S32c), it starts to have an interaction between the hydrogen atom from nitramine and the oxygen atom from the fourth water molecule. In this last case, the water molecule interacting directly with hydrogen could indeed help reduce the electronic barrier associated with the hydrogen abstraction.

Using the stationary states from Figure S32, and considering the mechanism obtained with PCM, a mechanism for each case was proposed and it is discussed in the next paragraphs.

Figure S33 shows mechanisms calculated considering 1 and 2 water molecules. As one

can see, the addition of 1 and 2 water molecules did not affect the mechanism significantly, maintaining the stationary states essentially the same as those obtained considering PCM. Also, the hydrogen abstraction barrier kept high ($\approx$ 45 kcal mol$_{-1}$), implying the fact that the presence of only a few molecules of water does not affect the electronic barrier, and they did not help with the hydrogen abstraction process, as predicted.

For both cases (n = 1 and 2), first the system passes through a TS ($TS4_D$) responsible for transferring the hydrogen from the nitrogen atom to the oxygen atom of nitramine, and subsequently, NO_2^+ binds to nitramine to form $SS7_D$ by overcoming a low barrier of 3.0 and 5.0 kcal mol^{-1}, approximately, for n = 1 and n = 2, respectively, in accordance with the mechanism proposed by applying PCM.

In the mechanism described in the main manuscript with PCM, it was necessary to add an explicit molecule (water) to correctly remove the proton and obtain dinitramidic acid (HDN). Now, since we start the mechanism with molecules of water already present in the system, we don't have the presence of $SS8_D$ but a transition state $TS8_D$, responsible for interacting one water molecule with the hydrogen producing HDN + H_3O^+. For n = 1, we were able to characterize the TS and calculate its IRC; however, for n = 2 we could not find the TS so we varied the distance between the oxygen from water and the nitrogen from H−HDN to obtain any information about the existence of a possible TS. As one can see in Figure S33e, when the distance O−H is close to 2.3 Å, there's a discontinuity in the plot associated to a changing in the geometry caused by the relaxation of the molecule; this point presents a geometry similar to $TS8_D$ obtained in the mechanism with 1 water molecule and an energy of 1.1 kcal mol^{-1} in relation to $SS7_D$, also similar to the result obtained for n = 1. In this sense, the presence of few molecules of water affects significantly neither the mechanism nor the electronic barriers, giving results in accordance with those obtained in the first version of the manuscript with PCM.

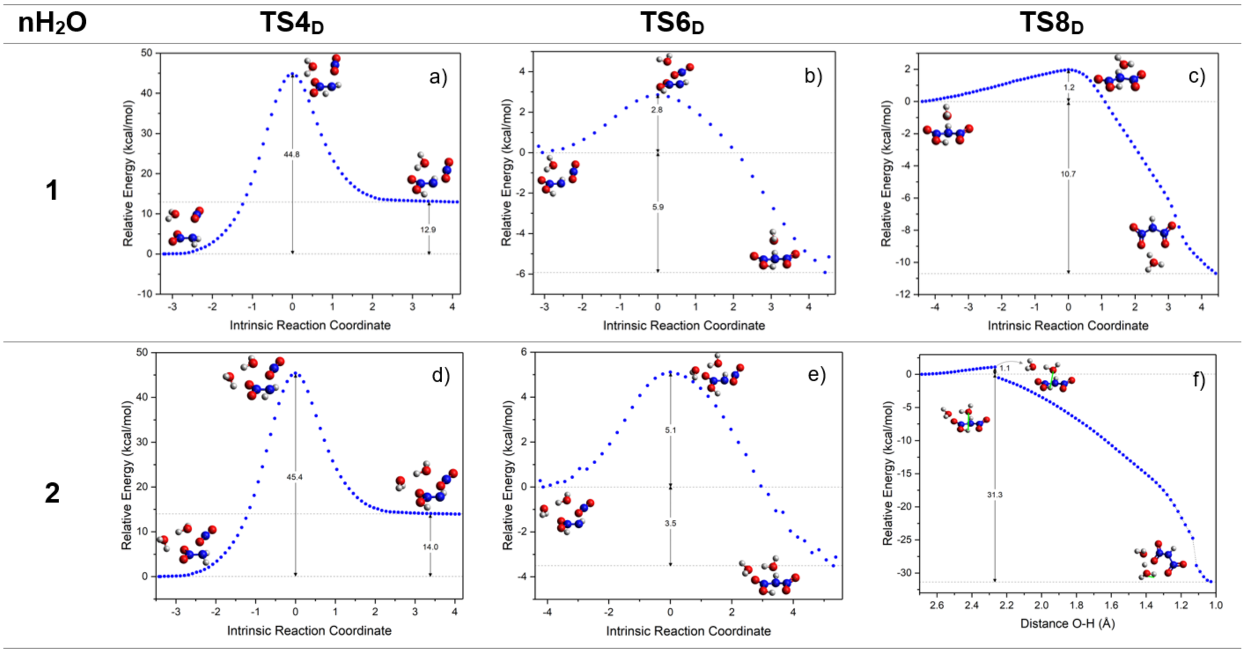

Figure S33: IRCs and Scan related to $TS4_D$, $TS6_D$ and $TS8_D$ for mechanisms involving 1 and 2 water molecules. Calculated with M06-2X/def2-SVP/PCM.

However, when adding more molecules of water, as explained earlier, it is expected the formation of nitric acid. This effect is observed for the mechanism with four explicit water molecules.

First, from SOLVATOR algorithm it was obtained the $SS3_D$ with 4 H_2O. This geometry was optimized with Gaussian 09. The optimization is shown in Figure S34a. A guess for $TS4_D$ was given and the IRC for the obtained TS is shown in Figure 34b. As one can see from Figure S34b, the left side led to a structure similar to the optimized SS3D, differing only by the relative position of the water molecules. However, when optimizing the $SS3_D$ obtained from the IRC, the geometry relaxed even more and led to a new SS, a SS containing HNO_3 (Figure S34c), and consequently it led to a new pathway (E-Pathway). In this sense, from $SS3_D$ the system has two options, pass through a TS ($TS4_D$) and produce $SS5_D$, or the system can relax to a new stationary state ($SS4_E$) 30 kcal mol^{-1} more negative.

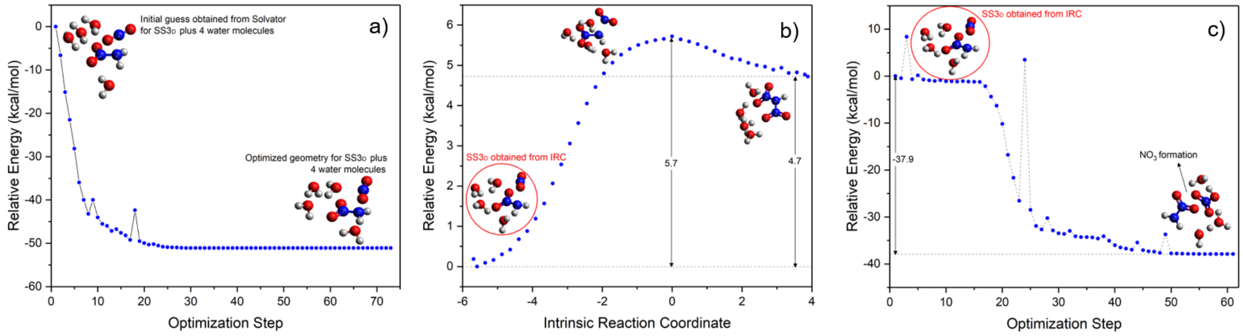

Figure S34: a) Optimization of $SS3_D$. b) IRC of $TS4_D$. c) Optimization of the $SS3_D$ obtained from IRC. Calculated with M06-2X/def2-SVP/PCM.

It is also important to point out that the right side of the IRC (Figure S34b) was also optimized and the optimization reduced the energy of the SS even more, from 1 kcal mol^{-1} to 36.8 kcal mol^{-1} relative to the TS. One important difference is the fact that the right side of the IRC led to the formation of the desired product: HDN, not being necessary to pass through other TS, as for the mechanisms containing one and two water molecules.

Finally, the mechanisms for 1, 2 and 4 water molecules are presented in the energy diagrams shown in Figure S354.

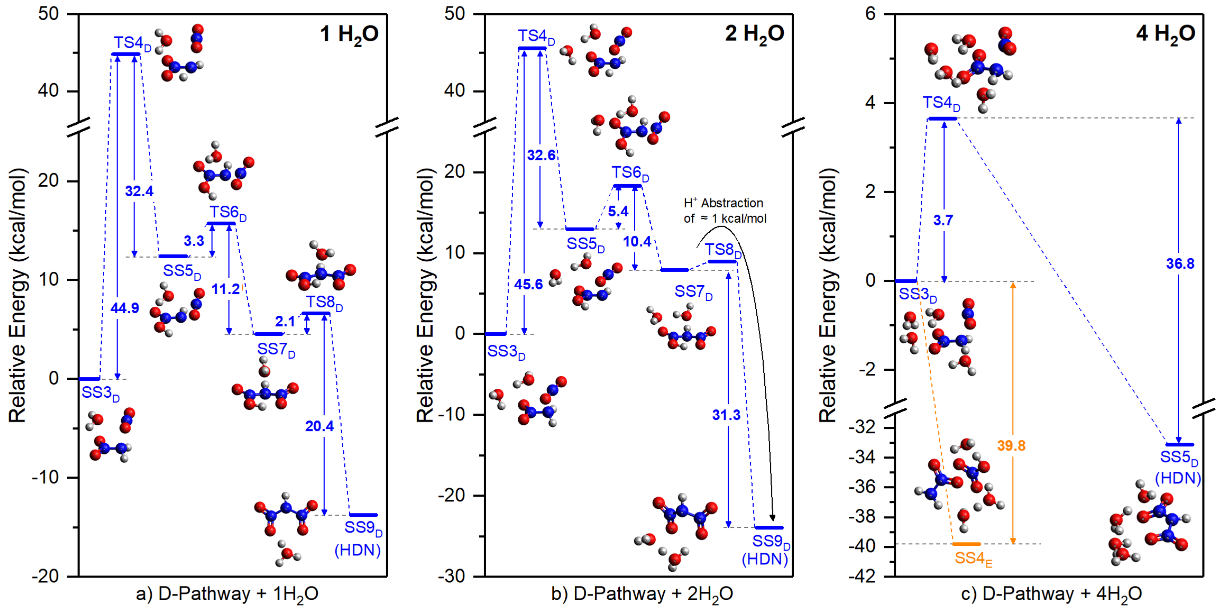

Figure S35: Relative energy diagrams for the three mechanisms studied: with a) 1 H_2O molecule, b) 2 H_2O molecule and c) 4 H_2O molecule. Calculated with M06-2X/def2-SVP/PCM

3.2 Stationary structures for calculations with explicit solvent

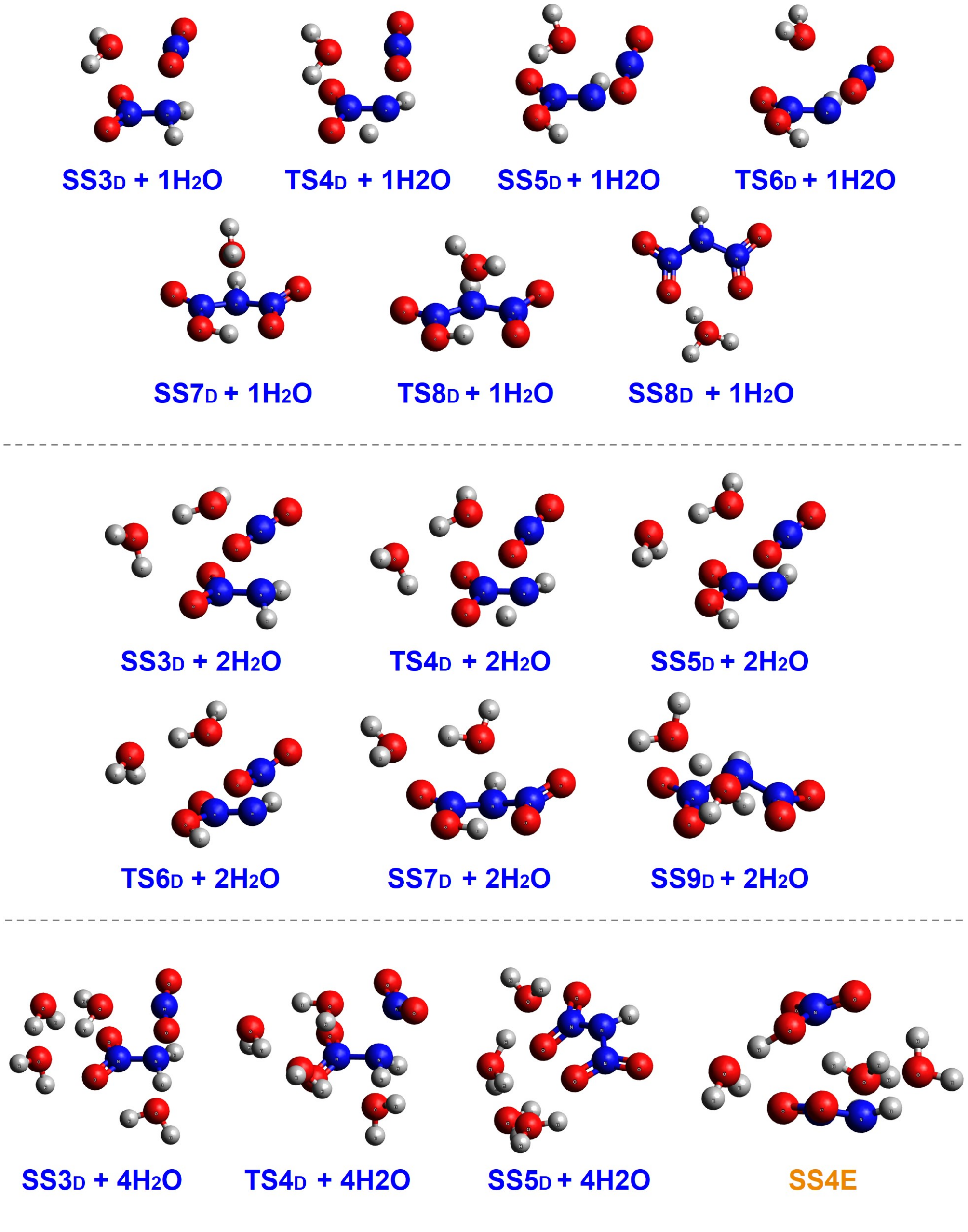

Figure S36: Stationary states for the mechanism under implicit solvent optimized with M06-2X/def2-SVP/PCM.

3.3 Stationary states cartesian coordinates for calculations with explicit solvent

12

ss3d + 1h2o

N -0.676342 -1.309031 0.031810

H -0.813100 -1.879353 -0.803465

H -0.793736 -1.822779 0.905943

N 1.832759 -0.251375 -0.001319

O 1.878733 -0.264036 1.104280

O 1.874386 -0.297097 -1.106199

N -1.536845 -0.232316 0.005961

O -1.825203 0.255005 1.069759

O -1.866333 0.172601 -1.077982

O 0.471082 1.659764 -0.023542

H 0.068496 2.059732 -0.805777

H -0.059976 1.981556 0.717610

12

ts4d + 1h2o

N -0.747558 -1.277192 0.090223

H -0.736253 -1.966030 -0.665307

H -1.012153 -0.848253 1.296660

N 1.877126 -0.266109 -0.048896

O 1.950583 -0.311248 1.054352

O 1.883142 -0.283711 -1.155448

N -1.549018 -0.262224 -0.084857

O -1.712421 0.214421 1.111034

O -2.037135 0.179769 -1.070909

O 0.480513 1.619545 0.018224

H 0.319668 2.249480 -0.695995

H -0.152563 1.853269 0.711334

12

ss5d + 1h2o

N -0.615802 -1.178708 0.178451

H -0.743865 -1.852445 -0.583316

H -0.982206 0.310061 1.733249

N 1.856073 -0.275173 0.138840

O 1.790359 0.141456 1.162099

O 2.014893 -0.738186 -0.853211

N -1.532041 -0.332228 0.057838

O -1.565544 0.623109 1.018665

O -2.383514 -0.218837 -0.781826

O 0.550424 1.467240 -0.768659

H 0.462776 1.703042 -1.701038

H 0.052734 2.143853 -0.291346

12

ts6d + 1h2o

N -0.353291 -0.778719 -0.855984

H -0.404194 -0.469779 -1.833218

H -0.885247 -1.397977 1.210178

N 1.533367 -0.345016 -0.057848

O 1.461787 -0.696359 1.008953

O 2.055766 0.052196 -0.970020

N -1.343277 -0.280294 -0.231168

O -1.411364 -0.587520 1.069336

O -2.204925 0.422371 -0.646341

O 0.336411 1.704312 0.460921

H 0.324246 2.490681 -0.100219

H 0.206215 2.045277 1.355474

12

ss7d + 1h2o

N 0.009910 -0.730087 -0.634356

H 0.022036 -0.668632 -1.661472

H -0.247211 -0.226025 1.523645

N 1.238692 -0.365703 -0.013385

O 1.212927 -0.229541 1.189591

O 2.152956 -0.304205 -0.744249

N -1.178624 -0.390673 -0.078508

O -1.225473 -0.233230 1.185150

O -2.130984 -0.342315 -0.744294

O -0.030104 1.899353 -0.175712

H -0.093345 2.417283 -0.989009

H -0.005895 2.562121 0.526702

12

ts8d + 1h2o

N -0.075386 -0.853328 -0.329666

H -0.091667 -1.506588 -1.122292

H -0.386099 0.934058 1.087188

N 1.215556 -0.457286 0.157855

O 1.233472 0.272374 1.105130

O 2.096889 -0.952872 -0.443761

N -1.250666 -0.354761 0.045216

O -1.302697 0.556265 0.951509

O -2.233130 -0.772893 -0.426265

O 0.276130 1.833973 -0.806979

H -0.248913 2.225564 -1.517324

H 0.934842 2.509809 -0.598474

12

ss9d + 1h2o

N 1.072619 -0.136033 -0.435947

H 2.087661 -0.274187 -0.464570

H -1.896287 -0.182550 -0.070275

N 0.735097 1.158475 0.029680

O -0.398468 1.367860 0.346480

O 1.649704 1.917962 -0.004062

N 0.412461 -1.275195 0.018926

O -0.774737 -1.218184 0.254225

O 1.090474 -2.247444 0.077461

O -2.745046 0.335871 -0.285065

H -3.543740 -0.192500 -0.090488

H -2.764295 1.169974 0.224410

15

ss3d + 2h2o

N -0.368794 1.760827 -0.048240

H -0.446706 2.316372 -0.900203

H -0.563875 2.276036 0.811664

N -1.948970 -0.435617 0.261320

O -1.475925 -0.606642 1.249283

O -2.557150 -0.236266 -0.642844

N 0.863232 1.166517 0.021757

O 1.195295 0.749725 1.110229

O 1.479446 1.032414 -1.000667

O -0.182147 -1.274670 -0.901689

H 0.000181 -1.008785 -1.812111

H 0.699383 -1.530415 -0.533879

O 2.267290 -1.702011 0.111657

H 2.307698 -2.349914 0.827432

H 2.370567 -0.845778 0.555487

15

ts4d + 2h2o

N -0.170783 1.787469 0.194586

H -0.476594 2.469163 -0.502306

H 0.204307 1.328497 1.359218

N -2.012642 -0.340463 0.211500

O -1.591552 -0.648226 1.189928

O -2.568404 -0.000010 -0.683944

N 0.949939 1.179176 -0.046424

O 1.252158 0.633584 1.100140

O 1.598365 1.086108 -1.034346

O -0.251979 -1.153089 -0.953849

H -0.087528 -0.999573 -1.892488

H 0.584128 -1.542874 -0.596395

O 2.083266 -1.902484 0.098939

H 2.060921 -2.646321 0.715271

H 2.174341 -1.119236 0.664122

15

ss5d + 2h2o

N 0.265741 -1.656224 -0.214168

H 0.317935 -1.986629 -1.183707

H -0.483997 -1.081130 1.769231

N 2.080550 0.266692 0.214091

O 1.721107 0.433764 1.248482

O 2.564026 0.069721 -0.761964

N -0.863827 -1.145684 -0.071860

O -1.136454 -0.674878 1.168951

O -1.752386 -0.970681 -0.871233

O 0.271476 1.347246 -0.725151

H 0.202419 1.484259 -1.678368

H -0.614609 1.596699 -0.370722

O -2.278704 1.708043 0.091022

H -2.335313 1.565147 1.045442

H -2.576203 0.862446 -0.279179

15

ts6d + 2h2o

N 0.712613 -1.323665 -0.287148

H 0.864022 -1.562762 -1.274365

H -0.168138 -1.046286 1.744018

N 1.851632 0.275343 0.196160

O 1.596779 0.511346 1.271614

O 2.466788 0.420143 -0.738824

N -0.535142 -1.139676 -0.102679

O -0.887350 -0.787769 1.133936

O -1.417673 -1.228065 -0.891898

O -0.076257 1.451808 -0.643928

H -0.112444 1.713924 -1.572146

H -1.006372 1.516027 -0.334058

O -2.683194 1.270062 0.114600

H -2.798938 1.175542 1.069451

H -2.974595 0.419357 -0.241224

15

ss7d + 2h2o

N 0.859402 -0.859452 -0.525476

H 0.913814 -0.869642 -1.552879

H 0.210542 -0.249781 1.516546

N 1.728376 0.101293 0.066824

O 1.560083 0.322548 1.245219

O 2.559690 0.514859 -0.650247

N -0.418210 -0.986630 -0.070496

O -0.643247 -0.712775 1.152314

O -1.233112 -1.428355 -0.770925

O -0.355241 1.541419 -0.433939

H -0.255881 2.082910 -1.226154

H -1.320651 1.496780 -0.270043

O -2.870458 0.756918 0.074190

H -3.311736 0.996214 0.899479

H -3.564795 0.800123 -0.595807

15

ss9d + 2h2o

N 0.836024 0.647982 0.517075

H 1.413540 1.389040 0.925399

H -0.658967 -1.777470 -0.405947

N 1.620644 -0.461488 0.157684

O 1.076390 -1.386811 -0.388290

O 2.758313 -0.397371 0.502230

N -0.092699 1.189098 -0.409031

O -0.576756 0.456329 -1.218227

O -0.320374 2.342623 -0.216381

O -1.601738 -1.940815 -0.163693

H -2.099537 -2.136499 -0.976031

H -1.985757 -1.040105 0.339327

O -2.296969 0.096321 0.955735

H -3.058537 0.566133 0.579533

H -2.469452 0.007545 1.906633

21

ss3d + 4h2o

N -1.004302 0.566117 -1.365645

H -1.359441 0.345274 -2.294142

H -1.406400 1.360115 -0.826430

N -1.840212 -1.252928 0.458525

O -2.149922 -0.440868 1.149226

O -1.753359 -2.099177 -0.255296

N 0.345259 0.598217 -1.349782

O 0.878584 1.229143 -0.463116

O 0.935177 -0.050671 -2.185779

O 0.299581 -1.198561 0.998164

H 1.072459 -1.452293 0.443352

H 0.677416 -0.464570 1.518162

O 2.179722 0.635013 1.878129

H 2.619781 0.789525 2.724136

H 2.048603 1.509877 1.487891

O 2.704437 -1.163301 -0.218428

H 2.817612 -0.467915 0.454702

H 2.512928 -0.676306 -1.033766

O -1.662309 2.328657 0.590737

H -0.762791 2.610958 0.808174

H -2.180673 3.143612 0.567140

21

ts4d + 4h2o

N -1.093848 -0.305855 0.968276

H -1.811294 -0.249249 1.708464

H -0.558734 -1.238920 0.944045

N -1.997194 -0.114581 -0.616487

O -2.029329 -1.155488 -1.096517

O -2.366634 0.962102 -0.721451

N -0.141334 0.764605 1.166459

O 0.986612 0.412259 1.318431

O -0.598599 1.861806 1.144725

O 0.207875 0.402112 -1.451640

H 0.818595 1.154070 -1.321493

H 0.849125 -0.329606 -1.503123

O 2.400972 -1.110057 -0.747253

H 2.954266 -1.743763 -1.221213

H 1.933538 -1.627013 -0.067661

O 2.492397 1.702674 -0.675692

H 2.698672 0.753561 -0.585221

H 2.393334 1.998801 0.237974

O 0.463753 -2.462269 0.862040

H 0.733056 -2.783403 1.736887

H 0.159688 -3.248769 0.382461

21

ss5d + 4h2o

N 1.730974 -0.723713 0.130774

H 2.600638 -1.260178 0.147445

H 0.448989 0.715910 2.661837

N 1.852181 0.532422 -0.477643

O 2.968425 0.955634 -0.433735

O 0.884583 1.056867 -0.944536

N 0.584488 -1.505981 0.061330

O -0.476139 -0.952756 -0.049295

O 0.789095 -2.676344 0.187671

O -1.837151 1.407555 0.696724

H -1.723883 1.329980 -0.345926

H -2.380320 0.585139 0.855163

O -3.159671 -0.761606 0.203659

H -4.075950 -0.991649 0.409299

H -2.643685 -1.570943 0.331681

O -1.835194 0.878106 -1.720732

H -2.401752 0.100977 -1.566692

H -0.964410 0.515211 -1.943293

O 0.428993 1.223619 1.837264

H 0.794230 2.090970 2.067686

H -0.930896 1.316894 1.165413

21

ss4e (hno3 formation)

N 0.476226 2.519518 -0.305305

H 1.008114 2.976241 -1.040042

H -0.544252 2.507063 -0.281938

N -0.284153 -1.263354 -0.935611

O -1.433812 -1.059316 -1.237309

O 0.688096 -0.940471 -1.559381

N 1.028997 1.420630 0.180923

O 0.334914 0.696878 0.872012

O 2.203548 1.204671 -0.058740

O -0.100844 -1.895817 0.216928

H 0.971717 -1.768762 0.492719

H -1.290878 -1.084781 1.215762

O -2.016165 -0.482886 1.533578

H -2.835893 -1.003505 1.599144

H -2.144463 0.326419 0.827620

O 2.235090 -1.506774 0.784284

H 2.395642 -1.518869 1.739844

H 2.408137 -0.584752 0.496520

O -2.294119 1.318373 -0.126640

H -3.099169 1.853342 -0.031862

H -2.350093 0.882763 -0.995676

3.4 Frequency tables for calculations with explicit solvent

Table S5: Frequencies for all stationary states and transition states for calculations with explicit solvent. Calculated with M06-2X/def2-SVP/PCM

		(SS3d + 1h2o)			
29.7753	84.4564	91.1549	131.2831	150.1527	179.8690
196.7340	225.4092	254.5702	296.6893	411.6970	506.8212
559.5509	602.1605	621.4659	720.5608	788.1363	884.7901
1086.9768	1267.1533	1506.7916	1577.3984	1592.9287	1623.4453
1795.0685	2606.9667	3519.8799	3639.5371	3838.6464	3920.7357

		(TS4d + 1h2o)			
-1963.2334	40.7426	71.8034	88.6118	103.4489	118.6249
154.3522	185.0817	202.8881	256.2742	319.3001	398.2579
564.5658	590.6394	618.5501	657.7080	758.6204	856.3031
1100.7329	1146.0375	1291.1155	1468.5786	1577.3413	1615.4916
1756.0468	2322.0958	2606.0435	3558.7884	3834.8120	3920.2724

		(SS5d + 1h2o)			
40.8806	79.9106	98.5342	131.5496	142.7881	160.9330
190.5726	206.0903	218.5266	283.0036	370.8811	518.7647
571.3148	587.8808	638.0428	697.1212	784.1108	866.2783
988.7212	1341.3065	1361.6066	1526.5587	1577.5725	1613.7148
1794.2807	2616.6392	3515.2418	3763.9925	3849.2387	3934.8315

		(TS6d + 1h2o)			
-279.2439	60.3525	84.1391	119.4882	142.9431	148.3139
156.2455	199.4896	233.4803	286.7460	334.8933	430.1468
535.5126	617.7346	647.1465	703.7710	792.7771	863.6410
1015.9510	1327.5312	1360.4902	1450.2746	1540.0848	1610.8721
1798.8866	2402.4056	3506.1463	3729.9369	3850.9458	3933.0372

		(SS7d + 1h2o)			
103.5262	110.3250	160.6312	166.0278	224.9786	268.2259
307.9818	313.6948	373.9092	471.7232	487.3794	635.1273
723.8441	757.4740	774.1145	862.7911	893.5881	981.9811
1053.3992	1231.6647	1449.0327	1480.4867	1567.4101	1629.8742
1861.5018	1948.9463	2675.3352	3481.1073	3852.8432	3930.8546

		(TS8d + 1h2o)			
-381.7052	44.2195	82.1607	152.7350	203.8848	218.0660
274.3019	281.2516	335.3039	390.5911	439.8450	452.9600
478.5710	747.1139	755.7394	794.3945	853.7542	956.5108
1060.1582	1279.3974	1438.6211	1461.6067	1505.2831	1634.3050
1863.9082	1921.6687	3291.2137	3490.8408	3847.7706	3927.4798

		(SS9d + 1h2o)			
47.3817	83.8086	115.1768	155.6922	207.0505	231.4147
288.8051	390.4785	449.1855	483.2383	540.2950	634.1239
791.7713	802.0634	817.3866	886.8648	1008.5456	1055.0406
1082.0698	1374.5663	1432.7405	1516.9688	1611.9672	1639.6824
1816.7992	1867.8488	2892.2076	3522.7844	3715.9842	3782.2656

		(SS3d + 2h2o)			
55.0530	68.1734	87.3347	102.7955	118.4517	156.4148
160.5091	171.4237	194.4932	246.8768	257.0822	323.5468
336.6400	384.8670	468.5951	502.4324	536.0786	593.7166
608.0606	618.5746	720.6585	777.2518	864.9864	931.8295
1098.1641	1259.4592	1513.7346	1565.4767	1587.9344	1615.0597
1655.1871	1788.6468	2597.0333	3366.0235	3510.0597	3634.7640
3806.2338	3891.5268	3908.3593			

(TS4d + 2h2o)					
-1943.0040	49.6177	58.2326	76.7957	86.0261	109.3110
136.4302	143.0081	168.2458	188.7007	253.1695	272.2282
300.1564	325.8761	335.4152	441.5476	535.0883	578.2539
588.9816	612.8459	644.9409	758.1230	856.1098	879.5025
1107.5661	1140.1956	1292.1121	1471.5445	1566.8361	1617.9706
1642.0874	1764.7018	2321.0593	2599.1025	3344.8025	3553.8204
3798.5278	3902.8214	3907.3796			

(SS5d + 2h2o)					
55.9868	87.9388	100.1247	123.8676	131.1838	146.6993
160.8866	183.4565	204.3916	241.3442	254.2714	287.4615
328.8631	402.9539	424.2063	472.6015	550.6675	605.0155
633.9351	640.1930	694.1316	777.3296	862.4389	881.8686
987.3818	1323.8892	1359.6544	1522.6964	1572.2806	1626.7214
1672.6691	1781.9800	2608.7426	3421.1725	3511.5599	3751.9371
3811.8976	3895.6628	3903.8408			

(TS6d + 2h2o)					
-274.4972	46.3597	82.557	97.0234	127.8267	137.558
147.2198	157.6191	182.243	210.3812	226.7138	292.574
318.0475	347.2755	365.407	481.3451	556.1834	575.561
623.8095	654.8494	705.835	772.0659	801.5818	854.353
1022.1761	1321.4403	1365.588	1438.4572	1536.5037	1613.023
1655.1502	1795.6626	2345.276	3490.7542	3516.7237	3717.568
3843.3374	3899.0101	3923.493			

(SS7d + 2h2o)					
60.7814	84.6895	85.1067	127.7741	166.8993	184.3141
188.3516	226.1782	252.5024	263.5786	299.3355	321.5978
323.8515	475.1878	494.0827	554.7012	636.3455	722.7593
728.4539	764.1799	781.7959	862.5549	871.1002	987.5679
1053.5880	1240.4599	1462.6940	1486.7099	1582.7835	1605.4401
1651.9274	1863.6216	1952.4958	2628.9817	3485.8606	3565.0794
3854.9918	3908.4792	3940.2767			

(SS9d + 2h2o)					
46.1283	68.7368	82.9329	125.3346	134.2582	164.7446
185.1813	263.7697	277.9884	336.9180	413.0825	450.8845
463.0493	485.3900	497.9687	648.7558	662.8749	709.2360
789.4864	809.8154	821.2880	890.4511	996.8722	1076.1165
1299.2962	1394.6327	1429.7972	1514.9285	1556.4115	1602.3192
1710.4732	1825.7629	1857.0126	1870.1335	3493.7365	3527.0792
3793.6762	3807.6098	3872.9601			

(SS3d + 4h2o)					
38.7483	52.9320	64.7185	69.2316	89.7756	106.7164
109.3803	131.1586	136.0791	149.6798	168.9412	174.6169
179.3938	189.5333	222.3486	232.9753	247.1681	264.2956
275.8564	290.8890	324.9710	366.5850	385.1151	449.5854
461.8787	518.8590	534.6674	596.4006	606.4579	635.4535
641.0707	722.9825	750.8442	809.5696	852.5824	867.9962
1123.9912	1302.1727	1515.9329	1552.2298	1575.6348	1593.0011
1609.5261	1627.0725	1693.6559	1763.1429	2560.3654	3154.9132
3488.7013	3616.7203	3698.6856	3726.0107	3837.0873	3838.7057
3848.0704	3926.7189	3933.6668			

(TS4d + 4h2o)					
-140.6692	29.8017	56.9045	59.5651	85.8797	109.6695
138.6133	142.1929	160.8473	170.2282	198.6671	207.4084
209.6969	223.9996	228.2638	250.8648	281.3057	317.2009
331.9761	369.0718	384.4176	393.5761	437.6036	456.3297
486.7544	553.9668	587.4922	606.0965	657.4375	676.0129
712.2538	740.4243	807.3933	827.6691	909.8592	972.1914
1243.5521	1407.6362	1442.1334	1496.7707	1550.3619	1595.4326
1618.2272	1630.5146	1703.8071	1875.7623	2179.1383	2603.2173
3441.9291	3646.7758	3699.7616	3713.5504	3757.8264	3805.9388
3880.3181	3899.7993	3906.3715			

(SS5d + 4h2o)					
28.1083	42.1663	51.8756	66.7446	89.0989	106.7700
120.7682	133.9664	139.6920	160.8938	173.6663	261.9501
274.4985	277.2574	285.0766	317.7960	352.9017	370.8974
407.0180	415.0234	437.1428	449.5301	461.9336	483.1889
501.8926	521.3956	595.3544	780.2227	791.1658	800.7102
808.1518	852.7094	881.9944	947.6085	1042.1668	1073.1095
1347.9694	1393.9251	1415.4729	1516.8993	1576.7575	1606.5873
1610.8613	1664.0491	1743.6904	1811.4850	1853.8669	2398.1245
2779.0432	3290.4342	3563.3582	3748.3245	3822.4181	3831.1368
3859.7765	3901.4319	3919.8018			

(SS4e - hno3 formation)					
53.1035	69.9235	95.3110	107.8419	116.7165	130.3943
140.0788	148.9369	154.4958	174.8231	186.8778	195.9997
223.1988	291.5104	304.0316	330.8981	408.6874	418.5376
439.1652	517.9150	530.7817	560.4430	609.4587	638.8077
663.4266	715.6753	740.3826	757.2890	797.9539	829.8082
848.0048	878.1919	1069.7574	1156.7827	1215.8157	1237.2675
1284.8775	1358.1161	1530.1003	1553.3331	1582.1915	1593.9559
1625.1664	1665.2274	1699.8350	1705.4126	1764.7706	1854.3897
2036.6636	3332.9672	3507.8987	3599.3809	3687.0139	3762.8780
3796.1066	3847.4533	3859.5785			

3.5 Table of energies for the mechanisms calculated with explicit solvent

Table S6: Relative energies (kcal mol^{-1}) for calculations with $1H_2O$. Calculated with M06-2X/def2-SVP/PCM

Species	ΔE	ΔH(0K)	ΔH(298.15K)	ΔG(298.15K)
$SS3_D$	0.00	0.00	0.00	0.00
$TS4_D$	44.88	41.26	41.29	41.06
$SS5_D$	12.44	12.09	12.21	12.07
$TS6_D$	15.75	14.57	14.30	15.25
$SS7_D$	5.55	4.65	3.60	6.73
$TS8_D$	6.66	5.91	4.99	7.40
$SS9_D$	-13.75	-12.98	-13.98	-11.74

Table S7: Relative energies (kcal mol^{-1}) for calculations with $2H_2O$. Calculated with M06-2X/def2-SVP/PCM

Species	ΔE	ΔH(0K)	ΔH(298.15K)	ΔG(298.15K)
$SS3_D$	0.00	0.00	0.00	0.00
$TS4_D$	45.59	41.79	41.88	41.33
$SS5_D$	12.97	12.85	12.83	13.13
$TS6_D$	18.35	17.16	16.94	17.50
$SS7_D$	7.91	7.43	6.86	8.41
$SS9_D$	-23.39	-23.57	-24.51	-22.69

Table S8: Relative energies (kcal mol^{-1}) for calculations with $4H_2O$. Calculated with M06-2X/def2-SVP/PCM

Species	ΔE	ΔH(0K)	ΔH(298.15K)	ΔG(298.15K)
$SS3_D$	0.00	0.00	0.00	0.00
$TS4_D$	3.66	-7.77	2.77	5.42
$SS5_D$	-33.10	-44.12	-33.68	-31.55
$SS5_E$	-39.80	-50.48	-40.86	-36.06
